# The epigenomic landscape of transposable elements across normal human development and anatomy

**DOI:** 10.1038/s41467-019-13555-x

**Published:** 2019-12-10

**Authors:** Erica C. Pehrsson, Mayank N. K. Choudhary, Vasavi Sundaram, Ting Wang

**Affiliations:** 1 Department of Genetics, Washington University School of Medicine, 4515 McKinley Avenue, St. Louis, MO 63110 USA; 2 The Edison Family Center for Genome Sciences and Systems Biology, Washington University School of Medicine, St. Louis, MO 63110 USA; 3 European Molecular Biology Laboratory, European Bioinformatics Institute, Wellcome Genome Campus, Hinxton, Cambridge, CB10 1SD UK; 4 McDonnell Genome Institute, Washington University School of Medicine, St. Louis, MO 63108 USA

**Keywords:** Bioinformatics, Epigenetics analysis, Epigenomics, Gene regulation, Epigenetics

## Abstract

Transposable elements (TEs) have deposited functional regulatory elements throughout the human genome. Although most are silenced, certain TEs have been co-opted by the host. However, a comprehensive, multidimensional picture of the contribution of TEs to normal human gene regulation is still lacking. Here, we quantify the epigenomic status of TEs across human anatomy and development using data from the Roadmap Epigenomics Project. We find that TEs encompass a quarter of the human regulatory epigenome, and 47% of elements can be in an active regulatory state. We demonstrate that SINEs are enriched relative to other classes for active and transcribed marks, that TEs encompass a higher proportion of enhancer states in the hematopoietic lineage, and that DNA methylation of Alu elements decreases with age, corresponding with a loss of CpG islands. Finally, we identify TEs that may perform an evolutionarily conserved regulatory function, providing a systematic profile of TE activity in normal human tissue.

## Abstract (web summary)

Although most are silenced, certain transposable elements (TEs) have been co-opted by the host. Here, the authors quantify the epigenomic status of TEs using data from the Roadmap Epigenomics Project, provide a systematic profile of TE activity across normal human tissues and development, finding that TEs encompass a quarter of the human regulatory epigenome, with 47% of TEs in regulatory states.

## Introduction

Transposable elements (TEs) are repetitive sequences that comprise approximately half of mammalian genomes due to historic proliferation within the host. Although the vast majority of human TEs have lost the ability to transpose, many encode functional regulatory elements that can disrupt gene regulatory networks if left unchecked. In consequence, most TEs are epigenetically silenced by DNA methylation and/or repressive histone modifications in normal somatic tissues^1,2^. For this reason, this sizable component of the genome was excluded from many genomic analyses in the past.

However, research from the past decade has demonstrated that a subset of TEs has been co-opted by the host and performs important roles in normal gene regulation and development. As such, TEs are now recognized as a rich source of genetic material for host regulatory innovation. TEs have contributed substantially to the propagation of transcription factor binding sites^3–5^, encoding 20% of the binding sites for 26 transcription factors profiled by ENCODE^6^, and they can disseminate batteries of synergistic transcription factor binding sites, suggesting that TEs provide the cell with a mechanism of efficiently coordinating gene regulatory networks^7,8^. Certain TE subfamilies (a collection of TE copies that descended from a single insertion into the ancestral genome) have been co-opted to rewire gene regulatory networks involved in pregnancy and innate immunity^9,10^, and they help disseminate CTCF (CCCTC-binding factor) binding sites and establish chromatin boundaries^11,12^. They can also act as tissue-specific enhancers^1^, in addition to their extensive contributions to the transcriptome in both normal and diseased tissues^13,14^. Overall, 18–31% of human transcription start sites derive from TEs^15,16^, 35% of which have highly restricted temporal and spatial expression, and 44% of open chromatin regions overlap TEs^17^.

Although many examples of TE exaptation have been published, a comprehensive profile of their epigenetic state and gene regulatory contribution is not yet complete. Analyses based on a single data type (e.g., transcription start sites^15,16,18^) or on a particular tissue capture only one dimension of the activity profile of TEs. However, large databases of epigenetic measurements across tissues are now available. For example, the Roadmap Epigenomics Project^19^ interrogated the epigenomes of 127 human tissues and cell types using complementary techniques, including ChIP-seq (chromatin immunoprecipitation) on numerous histone modifications (combined into a composite epigenetic state assignment using chromHMM), whole genome bisulfite sequencing (WGBS), DNase hypersensitivity (DHS) assays, and RNA sequencing. Although this dataset has been used to explore the *cis*-regulatory activity of TEs across tissues^20^, a thorough profile of TE contribution to gene regulation across multiple phylogenetic resolutions, using complementary epigenetic measurements, and in rarely profiled tissues is not yet complete.

Here, we use the dense, multidimensional data generated by the Roadmap Project and other consortia^21–23^ to create a quantitative epigenetic profile of human TEs across a wide breadth of normal tissues and multiple stages of development. By overlaying these datasets on the 4.4 million TEs in the human genome, we show that half of TEs exhibit biochemical activity typical of a gene regulatory element in at least one of the Roadmap epigenomes. We explore the epigenetic dynamics of tissue-specific active TEs and demonstrate both tissue- and phylogeny-specific variations in TE epigenetic marks that have potential functional implications. Finally, we investigate alterations in TE repression mechanisms over time and explore the conservation of TE regulatory signatures across species. Together, these analyses provide a systematic profile of TE activity across normal human tissues.

## Results

### Substantial contribution of TEs to the regulatory epigenome

**Fig. 1:**
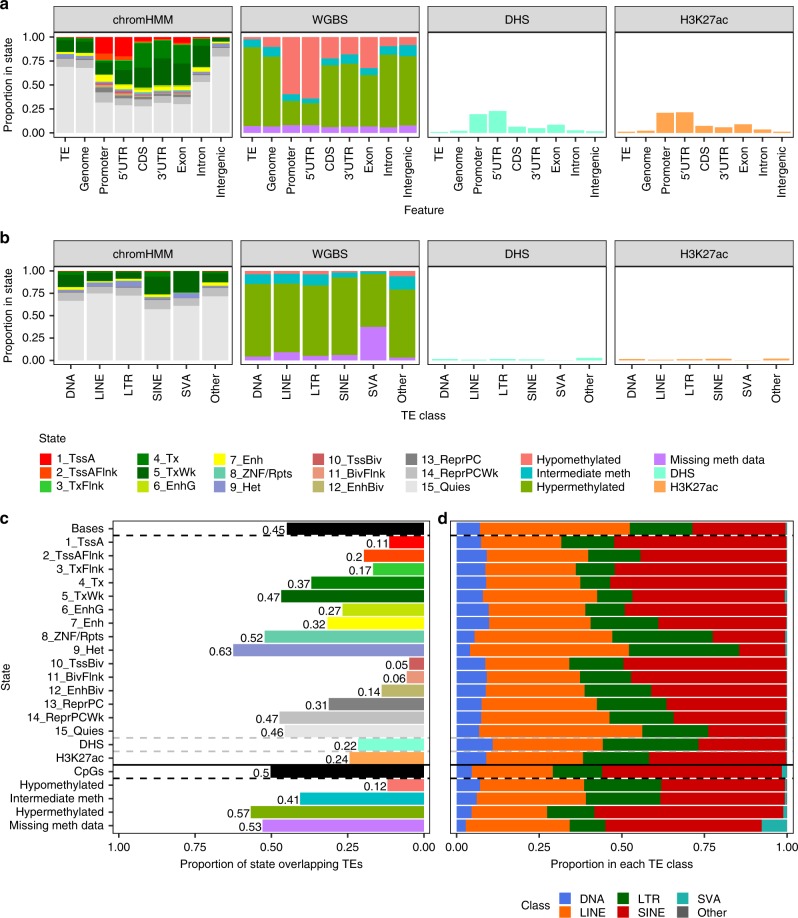
Contribution of TEs to epigenetic states across all Roadmap epigenomes. **a** Proportion of bases within TEs, the entire genome, and RefSeq genic features annotated with each chromHMM state, methylation state (proportion of CpGs), and overlapping DHS or H3K27ac peaks, summed across all epigenomes with data for that technique (chromHMM states 127 epigenomes, methylation states 37, DHS 53, and H3K27ac 98). The color legend is below **b**. Methylation states are defined as: hypomethylated, <30% methylated; intermediately methylated, 30–70%; hypermethylated, >70%; missing methylation data, ≤3 reads covering the CpG. CDS: coding exon; UTR: untranslated region. **c** Proportion of bases within each TE class annotated with each epigenetic state, summed across all epigenomes with data for that technique. **c** Total proportion of the epigenetic state within TEs across all epigenomes with data for that technique vs. the total proportion of all genomic bases and CpGs within TEs. Bars are colored by state (*y*-axis). **d** The proportion of each bar in **c** by TE class. The *y*-axis is shared with **c**.

For an initial, high-level epigenetic profile of TEs across human tissues, we first identified the total proportion of TE bases or CpGs annotated with each epigenetic state across all Roadmap epigenomes (Fig. 1a; Supplementary Fig. 1). In comparison to genes (Supplementary Fig. 2a), TEs are depleted in active regulatory (1_TssA, 2_TssAFlnk, 3_TxFlnk, 6_EnhG, 7_Enh) and transcribed (4_Tx, 5_TxWk) chromHMM states (Supplementary Table 1), hypomethylated CpGs (<30% methylated), and DHS and H3K27ac (histone 3 lysine 27 acetylation) peaks. Indeed, only 3% of TE bases are annotated with active regulatory chromHMM states, compared to 32% of promoter bases, and only 15% are annotated with transcribed states, compared to 42% of exon bases. This pattern is effectively identical when considering the average TE profile across all epigenomes (Supplementary Fig. 2b) and is even more striking for protein-coding vs. non-coding genes (Supplementary Fig. 2c).

The aggregate epigenetic profile of TEs also differs by class, which includes DNA transposons, LINE (long interspersed nuclear element), SINE (short interspersed nuclear element), and LTR (long terminal repeat) retrotransposons, the primate-specific SVA class (SINE-VNTR-Alu), and Other class TEs, older elements whose sequence is too degraded to accurately classify (Fig. 1b; Supplementary Fig. 3a–c). For instance, the SINE class has a higher proportion of bases in the 4_Tx transcribed state and most active regulatory states than other classes, while the SVA class has more CpGs missing methylation data. The latter is likely due to SVA length (median 1,151.5 bp vs. 232 bp for all TEs; Supplementary Fig. 3d) and recent propagation in the genome, which decreases mappability (i.e., the likelihood that a read is correctly mapped). Indeed, the number of epigenomes in which individual SVA elements are missing methylation data is negatively correlated with mappability (Spearman correlation, *ρ* = −0.38, *P*-value < 0.001). Interestingly, a higher proportion of the LTR class is annotated with the 9_Het heterochromatin state, but the SINE class is more hypermethylated than other classes, suggesting that the two classes are subject to different mechanisms of epigenetic repression. Regression analysis on individual TEs confirms that this difference is driven primarily by TE class, not CpG density (Supplementary Discussion; Supplementary Table 2).

Despite their depletion in active epigenetic states, TEs have enormous representation in the genome, encompassing ~45% of its length and ~50% of CpGs. As a result, TEs comprise 26% of active regulatory and 44% of transcribed chromHMM states across all Roadmap epigenomes, as well as 22% of DHS peaks and 24% of H3K27ac peaks (Fig. 1c). Some TE classes contribute a disproportionate amount of each epigenetic state (Fig. 1d). For instance, the SINE class contributes 37–53% of the active and poised (10_TssBiv, 11_BivFlnk, 12_EnhBiv) chromHMM states and 41% of H3K27ac peaks within TEs, although it encompasses only 28% of TE bases. Therefore, although TEs are depleted in active epigenetic states, their contribution to them is substantial, and the extent of the contribution varies by TE class.

### Potential for individual TEs to be epigenetically active

**Fig. 2:**
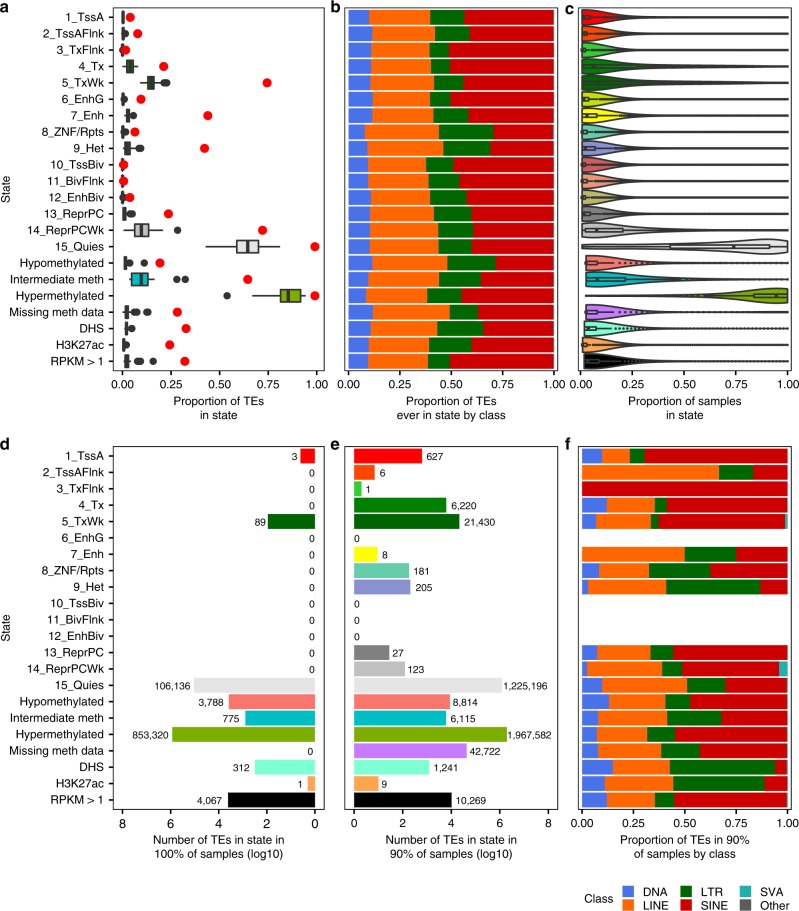
Individual TE potential to be annotated with an epigenetic state. **a** Boxplots indicate the proportion of all TEs (4,430,788 TEs) annotated with the state per epigenome (chromHMM states *n* = 127 epigenomes, methylation states *n* = 37, DHS *n* = 53, H3K27ac *n* = 98, expression RPKM >1 *n* = 56; see Methods). Red dots are the fraction of TEs annotated with the state in at least one epigenome. For WGBS states, only TEs with CpGs are included (3,200,428 TEs, 72% of all TEs). **b** For TEs annotated with the state in at least one epigenome (**a**, red dots), the proportion in each TE class. The color legend is located below **f**. **c** For TEs annotated with the state in at least one epigenome (**a**, red dots), the proportion of all Roadmap epigenomes the TE is annotated with the state. The **b** and **c** *y*-axes are shared with **a**. **d** Number of TEs annotated with the state in 100% of epigenomes. **e** Number of TEs annotated with the state in ≥90% of epigenomes. **f** For TEs annotated with the state in ≥90% of epigenomes, the proportion in each TE class. The **e** and **f** *y*-axes are shared with **d**. **a**, **c**–**e** Boxplots, bars, and distributions are colored by state (*y*-axis). **a**, **c** Boxplot elements: center line, median; box limits, first and third quartiles; whiskers, maximum value ≤1.5*IQR from box limits; points, outliers.

We next investigated the likelihood of each of the 4,430,788 human TE fragments to be annotated with an active epigenetic state in a Roadmap tissue. Within each epigenome, only a small fraction of individual TEs are in each active epigenetic state (see Methods; median ≤3% for all active regulatory states and DHS/H3K27ac peaks; Fig. 2a). However, the majority of TEs are annotated with an active epigenetic state in a Roadmap epigenome: 47% of individual TEs are in an active regulatory chromHMM state and 75% are in a transcribed state at least once. Specifically, 4% of TEs are in the 1_TssA state (characterized by H3K4 tri-methylation, H3K4me3), 44% in the 7_Enh state, and 33% and 24% overlap a DHS or H3K27ac peak, respectively. In contrast, 82% of TEs are in a repressed state (heterochromatin, 9_Het, or Polycomb repressed, 13_ReprPC and 14_ReprPCWk) in at least one Roadmap epigenome.

Despite the extensive quality control performed on the Roadmap datasets, our results are dependent on variability between similar epigenomes, and some fraction of the 47% of TEs may be noise. The method of annotating TEs with chromHMM states also has a moderate impact on this result. When TEs are required to overlap the center of a 200 bp chromHMM annotation window, the statistic decreases to 37%, dropping further to 26% when TEs are required to overlap the center of a chromHMM annotation block (Supplementary Fig. 4a, b; Supplementary Discussion). Furthermore, although a TE must overlap the summit of a DHS or H3K27ac peak (the predicted binding location^24^) to be annotated with that state, we cannot say with complete certainty that the TE is responsible for the peak.

However, the results are robust to the chromHMM model used. With the 18-state model generated by the Roadmap Project, which includes H3K27ac as well as the modifications included in the 15-state model, the number of TEs in an active regulatory state (states 1–4 and 7–11) in at least one epigenome increases slightly to 48%, despite a 23% reduction in the number of epigenomes under consideration (*n* = 98; Supplementary Fig. 5a, b). The Roadmap Project also trained separate 50-state chromHMM models on seven deeply profiled reference epigenomes (embryonic stem cells (ESCs), ES-derived cells, and IMR90) using all available epigenetic marks. Using those state calls, a median of 8% of TEs are in an active regulatory state in each epigenome (based on the corresponding 18-state model state; Supplementary Fig. 5c), as opposed to 3% with the 15-state model. Therefore, more nuanced annotation models increase the observed regulatory role of TEs.

The fraction of individual TEs ever in an active regulatory state is slightly lower than expectation, as estimated by shuffling the locations of all TEs ten times. On average, 49% (standard deviation, 0%) of shuffled TEs are in an active regulatory state in at least one epigenome. However, the particular TEs in an active regulatory state in each epigenome are not random, and we observe subfamily-specific patterns that are lost with shuffling (see below).

Although LTR elements comprise 16% of TEs, they account for 27% of TEs ever in the 8_ZNF/Rpts state and 23% of those ever in the 9_Het heterochromatin state, as well as 21–22% of TEs overlapping DHS or H3K27ac peaks (Fig. 2b; Supplementary Fig. 6a). SINE elements (40% of all TEs) represent 50–51% of TEs ever in the transcribed 4_Tx state, the transcribed flanking 3_TxFlnk state, the genic enhancer 6_EnhG state, or expressed RPKM >1 (reads per kilobase per million reads), likely due to greater overlap with protein-coding introns (Supplementary Fig. 6b).

TE regulatory signatures in the Roadmap epigenomes are restricted rather than universal. A TE in an active regulatory state or overlapping a DHS or H3K27ac peak remains in that state in only 1–6% of epigenomes (median; Fig. 2c), while a TE remains in a transcribed chromHMM state in 6–9% of epigenomes. The degree of restriction also exhibits class-specific variation: for example, SINE and SVA elements remain in the 5_TxWk state longer than other TEs (Supplementary Fig. 6c). In contrast, RefSeq promoters and exons exhibit a much more universal activity profile (Supplementary Fig. 7a–d). 78% of promoters are in the 1_TssA state and >92% overlap a DHS and H3K27ac peak summit in at least one epigenome, while 93% of RefSeq exons are expressed RPKM >1 at least once (vs. 32% of TEs). A substantial proportion of promoters and exons (26–48%) also remain in those states in ≥90% of epigenomes. These results demonstrate that the ability of individual TEs to be regulatory elements, while greater than previously expected, is dramatically less than that of dedicated regulatory and genic elements.

To ensure that biological outliers did not skew our results, we repeated the analyses excluding five cancer cell lines and IMR90, which was identified as an epigenetic outlier in ref. ^19^ (Supplementary Fig. 7e, f). The results are very similar, except that the number of TEs hypo- or intermediately methylated in any epigenome decreases by 6–8% with the exclusion of IMR90, which has a much higher proportion of lowly methylated CpGs (Supplementary Fig. 1).

A small fraction of TEs is consistently annotated with the same epigenetic state in all epigenomes, and most are repressed (Fig. 2d). Of the 2% of TEs consistently in the same chromHMM state (*n* = 106,228), 99.9% are in the 15_Quies quiescent state, which lacks ChIP-seq signal for the five constituent histone modifications. However, there are a few examples of TEs in an active epigenetic state in all epigenomes (Supplementary Data 1; Supplementary Discussion), and even more in 90% of epigenomes (Fig. 2e). The latter are frequently enriched for SINE elements, although LTR elements are enriched among TEs overlapping DHS peaks (Fig. 2f). Many of these TEs overlap RefSeq promoters and genes, including 92% of TEs expressed RPKM >1 in all epigenomes (79% of which overlap protein-coding exons), but others are intergenic. This includes 13 TEs overlapping a DHS peak in all epigenomes that are >50 kb from the nearest RefSeq gene and do not overlap a GENCODE annotation. They are frequently in the 1_TssA promoter state, the 7_Enh enhancer state, or the 8_ZNF/Rpts state and may represent uncharacterized transcripts or enhancers.

### Epigenetic state dynamics of TEs

**Fig. 3:**
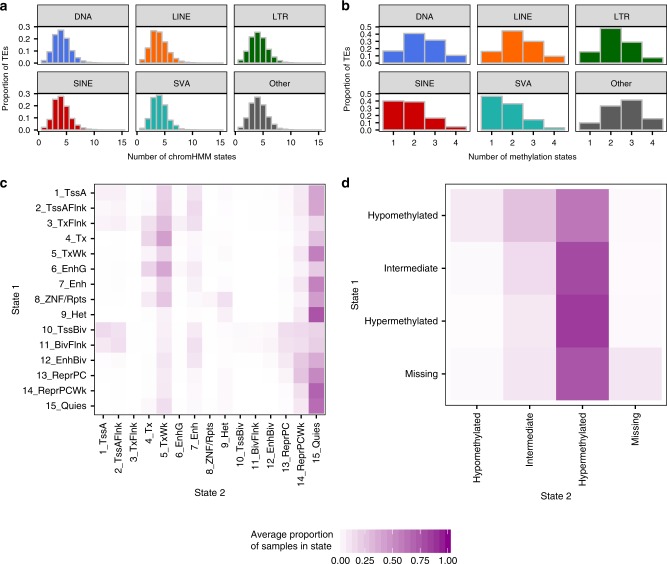
Epigenetic state dynamics of TEs. **a** For all individual TEs in each class, the total number of chromHMM states the TE is annotated with across all epigenomes (DNA *n* = 456,948 TEs, LINE *n* = 1,480,369, LTR *n* = 708,210, SINE *n* = 1,769,839, SVA *n* = 3,608, Other *n* = 11,814). **b** For all individual TEs in each class, the total number of methylation states the TE is annotated with across all epigenomes (TEs overlapping CpGs only: DNA *n* = 275,140 TEs, LINE *n* = 952,459, LTR *n* = 532,571, SINE *n* = 1,430,171, SVA *n* = 3,519, Other *n* = 6,568). **a, b** Histograms are colored by class (facet). **c** For TEs in chromHMM State 1 in at least one Roadmap epigenome (see Fig. 2a, red dots), the average proportion of epigenomes in which they are annotated with chromHMM State 2 (represented by color scale below **c**, **d**, 0 to 100% of epigenomes). **d** For TEs in methylation State 1 in at least one Roadmap epigenome (see Fig. 2a, red dots), the average proportion of epigenomes in which they are annotated with methylation State 2 (represented by color scale below **c**, **d**, 0 to 100% of epigenomes).

Because most TEs exhibit restricted regulatory activity, we next examined the dynamics of their epigenetic profiles across Roadmap epigenomes. Each TE is annotated with a median of four chromHMM and two methylation states across all epigenomes, although nine TEs are annotated with all 15 chromHMM states (Fig. 3a, b). However, the set of states with which each TE is annotated varies (Fig. 3c, d). For example, TEs in the poised promoter states (10_TssBiv and 11_BivFlnk) in any epigenome (<1% of TEs) spend an average of 10–16% of epigenomes in the corresponding active promoter and promoter flanking states (1_TssA and 2_TssAFlnk) and 13–15% of epigenomes in the Polycomb repressed state (13_ReprPC), a pattern typical of a poised regulatory element becoming active in a differentiating cell lineage. In contrast, the average TE spends ≤1% of epigenomes in those states.

Interestingly, TEs ever in repressed states are less dynamic than those ever in active states (Supplementary Fig. 8a, b). However, this may be partially due to long TEs that overlap multiple chromHMM states within a single epigenome (Supplementary Discussion; Supplementary Fig. 8c, d).

Although all TEs exhibit similar chromHMM dynamics regardless of class (Fig. 3a), SINE and SVA elements have less dynamic methylation profiles (Fig. 3b; Supplementary Fig. 8e). They are less likely to be hypo- or intermediately methylated in any epigenome (Supplementary Fig. 6a), and they remain intermediately methylated in fewer epigenomes (median 3–5% of epigenomes vs. 8–14% for other classes). This may be due to the tendency of SINE and SVA elements to overlap CpG islands (1% of SINE and 35% of SVA elements vs. <0.5% for other classes), which have greater coordination of CpG methylation levels and may result in a more bimodal distribution of average methylation. Taken together, these results suggest that some TEs have biochemical activity specific to particular tissues or developmental states, and that TE phylogeny contributes to this profile.

Compared to background (ten iterations of shuffled TEs), true TEs are slightly less dynamic than expected, although the results are almost identical (Supplementary Fig. 9a, b; Supplementary Discussion).

Finally, we confirmed the concordance of the five experimental techniques used to query TE epigenetic status (Supplementary Fig. 10a–e). TEs are significantly more likely to overlap a DHS or H3K27ac peak, be in an active regulatory chromHMM state, or be hypo- or intermediately methylated when also in one of the other states (pairwise by-technique Chi-squared tests, *P*-value = 0). In fact, although only 2% of TEs overlap a DHS peak and 0.7% overlap an H3K27ac peak (of the TE-by-epigenome instances with data for both metrics), 0.2% are annotated with both peaks, ten times the expected number if the peaks were independent. Furthermore, the proportion of TEs overlapping both peaks increases to 3–4% when the TE is also in the 1_TssA or 7_Enh state. However, because the epigenetic marks are not completely redundant, we have included all in our analyses.

### Differences in TE activity by tissue classification

**Fig. 4:**
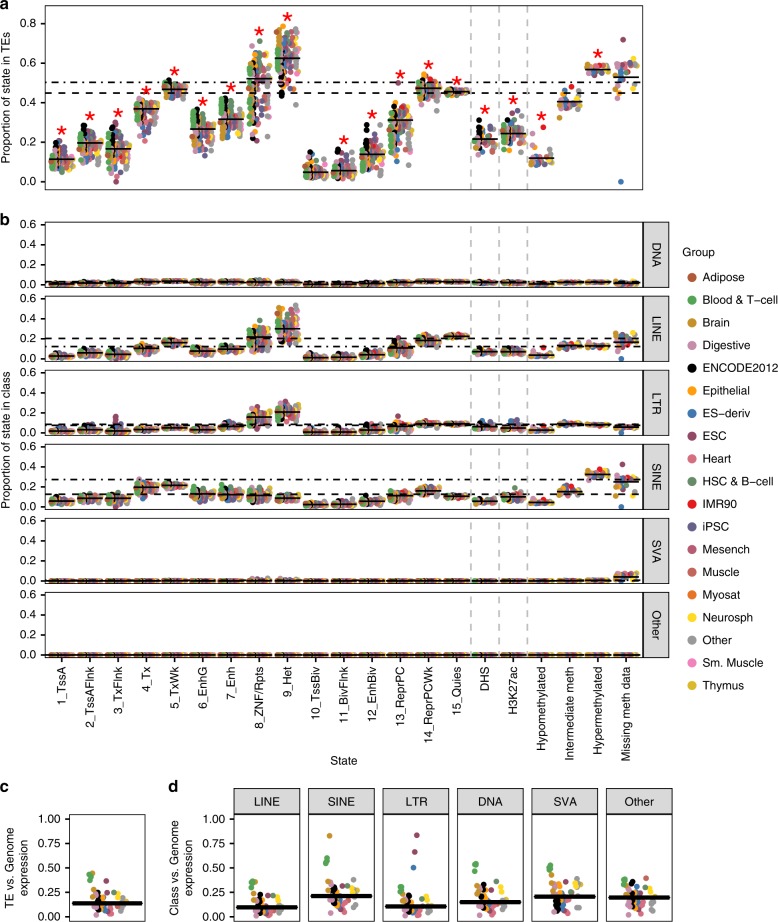
Variation in TE contribution to epigenetic states by epigenome classification. **a** Proportion of the epigenetic state within TEs by epigenome. Each epigenome is represented by a circle, colored by Roadmap group. Solid black lines represent the proportion of the epigenetic state within TEs across all epigenomes (contribution; see Fig. 1c). Dashed and dot-dashed lines represent the proportion of genomic bases and CpGs within TEs, respectively. Red stars represent Bonferroni-corrected Kruskal–Wallis test *P*-value < 0.05 across epigenome groups (chromHMM states *n* = 127 epigenomes, methylation states *n* = 37, DHS *n* = 53, H3K27ac *n* = 98; see Supplementary Data 3 for group assignments). **b** Proportion of the epigenetic state within each TE class by epigenome, colored by Roadmap groups. Solid black lines represent the proportion of the epigenetic state within the class across all epigenomes. Dashed and dot-dashed lines represent the proportion of genomic bases and CpGs within the TE class, respectively. **c** The ratio of the average RPKM over TEs to the average RPKM over the entire genome, colored by Roadmap group. **d** The ratio of the average RPKM over each TE class to the average RPKM over the entire genome, colored by Roadmap group. **c, d** The solid black bar is the median for all epigenomes (*n* = 56 epigenomes with RNA-seq data).

In our analyses across all Roadmap epigenomes, we established that TEs contribute different proportions of each epigenetic state to the genome (Fig. 1c), and that this pattern differs by TE class (Fig. 1d). Figure 4a demonstrates that there is also variation between epigenomes in the contribution of TEs to epigenetic states. Besides CpGs missing methylation data, the 8_ZNF/Rpts state exhibits the largest variation, ranging from 21% of the state within TEs (E002, ES-WA7 Cells) to 71% (E051, Primary hematopoietic stem cells G-CSF-mobilized Male). Indeed, TEs can be either enriched or depleted in this state compared to their genomic representation (dashed line) depending on the epigenome.

This variation across epigenomes is driven in part by tissue. The proportion of most epigenetic states within TEs is significantly different across the group classifications assigned by the Roadmap Project (*P*-value < 0.05, Kruskal–Wallis test, Bonferroni correction; Supplementary Discussion). As expected, ESCs are enriched among the samples with a higher contribution of TEs to the 1_TssA state than the total across epigenomes (solid black lines; ESC mean 16%; *P*-value < 0.1, permutation test with 1,000 permutations, FDR (false discovery rate) correction; Supplementary Data 2)^25–27^. Interestingly, blood groups (Blood & T-cell and HSC (hematopoietic stem cell) & B-cell) are also enriched among epigenomes with a higher contribution from TEs to the weakly transcribed (5_TxWk) and enhancer (6_EnhG and 7_Enh) states, while Brain epigenomes are depleted (mean 5_TxWk: 48–49% vs. 45%; 6_EnhG: 30–32% vs. 21%; 7_Enh: 37–38% vs. 25%). Other trends can be observed using additional Roadmap-assigned classifications (Anatomy and Type) or other epigenome metadata (e.g., donor age, germ layer of origin, and cancer cell lines; Supplementary Data 3), including that cancer cell lines are enriched among epigenomes with a higher proportion of many active and poised regulatory states in TEs (*P*-value < 0.05, Kruskal–Wallis test, Bonferroni correction; Supplementary Fig. 11).

The proportion of each epigenetic state within TE classes also shows tissue-specific patterns (Fig. 4b). For instance, only the LTR class shows enrichment of ESCs among epigenomes with a higher contribution of TEs to the 1_TssA, 2_TssAFlnk, 3_TxFlnk, 7_Enh, and H3K27ac states (*P*-value < 0.1, permutation test with 1,000 permutations, FDR correction). Similarly, only the SINE class shows enrichment of both HSC & B-cell and Blood & T-cell epigenomes among those with a higher contribution of TEs to the enhancer states (6_EnhG and 7_Enh). Thus, the patterns observed in Fig. 4a are not evenly distributed across TE classes.

Finally, we compared the average genome-wide expression level to the TE expression level, which recapitulates the results observed with epigenetic marks (Fig. 4c). The median ratio of TE-to-genome expression is 14% across all epigenomes, but the range is 2–45%, with blood, brain, and ESC epigenomes exhibiting a much higher TE expression level relative to the rest of the genome. All classes exhibit higher relative expression in blood and brain, while the ESC pattern is observed only for LTRs (Fig. 4d). Together, these results point to a potential biological role for specific TE subgroups in specific organs, which may be facilitated by tissue-specific expression of transcription factors whose binding sites are encoded by various phylogenetic lineages of TEs.

### Tissue-specific subfamily enrichment in epigenetic states

**Fig. 5:**
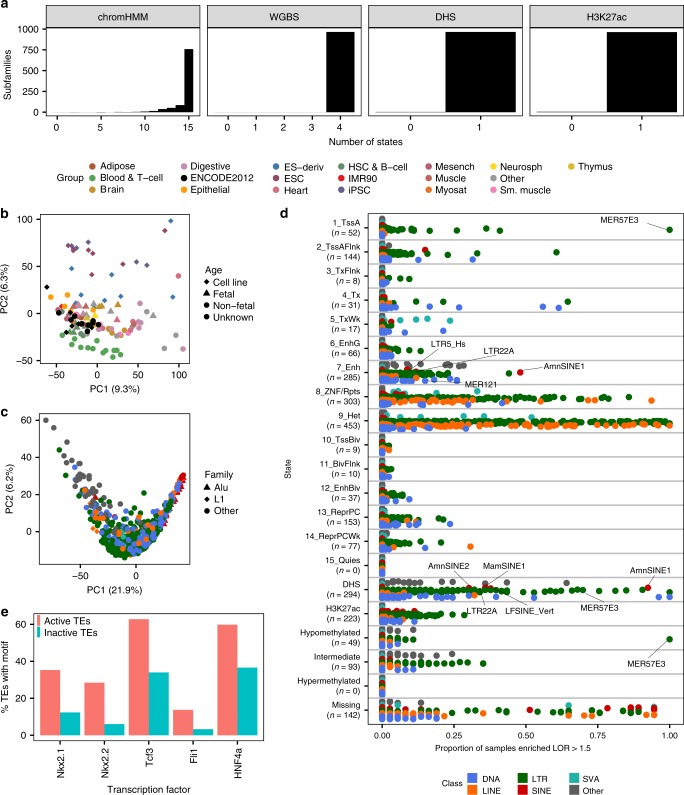
Tissue-specific enrichment of TE subfamilies in epigenetic states. **a** The total number of epigenetic states with which each TE subfamily is annotated across all epigenomes (see Methods; *n* = 968 TE subfamilies, *n* = 965 for methylation states). **b** PCA on Roadmap epigenomes (*n* = 127 epigenomes), using the LOR enrichment of each TE subfamily in each chromHMM state (13,716 subfamily-by-state combinations) as variables. Color is based on group (see legend above Fig. 5b), and shape is based on epigenome age (non-fetal and unknown age are not distinguished). **c** PCA on TE subfamilies (*n* = 937 subfamilies), using the LOR enrichment of the subfamily in each chromHMM state in each Roadmap epigenome (1,904 state-by-epigenome combinations) as variables. Color is based on class (see legend below Fig. 5d), and shape is based on TE family (only Alu and L1 are highlighted). **b**, **c** The amount of variation explained by each PC is listed in parentheses. **d** The proportion of epigenomes each TE subfamily is enriched LOR > 1.5 in the epigenetic state (chromHMM states 127 epigenomes, methylation states 37, DHS 53, H3K27ac 98). Each subfamily is represented by a circle and is colored by TE class (see Methods). The *n* below each state on the *y*-axis indicates the number of subfamilies enriched in the state in at least one epigenome. **e** The percentage of LTR22A elements that contain a binding motif for the transcription factor. Active TEs: elements annotated with the 7_Enh enhancer state in epigenomes where the subfamily is enriched in the state (*n* = 103 TEs); Inactive TEs: elements never in the state (*n* = 68 TEs). The top 5 most significant transcription factors as predicted by HOMER are shown (binomial FDR-corrected *P*-value < 0.0001 for each).

We next asked whether the tissue specificity we observed at the class level was present at the finer phylogenetic resolution of the TE subfamily. The vast majority of TE subfamilies overlap all 15 chromHMM states (759 subfamilies, 78%), all four methylation states (100% of subfamilies with CpGs), and DHS and H3K27ac peak summits (965 and 963 subfamilies, respectively) in at least one Roadmap epigenome (Fig. 5a; Supplementary Fig. 12a), and most subfamilies are in most states in ≥75% of epigenomes (Supplementary Fig. 12b). These numbers generally match expectation (ten iterations of shuffled TEs; Supplementary Fig. 12a), except that 92% of shuffled subfamilies overlap all 15 chromHMM states (*n* = 894, standard deviation = 6). Supplementary Fig. 12b suggests that this is due to more overlap with the small active and poised regulatory states, possibly due to exclusion of true TEs from developmentally important gene promoters.

To identify subfamilies exhibiting coordinated epigenetic profiles, we calculated the log odds ratio (LOR) enrichment of each TE subfamily in each epigenetic state in each epigenome compared to genomic background (Equation (1)). In total, there are 32,947 enrichments with LOR > 1.5 (approximately a three-fold enrichment over genomic background), 70% of which are in the 8_ZNF/Rpts and 9_Het states. Shuffling TE locations abrogates the vast majority of enrichments and enriched subfamilies (Supplementary Fig. 12c, d), confirming that many subfamily elements have coordinated epigenetic profiles.

Subfamily enrichment in epigenetic states can differentiate epigenome categories. Principal component analysis (PCA) performed on chromHMM state enrichments clearly separates Roadmap groups, including ESCs/iPSCs (induced pluripotent stem cells) and blood epigenomes along the second principal component (PC2) (Fig. 5b). Brain and digestive epigenomes and other organs form a large cluster in the center of the plot. This analysis closely recapitulates the results observed using genome-wide epigenetic profiles of the Enh and ReprPC states, which also separated these major epigenome groups^19^ . Interestingly, in our analysis, fetal epigenomes form a subcluster within the tissue-based clusters, suggesting that developmental stage influences the epigenetic profile of TE subfamilies. Similar results are observed for different epigenetic assays (Supplementary Fig. 13a–d) and epigenome classifications (Supplementary Fig. 13e–h), including that cancer cell lines cluster to one side of PC1. Subfamilies can also be distinguished by their epigenetic profiles (Fig. 5c; Supplementary Fig. 13i–k).

**Fig. 6:**
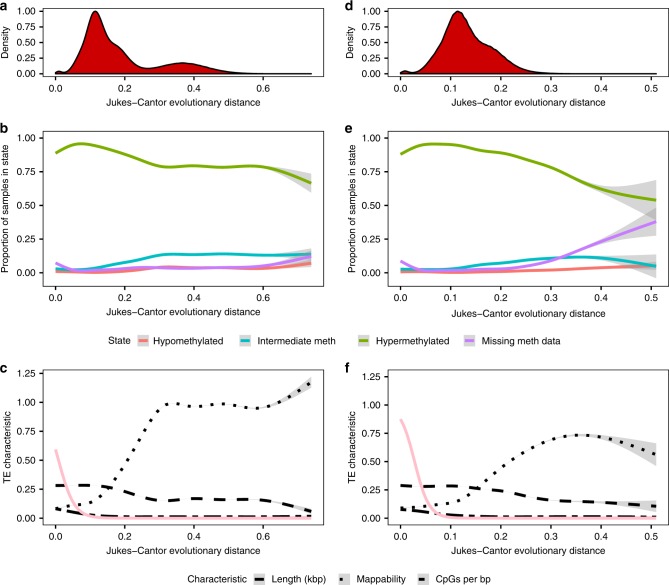
SINE and Alu methylation as a function of age. **a** Scaled density plot of Jukes-Cantor evolutionary distance (age) for SINE elements (*n* = 1,430,171 SINE elements with CpGs). **b** Generalized additive models of the proportion of epigenomes (total 37 epigenomes) in which individual SINE elements are annotated with each methylation state vs. age, smoothed using cubic regression splines. **c** Generalized additive models of SINE element characteristics vs. age, smoothed using cubic regression splines. The pink line represents a logistic regression model of the likelihood a SINE element overlaps a CpG island vs. age. **d** Scaled density plot of Jukes-Cantor evolutionary distance (age) for Alu elements (*n* = 1,105,597 Alu elements with CpGs). **e** Generalized additive models of the proportion of epigenomes (total 37 epigenomes) in which individual Alu elements are annotated with in each methylation state vs. age, smoothed using cubic regression splines. **f** Generalized additive models of Alu element characteristics vs. age, smoothed using cubic regression splines. The pink line represents a logistic regression model of the likelihood an Alu element overlaps a CpG island vs. age.

To identify TE subfamilies that may perform tissue-specific coordinated regulatory functions, we next identified subfamilies that are enriched LOR > 1.5 more often than expected in an epigenome category in an active state (active regulatory or transcribed chromHMM state, hypo- or intermediately methylated states, or DHS or H3K27ac peak overlap; FDR-corrected *P*-value < 0.05, permutation test; Fig. 5d; Supplementary Data 4; Supplementary Discussion). As in ref. ^1^, we observe enhancer state (7_Enh) enrichment of LTR77 in blood and UCON29 in brain, as well as numerous subfamilies with ESC/iPSC-specific enrichment. However, the diversity of anatomical sites sampled by the Roadmap Project also allows us to identify subfamilies with previously uncharacterized tissue-specific activity. For example, LTR22A, a relatively young ERVK subfamily, is preferentially enriched in the 7_Enh enhancer state and in DHS and H3K27ac peak overlap in digestive system epigenomes. Although 69–74% of LTR22A elements in those states when the subfamily is enriched are intergenic, many overlap lincRNAs (long intergenic non-coding RNAs) and antisense RNAs, and others are within genes with digestive system-specific expression, including *DDC* and *GPR160*. Individual elements annotated with 7_Enh in epigenomes where the subfamily is enriched are also more likely to encode binding sites for HNF4a, a transcription factor implicated in intestinal development, compared to elements never in the state (60% vs. 37% of elements, binomial test, Benjamini-corrected *P*-value = 0.0002; HOMER; Fig. 5e)^28^.

Despite comprising 52% of subfamilies, LTR subfamilies represent ≥70% of those enriched at least once in many of the active states, and the number of enrichments in these states decreases with subfamily age (median Jukes-Cantor evolutionary distance to the subfamily RepBase consensus) and loss of promoters (*P*-value < 0.001, Spearman correlation; Supplementary Fig. 14a, b). In contrast, the oldest SINE subfamilies, AmnSINE1, AmnSINE2, MamSINE1, and LFSINE_Vert, together account for 90% of the 279 enrichments of SINE subfamilies in active states. All four subfamilies are enriched in DHS peak overlap in >30% of epigenomes. AmnSINE1 is preferentially enriched in 7_Enh in epigenomes of ectodermal origin, particularly epithelial epigenomes, while MamSINE1 is preferentially enriched in epithelial epigenomes for DHS peak overlap. Interestingly, AmnSINE1 is preferentially enriched in fetal epigenomes for H3K27ac peak overlap. LFSINE and AmnSINE1 have been previously implicated as enhancers in brain formation^1,29,30^, and AmnSINE1 elements in 7_Enh when the subfamily is enriched are enriched for Gene Ontology (GO) Biological Processes involved in differentiation, development, and morphogenesis (Supplementary Fig. 14c)^31^.

The DNA subfamily MER121 is enriched in the 7_Enh state in a group of primary cultures of mesodermal origin, including mesenchymal stem cells, adipocytes, chondrocytes, fibroblasts, osteoblasts, myoblasts, and myosatellites. 65% of the elements in the state when the subfamily is enriched are intergenic, and they are associated with several GO Biological Processes involved in embryonic morphogenesis and development, driven by their proximity to genes such as *SOX9*, *BMP4*, and *FGF10* (GREAT; Supplementary Fig. 14d).

We also observe several TE subfamilies with fetal-specific DHS enrichment, including MER131 (Other class). MER131 elements annotated with the state in epigenomes where the subfamily is enriched are enriched in several GO Biological Processes, including embryonic morphogenesis (Supplementary Fig. 14e). MER41C and MER39B are enriched in several active states in placenta or trophoblast epigenomes, and several LTR subfamilies, primarily from the ERV1 family, are preferentially enriched in active regulatory states in cancer cell lines, including LTR7C.

In addition to tissue-specific patterns, a few subfamilies are enriched in an active state in all epigenomes. MER57E3, an ERV1 family LTR subfamily, is enriched in all epigenomes in the 1_TssA promoter state and hypomethylated CpGs and in the DHS state in 70% of epigenomes. On average, 28% of MER57E3 elements are in the 1_TssA state in each epigenome. Of its 238 members, 156 are on chromosome 19 (66%), and 18 overlap promoters, including KRAB-ZNF (Krüppel-associated box-zinc finger) genes, which play important roles in repressing TEs^32^. Interestingly, in most cases, the TE is in the same relative position slightly downstream of the core promoter. We therefore speculate that MER57E3 elements were propagated along with KRAB-ZNF genes during primate evolution, rather than propagating independently.

By calculating subfamily enrichment using the 50-state chromHMM models generated for seven reference epigenomes (Supplementary Fig. 15), we further explored the coordinated regulatory role of TEs. MER121 is enriched in E017 (IMR90) in the 7_Enh enhancer state, as well as five enhancer-like states (E26, E30-32, E37) from the 50-state model. Although three of the states are characterized by numerous histone modifications, E30 is characterized primarily by H3K4me2, DNase, and H2BK5ac, while E37 is characterized by H3K4me1, H4K5ac, and H3K18ac. Further research could determine whether these epigenetic differences correspond to different roles for subsets of MER121 elements.

Finally, we observe tissue-specific enrichment in active epigenetic states in very young subfamilies, suggesting that they are not universally repressed (Supplementary Discussion). This includes LTR5_Hs, the LTR of the HERVK (HML-2) group of endogenous retroviruses (ERVs), in pluripotent epigenomes. HERVK (HML-2) is the most recent ERV to enter the human genome and the only one still theoretically capable of retrotransposition^33^. We also observe subfamilies representing >1% of an active epigenetic state in the genome despite a LOR < 1.5 (Supplementary Fig. 16a–d; Supplementary Discussion), underscoring the contribution of large Alu, MIR, and L1 subfamilies to the epigenome.

### TE epigenetic profiles as a function of age

Having observed extensive variation in the epigenetic profile of TEs, we next identified factors that correlate with the epigenetic state of individual TEs. As expected, the chromosome on which a TE resides impacts its epigenetic profile (Supplementary Fig. 17a–e), as does overlap with genes (Supplementary Fig. 18a; Supplementary Discussion). Indeed, 18% of TEs ever in the 1_TssA state overlap RefSeq promoters (vs. 2% of all TEs), and 89–90% of TEs ever in the 4_Tx and 6_EnhG states are within RefSeq introns (vs. 45% of all TEs). Interestingly, while most TEs in the 4_Tx state appear to be intronic passengers (Supplementary Fig. 18b), more TEs overlapping DHS peaks or in the 7_Enh state in only a few epigenomes are intergenic and thus more likely to be independent regulatory elements. Active LTR elements are also more likely to be intergenic than are other classes.

The frequency with which a TE is hypo- or intermediately methylated is slightly positively correlated with evolutionary age, while the opposite is true for hypermethylation (Spearman correlation; Supplementary Fig. 19a–d). This pattern is most striking for SINE elements (Fig. 6a, b). Interestingly, SINE TEs decrease not just in length but in CpG density with age, which would not be expected if CpGs were evenly distributed across the TE (Fig. 6c). Indeed, older SINE TEs are significantly less likely to overlap CpG islands (logistic regression, Jukes-Cantor evolutionary distance predictor coefficient *P*-value < 0.001), and the median age of SINE elements that overlap CpG islands is much lower than those that do not (0.04 vs. 0.17). Taken together, these results indicate that SINE elements with CpG islands are subject to greater levels of repressive DNA methylation, but that the CpG islands are eliminated from the TEs as they age.

To confirm that the trends observed for all SINE elements are not the result of bimodal age distribution between the older SINE families (MIR, Deu, tRNA, and SINE, median Jukes-Cantor evolutionary distance 0.37 ± interquartile range (IQR) 0.10; Fig. 6a) and the younger Alu subfamilies (median distance 0.13 ± IQR 0.07; Fig. 6d), we repeated the analysis with only Alu elements (66% of SINE TEs), which recapitulated the results (Fig. 6e, f; Supplementary Table 3).

### Evolutionary conservation of TE regulatory signatures

Finally, we investigated whether the regulatory signatures we observed in human TEs are evolutionarily conserved by comparing them to orthologous TEs in the mouse genome. Although human and mouse diverged approximately 90 million years ago^34^, 6% of hg19 TEs have an identifiable corresponding region in the mm10 genome (*n* = 269,096) and overlap an mm10 TE from the same subfamily (*n* = 269,801), which we consider orthologous pairs (Supplementary Fig. 20a).

**Fig. 7:**
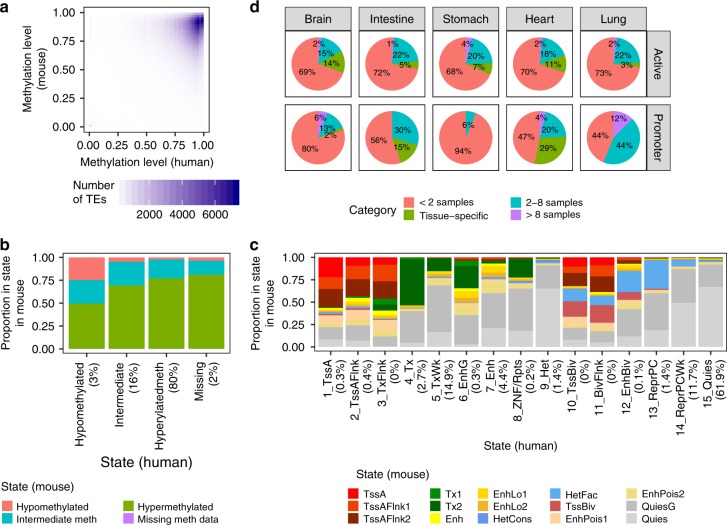
Conserved epigenetic profiles of orthologous TEs in human and mouse. **a** 2D density plot of the methylation level of orthologous TEs (*n* = 140,185 TEs) in seven anatomy-matched human-mouse epigenome pairs. TE pairs missing methylation in either epigenome or lacking CpGs are excluded. **b** Methylation state of orthologous TEs in human and mouse across the seven epigenome pairs. Percentages to the right of each *x*-axis state indicate the proportion of TE ortholog instances across the epigenome pairs that are annotated with that state. **c** chromHMM state of orthologous TEs (*n* = 296,457 TEs) in human and mouse across twelve anatomy-matched epigenome pairs. Percentages to the right of each *x*-axis state indicate the proportion of TE ortholog instances across the epigenome pairs that are annotated with that state. **d** Tissue-specificity profiles in mouse for orthologous TEs that exhibit tissue specificity in that tissue in human. TEs are considered tissue-specific if they are annotated with a state in two epigenomes from the same tissue but fewer than 5 epigenomes overall. Active: any active regulatory state (Brain *n* = 1,812 TEs, Intestine *n* = 1,691, Stomach *n* = 205, Heart *n* = 3,081, Lung *n* = 653); Promoter: 1_TssA/TssA (Brain *n* = 54 TEs, Intestine *n* = 27, Stomach *n* = 16, Heart *n* = 49, Lung *n* = 16). TEs in the 2–8 epigenomes category are not specific to the tissue.

We profiled the methylation level and chromHMM state of the orthologous TEs in twelve samples interrogated by the mouseENCODE project^21–23^ that anatomically matched human Roadmap epigenomes (Supplementary Table 4). Although only a small fraction of orthologous TEs is hypomethylated in either human or mouse, those hypomethylated in one species are significantly more likely to be hypomethylated in the other in the corresponding tissue than expected by random chance (*P*-value < 0.001, Chi-squared test for seven human-mouse epigenome pairs with WGBS data; Cramer’s V 0.10 to 0.14; Fig. 7a, b). This result holds true when all methylation states are considered (Cramer’s V 0.16 to 0.23). Thus, the DNA methylation level of orthologous TEs is more conserved than expected between human and mouse.

The histone modification profile of orthologous TEs is also conserved across species. Although the human and mouse epigenomes were annotated with different chromHMM models, there is a non-random distribution of orthologous TEs in each human/mouse chromHMM state combination (*P*-value < 0.001, Chi-squared test for twelve epigenome pairs; Cramer’s V 0.22 to 0.26). Figure 7c demonstrates a clear enrichment of the promoter state (TssA) in mouse for TE orthologs annotated with the promoter state (1_TssA) in human, as well as an enrichment in multiple active regulatory mouse chromHMM states (mouse: TssA, TssAFlnk1, TssAFlnk2, Enh, EnhLo1, and EnhLo2) for orthologs in human active regulatory states (human: 1_TssA, 2_TssAFlnk, 3_TxFlnk, 6_EnhG, 7_Enh). The epigenetic profile of shared TE subfamilies (578 of 968 human subfamilies; Supplementary Fig. 20b) is also conserved across species (Supplementary Fig. 20c–e).

We next identified TEs that exhibit conserved tissue-specific epigenetic profiles (Fig. 7d). Of the TEs with tissue-specific promoter state (1_TssA) annotation in humans (*n* = 162), 0–29% also exhibit tissue-specific promoter state (TssA) annotation in mouse in the same tissue. When considering all active regulatory states (*n* = 7,442), 3–14% of tissue-specific TEs exhibit the same profile in mouse. All TEs with conserved tissue-specific promoter annotations (*n* = 19) are located within or near human RefSeq genes, and in many cases, their conserved epigenetic profile likely stems from overlap with the promoter region of a gene whose tissue-specific expression profile is conserved between the species. For example, two conserved MIR elements (chr12:57443327–57443493 and chr19:39302694-39302815) overlap *MYO1A* and *LGALS4* or their promoters, which have high intestinal expression. However, 54 of the TEs with conserved active regulatory annotations are >50 kb from the nearest RefSeq gene, including 28 that are >100 kb. Four such elements are displayed in Supplementary Fig. 21, including three brain-specific enhancers and an intestine-specific element that is an enhancer in human and a promoter in mouse. These include a UCON29 element, a subfamily that has been shown to have brain-specific enhancer activity in this paper and others^1^.

## Discussion

In this study, we use multidimensional epigenomic data produced by the Roadmap Epigenomics Project to quantify the substantial contribution of TEs to active epigenetic states in the human genome across >100 diverse tissues and developmental timepoints. As suggested by Britten and Davidson^35^, TEs represent a unique opportunity for the host to efficiently bring distant regions under the control of the same gene regulatory network. In this model, the ancestral TE disseminates a battery of transcription factor binding sites throughout the genome, and those that insert in favorable locations for the host are conserved, while others are neutralized through mutation. Thus, TEs allow the host to circumvent the independent evolution of multiple adjacent binding sites^7^. Conversely, some of the TE-derived regulatory activity we observe may have no impact on the host, but rather represents regulatory elements that have not yet been eliminated. TEs are also repressed by a variety of specialized mechanisms, including KRAB-ZNF genes^32^, which may restrict TE activity in a manner that does not affect non-TE-derived regulatory elements.

By comparing the epigenetic profile of TEs to shuffled TE locations, we confirm that TEs are slightly depleted from active epigenetic states, consistent with the reported overlap between TEs and transcription factor binding sites^6^. However, this also confirms that TEs are not universally repressed, a relatively new mode of thinking. We also demonstrate that the specific TEs in active regulatory states are not random and represent coordinated subfamily activity (Supplementary Fig. 12c–d).

In our results, TEs belonging to the Other class disproportionately contribute active regulatory states to the genome (Fig. 1d). These TEs are much older (Supplementary Fig. 3d), affording more opportunity for the host to co-opt their regulatory sequences or neutralize them to the point where they are no longer annotated as TEs. The oldest SINE subfamilies also have higher regulatory signatures than the younger Alu subfamilies, which are repressed via DNA methylation. In contrast, younger LTR subfamilies are more often enriched in active regulatory states (Fig. 5d; Supplementary Fig. 14b). ERVs comprise an entire viral genome on insertion and retain intact regulatory elements even after LTR recombination eliminates the intervening exons^36^. They are also more likely to be intergenic, potentially reducing their impact on existing gene networks and increasing tolerance of their activity by the host. Furthermore, LTRs undergo a switch in repression mechanism from DNA methylation to H3K9 histone methylation with evolutionary age, which could influence their activity profile^37^. Thus, diverse origins and structures influence the epigenetic profile of TE classes and subfamilies.

Neuronal cells have been previously reported to have a higher level of TE retrotransposition^38^, and we demonstrate a higher relative expression level of TEs in brain than in other epigenomes. However, this is not matched by increased TE biochemical activity. In contrast, we observe a striking enrichment of TE expression and epigenetic signatures of regulatory activity within TEs in blood. Blood is relatively depleted in the 5_TxWk state (Supplementary Fig. 1), such that TEs with a constant level of 5_TxWk annotation can represent a higher proportion of the state in those cell types. However, this is not true for the 6_EnhG and 7_Enh states, suggesting that TEs are preferentially active as enhancers in those epigenomes.

An outstanding question in the field has been whether TE-derived regulatory elements are conserved or species-specific. In a minority of cases, we observe that intergenic orthologs with a tissue-specific activity profile in humans retain the same profile in mouse, suggesting that regulatory activity that developed before the human-mouse split was maintained in both species independent of overlapping genes. However, the mouse samples used in our comparison are significantly younger than the human samples, and we may observe greater conservation using age-matched tissues. Other potential confounders that could artificially reduce the concordance between species include differences in tissue collection, processing, and storage protocols, as well as epigenetic assays, between laboratories and consortia; sequencing batch effects; the use of multiple chromHMM models; and a limited ability to computationally identify orthologous TEs. Recent evidence has also suggested that TEs can be involved in turnover events, wherein TEs encoding a transcription factor binding site can step in when a nearby binding site is lost, preserving the function of the region using novel or lowly conserved sequence^39^.

As mentioned above, Cao et al.^20^ used a machine learning model trained on ENCODE histone modification data to demonstrate that 35% of TEs have signatures of *cis*-regulatory elements. Although this is moderately lower than our estimate, they reveal via saturation analysis that more TEs may perform regulatory functions in additional cell types. They also confirmed that TE enhancer activity is highly tissue-specific and can be used to distinguish cell types, and they profiled the tissue-specific enrichment of TE families as enhancer- or promoter-like elements in Roadmap tissues. Interestingly, they found that TEs with ESC/iPSC enhancer activity had highly conserved epigenetic profiles in primates, but less so in less related species, and they uncovered widespread 3D interactions between MIR and L2 elements that modulate their enhancer activity.

An additional study that identified TE-derived promoters and enhancers using FANTOM5 CAGE-seq (cap analysis gene expression) data^18^ determined that 45% of enhancers and 5% of promoters overlap TEs, lower than random expectation, with more tissue-specific enhancers overlapping TEs, particularly those specific to blood and testis. Older TE families had more members overlapping enhancers, as did ERVs. Additionally, they found that although enhancers are biased toward overlap with mammal-specific and ancient TEs, enhancers that overlap younger TEs are more likely to be active in only a single tissue. Villar et al.^40^ also identified an enrichment of younger repeat families in recently evolved enhancers, although ancient ERV families are more likely to be exapted as recently evolved promoters.

An important caveat to our results is the exclusive use of uniquely mapped, short (36 bp) reads to generate chromHMM state assignments and peak calls. TEs present a mappability challenge due to their repetitive nature, particularly for short reads and young TE subfamilies that have not accumulated sequence changes over time, such as the SVA subfamilies, young Alu and L1 subfamilies, and some ERVs. Although we capture enrichment of very young subfamilies with low mappability in active regulatory states, it is possible that using multimapped reads assigned at the subfamily level would reveal additional TE activity and that our results represent a lower bound. In particular, many SVA and SINE elements have low mappability due to a combination of age, length, and prolific expansion (Supplementary Fig. 3d), and these classes may be disproportionately affected by mappability. However, mappability is not correlated with the number of epigenomes a TE is annotated with the 15_Quies state (Spearman correlation, rho = 0.01, *P*-value < 0.001), and the TEs in each chromHMM state exhibit histone modification, chromatin accessibility, and DNA methylation profiles characteristic of that epigenetic state (Supplementary Fig. 22a).

In cancer, the genome undergoes global DNA hypomethylation and exhibits dysregulated chromatin^41^. The enormous number of TEs with narrowly restricted regulatory profiles suggests that they are subject to tight epigenetic control and could be rapidly de-repressed during malignant transformation. In line with this hypothesis, we observe greater contribution of TEs to active regulatory states in cancer cell lines. Aberrantly activated TEs can alter the expression of nearby genes, serve as alternative promoters that form chimeric or immunorestricted transcripts, or even drive oncogene expression^36^. However, epigenetic therapies may also potentiate cancer immunotherapy through the activation of TEs, which increase tumor immunogenicity by forming double-stranded RNA and immunogenic proteins^42–46^. Resources such as this study could inform predictions of which TEs are activated in cancer and in response to epigenetic therapies, including in normal tissues to prevent potential off-target effects.

In conclusion, this study represents an important synthesis of epigenetic data in the context of TEs that could serve as a resource for investigations of this underexplored aspect of the human genome in healthy and diseased states.

## Methods

### Data download

Data from the Roadmap Epigenomics Project were downloaded from the data portal (http://egg2.wustl.edu/roadmap/; see Data Availability). For this study, all consolidated epigenomes were included (*n* = 127), although not all epigenetic marks were profiled for each epigenome. chromHMM state assignments for the 15-state model are presented with the same colors in this paper as in the original Roadmap paper (https://egg2.wustl.edu/roadmap/web_portal/chr_state_learning.html#core_15state), with the exception of 15_Quies (light gray instead of white). Colors for the 18-state chromHMM model are from the Roadmap data portal (https://egg2.wustl.edu/roadmap/web_portal/chr_state_learning.html#exp_18state). Supplementary Fig. 4 of ref. ^19^ provides the 18-state chromHMM model state for which each 50-state model state is most enriched, which was used to assign colors and broader state categories to each of the 50-state model states. The figure was also used to identify histone modifications that characterize each state. Epigenome Group assignments also use the same colors as in the Roadmap paper. TE class colors are those used in the RepeatBrowser track of the WashU Epigenome Browser, except for SVA (Other on the track).

mm10 chromHMM assignments and CpG methylation levels were downloaded from the ENCODE data portal (https://www.encodeproject.org/) (see Supplementary Table 4 for accessions). Human epigenomes from the Roadmap Epigenomics Project and mouse epigenomes from ENCODE were paired based on anatomy. Mouse chromHMM state assignments were generated with a different 15-state model incorporating 8 epigenetic marks (the five core histone modifications used in the hg19 state assignments plus H3K27ac, H3K9ac, and H3K4me2)^21–23^. Although the promoter and active regulatory states are not identical between the hg19 and mm10 chromHMM state assignments, we confirmed that characteristic histone modifications are similar for each (e.g., 1_TssA and TssA, 2_TssAFlnk and TssAFlnk1/TssAFlnk2, and 7_Enh and Enh/EnhLo1/EnhLo2). Colors were assigned to mm10 chromHMM states based on similarity to hg19 chromHMM states.

hg19 chromosome sizes; RepeatMasker annotations for hg19 and mm10; hg19 RefSeq genic feature locations; hg19 CpG island locations (cpgIslandExtUnmasked.txt); the hg19 36 bp mappability track (wgEncodeCrgMapabilityAlign36mer.bw); and GENCODE v19 comprehensive genes were downloaded from the UCSC Genome Browser.

### Epigenome metadata

In addition to the Group, Anatomy, and Type metadata assigned to each epigenome by the Roadmap Project, we assigned three additional categories, Cancer, Age, and Germ layer (Supplementary Data 3). Cancer: Cancer and cancer cell lines were designated “yes”; all other epigenomes designated “no”. Age: Epigenomes were split into age categories based on the age of the donor or presence of the words “fetal” or “adult” in the epigenome name. ENCODE cell lines were assigned to “Unknown” or “Cell line” based on whether they were listed as “primary cells” or “cell line” in the epigenome name or in ref. ^21^. Germ layer: Epigenomes were split into germ layer of origin, including endoderm, ectoderm, and mesoderm, “mixed” (organs with multiple germ layer origins), “pluripotent” (ESC/iPSC cells plus mesendoderm cells), and “other” (placenta/trophoblast).

### Feature pre-processing

All analyses were restricted to chromosomes 1–22, X, Y, and M for hg19 and chromosomes 1–19, X, Y, and M for mm10. 31,580 TEs are on chromosome Y (0.7%) and are missing from six Roadmap epigenomes without chrY annotations.

RepeatMasker-annotated repeats were restricted to classes LTR, DNA, SINE, LINE, Unknown, Unknown?, DNA?, LINE?, SINE?, LTR?, RC, RC?, and Other. For hg19 by-class analyses, RepeatMasker class Other was renamed “SVA”, as it includes only SVA subfamilies. Classes Unknown, Unknown?, DNA?, LINE?, SINE?, LTR?, and RC were combined into a single category (“Other” class).

Promoters were generated by extending the region 2000 bp upstream of RefSeq transcription start sites to 500 bp downstream using *bedtools slop*^47^. RefSeq promoters and exons were collapsed by coordinate and strand to identify unique features. Intergenic regions were generated by taking the complement of RefSeq genes using *bedtools complement*. RefSeq features were split into protein-coding and non-coding features based on accession (NM vs. NR).

The Jukes-Cantor evolutionary distance for each TE was calculated from the substitution rate compared to the RepBase consensus sequence. A mappability score for each TE was calculated in^6^ using the 36 bp mappability track from the UCSC Genome Browser, as all ChIP-seq reads were trimmed to 36 bp for use in chromHMM state assignment.

### Intersection with Roadmap data

Features were intersected with epigenetic data or other features using *bedtools intersect* without regard to strand, except where noted (Supplementary Fig. 2a). Features were considered overlapping if they overlapped by ≥1 bp. CpGs where one base did not overlap the feature were included in the total as half a CpG. As in the Roadmap Epigenomics Project, CpGs whose read coverage was ≤3 reads were considered missing values.

The proportion of the entire genome, chromosome, TEs, TE classes, TE subfamilies, and RefSeq features in each state or overlapping another feature was calculated as the proportion of all unique bases or CpGs within the feature that were (1) annotated with the chromHMM state, (2) at that methylation level (WGBS, divided into four methylation states), (3) overlapping a DHS or H3K27ac peak (not limited to peak summits), or (4) overlapping the second feature. The exceptions are chromosome and subfamily enrichment analyses, where the number of unique DHS/H3K27ac peak summits overlapping the feature were counted (Supplementary Fig. 17c, d; Fig. 6b–d). The average expression level over the entire genome, all TEs, and each TE class was calculated as the average read coverage over the feature.

The contribution of TEs to the DHS and H3K27ac states (Figs. 1c and  4a) is roughly equivalent whether total bases overlapping peaks or number of peaks is considered. TEs overlap 27% of DHS peak summits across all epigenomes, with a range of 19% vs. 35% per epigenome, and 26% of H3K27ac peaks, with a range of 17% vs. 38% per epigenome.

For Roadmap analyses, an individual TE or promoter was considered annotated with a state if it overlapped the center of a 200 bp chromHMM state annotation or the summit of a DHS or H3K27ac peak. Additionally, TEs located between 200 bp chromHMM window centers (18% of TEs) were assigned the state with the largest overlap with the TE, such that every TE was assigned to at least one chromHMM state. The same rules were applied for the 15-state, 18-state, and 50-state chromHMM models. Because there is variation in the number and average width of chromHMM blocks and DHS/H3K27ac peaks by epigenome, a TE is more likely to be annotated with a state in some epigenomes because there are more peak summits available to overlap (Supplementary Fig. 22b; Supplementary Discussion). A TE or promoter was considered in a methylation state if the mean methylation level (calculated over all CpGs overlapping the TE/promoter with >3 reads) fell within the range of that state. Features not overlapping CpGs were not considered for methylation analyses. The expression level of individual TEs and exons was calculated as the average read coverage over the feature, normalized by the normalization factors provided by Roadmap. RNA-seq coverage on chromosome Y in epigenomes lacking that chromosome (*n* = 6) was not included in the analyses.

Two alternative methods of annotating individual TEs with a chromHMM state were explored: (1) counting only TEs overlapping the center of a chromHMM annotation block and (2) counting only TEs overlapping the center of a 200 bp chromHMM annotation window (Supplementary Fig. 4a, b). The number of TEs annotated with the state per epigenome and in any Roadmap epigenome were calculated in a manner identical to that for the standard annotation rules.

Across all instances of an individual TE overlapping a DHS peak summit in an epigenome, 93% overlap only one peak, but they can overlap up to 17 DHS peaks in a single epigenome. Similarly, 97% of TEs overlapping H3K27ac peak summits overlap only one peak, but they can overlap up to 14 H3K27ac peaks in a single epigenome.

The chromHMM state of mm10 TEs was any state the TE overlapped by 1 bp or more. Methylation states were calculated as for hg19 TEs.

### Shuffled TEs

The genomic positions of all TEs were shuffled using *bedtools shuffle* with default arguments, excluding chrM, contigs, and genome gaps. Ten iterations of shuffled TEs were generated. Individual shuffled TEs were annotated with epigenetic states using the same rules as for real TEs. Epigenetic dynamics, including the total number of states per TE across all epigenomes and aggregate profiles of epigenetic states across epigenomes (Supplementary Fig. 9), and subfamily potential and enrichment (Supplementary Fig. 12) were calculated in an identical manner as for real TEs. For subfamily enrichment in methylation states, the number of CpGs per subfamily was recalculated using the shuffled subfamily.

### Permutation tests

To identify epigenome categories (i.e., Group: ESC) with enriched representation among the epigenomes with a higher or lower proportion of the state within TEs or a TE class than the cross-epigenome total, we permuted the category labels 1,000 times and compared the true representation to this distribution. Tests were performed simultaneously across all categories (e.g., Group, Germ layer; Supplementary Data 2), and epigenomes with proportions higher and lower than the cross-epigenome total were tested separately. FDR correction was performed only on epigenome categories with any epigenome higher or lower than the cross-epigenome total.

### Subfamily enrichment

The enrichment of a TE subfamily *i* in a state *j* in an epigenome *k* was calculated as the log odds ratio (LOR) as in ref. ^6^, as follows:

1 $${\mathrm{log}}_2\left( {1{\mathrm{e}}^{ - 20} + \frac{{\frac{{\left( {ijk,{\mathrm{bp}},{\mathrm{peak}}\,{\mathrm{summits}},\,{\mathrm{or}}\,{\mathrm{CpGs}}\,{\mathrm{in}}\,{\mathrm{subfamily}}\,{\mathrm{in}}\,{\mathrm{state}}\,{\mathrm{in}}\,{\mathrm{epigenome}}} \right)}}{{\left( {ik,{\mathrm{bp}}\,{\mathrm{or}}\,{\mathrm{CpGs}}\,{\mathrm{in}}\,{\mathrm{subfamily}}\,{\mathrm{in}}\,{\mathrm{epigenome}}} \right)}}}}{{\frac{{\left( {jk,{\mathrm{bp}},\,{\mathrm{peak}}\,{\mathrm{summits}},\,{\mathrm{or}}\,{\mathrm{CpGs}}\,{\mathrm{in}}\,{\mathrm{state}}\,{\mathrm{in}}\,{\mathrm{epigenome}}} \right)}}{{\left( {k,{\mathrm{bp}}\,{\mathrm{or}}\,{\mathrm{CpGs}}\,{\mathrm{in}}\,{\mathrm{epigenome}}} \right)}}}}} \right)$$

A pseudocount of 1e^−20^ was included to avoid –Inf values. For chromHMM states, *ijk* and *jk* were calculated as the total bp in the state overlapping the subfamily or in the entire genome; for WGBS, total CpGs; and for DHS and H3K27ac, total peak summits. *ik* and *k* refer to the lengths of the subfamily and genome. The LOR enrichment of each subfamily in the 50-state chromHMM model states was calculated using the same rules and thresholds as for the 15-state model.

For subfamily potential analyses, a subfamily was considered annotated with a state if *ijk* was greater than zero (Fig. 5a).

PCA was performed using the prcomp() function on variables with variance >0 between the epigenomes/subfamilies. Only subfamilies with >30 members (>30 members with CpGs for WGBS) were included. Matrices were scaled and centered before PCA was performed.

Only subfamilies with >30 total members and >10 members in the state in the epigenome were considered enriched (LOR > 1.5). With these thresholds, 31 subfamilies were excluded from chromHMM/DHS/H3K27ac enrichment analysis (plus 2 additional subfamilies in epigenomes where chrY was absent). For WGBS analyses, only members overlapping CpGs were considered, which excluded 23 additional subfamilies. Three TE subfamilies (Charlie1b_Mars, CheshMITE, and HAL1N1_MD) overlap no CpG. 34,892 enrichments were excluded by the second threshold, 10,757 of which were 8_ZNF/Rpts.

To identify subfamilies preferentially enriched in an epigenetic state in an epigenome category (e.g., Group: ESC), we performed permutation tests as above (Supplementary Data 4), testing whether epigenome categories were unusually represented among all enriched epigenomes for that state/subfamily combination. FDR multiple hypothesis correction was performed only on subfamily-by-state-by-category combinations with at least one epigenome with enrichment LOR > 1.5 and only on active states, and only categories with a corrected *P*-value < 0.05 were considered.

For candidate subfamilies, GREAT analysis was performed on all TEs in the state in epigenomes where the subfamily was enriched in the state. The default basal plus extension model (5 kb upstream, 1 kb downstream, 1,000 kb distal) was used, and only GO Biological Processes were considered. Terms were considered significant if the FDR-corrected *P*-value < 0.05 for both binomial and hypergeometric tests with a minimum region-based fold enrichment of 2. The default genomic background was used.

HOMER (findMotifsGenome.pl) was performed on candidate subfamilies to identify enriched known transcription factor motifs. TEs in the state in epigenomes where the subfamily was enriched in the state were compared to TEs from that subfamily that are never in the state in any Roadmap epigenome as background, with flags *-size given –nomotif*.

### Epigenetic profile modeling

To test the relative contribution of class and CpG density to the mechanism of TE repression, quasi-Poisson generalized linear regression models were constructed for the number of epigenomes a TE is in the 9_Het or hypermethylated states (function glm(), quasipoisson family; Supplementary Table 2). Class (LTR vs. SINE) and CpG density (CpGs per kbp) were included as predictors. ANOVA (analysis of variance) with F tests for significance was performed to test differences in performance between models.

Generalized additive models of TE length, mappability, CpG density, and the number of epigenomes a SINE or Alu element is in each methylation state were constructed using Jukes-Cantor evolutionary distance as the predictor (function gam(); Supplementary Table 3). Logistic regression models of the likelihood a SINE or Alu element overlaps a CpG island were constructed using Jukes-Cantor evolutionary distance (JC) as the predictor (function glm(), binomial family). All SINE elements with CpGs (*n* = 1,430,171) or Alu elements with CpGs (*n* = 1,105,597) were included in the model.

### Orthologous TEs

The mm10 genome has 3,663,513 TEs and 1,134 TE subfamilies. Orthologous TEs were identified by converting hg19 TE coordinates to mm10 using liftOver (*−minMatch* = *0.1*), then identifying overlapping hg19 -to-mm10 and mm10 TEs with identical subfamily names using *bedtools intersect*. 29% of hg19 TEs (*n* = 1,265,775) lift over to mm10. See the Supplementary Discussion for more detail on overlap between mm10 and hg19 TEs.

Chi-squared test effect size was calculated using Cramer’s V (from the R package rcompanion).

### Epigenetic profile of TEs in chromHMM states

For each chromHMM state, for all instances of a TE in that state in an epigenome, the average histone modification ChIP-seq/DHS signal fold enrichment ratio over input for several epigenetic marks was calculated in 50 bp bins over a 10 kb region centered on the TE (excluding 26 TEs that would extend beyond the end of the chromosome). The epigenetic marks include the five core histone modifications used to generate chromHMM (H3K4me1, H3K4me3, H3K9me3, H3K27me3, and H3K36me3), H3K9ac, H3K27ac, and DHS. The average methylation over the same window was calculated for epigenomes with WGBS data.

### Reporting summary

Further information on research design is available in the Nature Research Reporting Summary linked to this article.

## Supplementary information

Supplementary Information

Description of Additional Supplementary Files

Supplementary Data 1

Supplementary Data 2

Supplementary Data 3

Supplementary Data 4

Reporting Summary

## Data Availability

All relevant data supporting the key findings of this study are available within the article and its Supplementary Information files or from the corresponding authors upon reasonable request. All datasets used in this manuscript were made publicly available as part of previous publications. Data from the Roadmap Epigenomics Project were downloaded from the data portal, including: epigenome metadata [http://egg2.wustl.edu/roadmap/web_portal/meta.html]; chromHMM state assignments using the 15-state model, 127 epigenomes [http://egg2.wustl.edu/roadmap/data/byFileType/chromhmmSegmentations/ChmmModels/coreMarks/jointModel/final/all.mnemonics.bedFiles.tgz]; chromHMM state assignments using the 18-state model, 98 epigenomes [http://egg2.wustl.edu/roadmap/data/byFileType/chromhmmSegmentations/ChmmModels/core_K27ac/jointModel/final/all.mnemonics.bedFiles.tgz]; chromHMM state assignments using separate 50-state models, 7 epigenomes [http://egg2.wustl.edu/roadmap/data/byFileType/chromhmmSegmentations/ChmmModels/class1Models_50states/] (file format [EID]/[EID]_50_segments.bed.gz); WGBS fractional methylation, 37 epigenomes [http://egg2.wustl.edu/roadmap/data/byDataType/dnamethylation/WGBS/FractionalMethylation.tar.gz]; DHS narrow peaks, 53 epigenomes [http://egg2.wustl.edu/roadmap/data/byFileType/peaks/consolidated/narrowPeak] (file format [EID]-DNase.macs2.narrowPeak.gz); H3K27ac narrow peaks, 98 epigenomes [http://egg2.wustl.edu/roadmap/data/byFileType/peaks/consolidated/narrowPeak/] (file format [EID]-H3K27ac.narrowPeak.gz); strand-agnostic, unnormalized mRNA signal coverage [http://egg2.wustl.edu/roadmap/data/byDataType/rna/signal/unnormalized_wig/strandagnostic/] (file format [EID].wig.gz) and normalization factors [http://egg2.wustl.edu/roadmap/data/byDataType/rna/signal/unnormalized_wig/all.EGID.N.readlength], 56 epigenomes; and histone modification ChIP-seq and DHS signal fold enrichment ratios over input [http://egg2.wustl.edu/roadmap/data/byFileType/signal/consolidated/macs2signal/foldChange/] (file format [EID]-[mark].fc.signal.bigwig) for H3K4me1, H3K4me3, H3K9me3, H3K27me3, and H3K36me3 (127 epigenomes each), H3K9ac (62 epigenomes), H3K27ac (98 epigenomes), and DHS (53 epigenomes). mm10 chromHMM assignments and WGBS CpG methylation levels were downloaded from the ENCODE data portal [https://www.encodeproject.org/]; see Supplementary Table 4 for unique accessions. The source data underlying all figures is provided in the source data file (SourceData.tar.gz), except for Supplementary Fig. 21. A reporting summary for this Article is available as a Supplementary Information file.

## Source data

Source Data

## Code availability

Software used in this study includes R version 3.3.0 and R packages plyr (1.8.4), reshape2 (1.4.3), ggplot2 (3.1.0), gridExtra (2.2.1), RColorBrewer (1.1.2), scales (1.0.0), mgcv (1.8.24), extraFont (0.17), and rcompanion (2.0.10); python versions 2.7, 3.6.5, and 3.7; bedtools (2.27.1); bedops; kentsrc; kentUCSC; htslib; numpy; pandas; HOMER (4.9); and GREAT (v3). Custom code used in this study is available at https://github.com/epehrsson/TE_landscape.
