# Supplementary material for: The epigenomic landscape of transposable elements across normal human development and anatomy
Source: Nat Commun. 2019 Dec 10;10:5640. doi: 10.1038/s41467-019-13555-x (PMC6904449; doi:10.1038/s41467-019-13555-x)
Supplement: Supplementary file 1 — Supplementary Information [file 41467_2019_13555_MOESM1_ESM.pdf]

Supplementary Information

For “The epigenomic landscape of transposable elements across normal human development and anatomy”, Pehrsson *et al*

Supplementary Discussion

Class and CpG density vs. repression mechanism

To determine the relative influence of TE class and CpG density on the mechanism of epigenetic repression of the TE, we created generalized linear models with a quasi-Poisson distribution for the response (i.e., number of epigenomes in the 9_Het or hypermethylated state) and TE class and CpG density as predictors (Supplementary Table 2). Adding CpG density as a predictor significantly improves the 9_Het model with just TE class (p-value <2e-16, ANOVA (analysis of variance) with F test), and both predictors are highly significant (coefficient p-value <2e-16). The same is true for the hypermethylation model. Therefore, although the two terms are not completely independent, they are also not redundant.

Although both predictors are significant, class has a larger effect size for both epigenetic states, particularly the 9_Het heterochromatin state. For the 9_Het model, being a SINE class versus LTR class TE reduces the number of epigenomes the TE is annotated with the state by 65% on average, despite controlling for CpG density. In contrast, increasing the CpG density by 8 CpGs per kbp (the average difference between the two classes) decreases it by only 3%. For the hypermethylation model, being a SINE class TE increases the number of epigenomes the TE is annotated with the hypermethylated state by 9%, while increasing the CpG density by 8 CpGs per kbp gives an increase of 2%.

Alternative chromHMM annotation methods

To determine the impact of chromHMM annotation method on the likelihood an individual TE is annotated with each epigenetic state (in each or any epigenome), we repeated the analyses with two alternative methods: 1) using only TEs overlapping the center of 200bp chromHMM annotation windows and 2) using only TEs overlapping the center of chromHMM annotation blocks. The number of TEs annotated with each state per epigenome is far lower when considering only TEs overlapping block centers, and as anticipated, the reduction is correlated with the median block size of the state. In contrast, the reduction is lower and more consistent across states when considering TEs overlapping 200bp bin centers (median 70-87% by state). Because chromHMM annotation windows are in the same location in each epigenome and only 82% of TEs overlap window centers, the expected reduction is to 82% of the original result. However, the reduction is greater for some smaller states, suggesting that TEs that do not overlap a 200bp bin center contributed disproportionately to these totals using the standard annotation method.

Requiring TEs to overlap the center of a chromHMM annotation block also results is lower numbers annotated with each state in at least one Roadmap epigenome (29-60% the original number), although the correlation with block size is much less apparent. Requiring TEs to overlap 200bp bin centers gives 73-86% the number with the standard annotation method.

TEs annotated with a state in all epigenomes

The three Alu elements in the 1_TssA state in all 127 epigenomes fall within the promoter regions of *MED18* (first intron), *SF3B5* (274bp upstream), and *DNAJC14/TMEM198B* (first intron/131bp upstream).

The sole TE always overlapping an H3K27ac peak summit is an intergenic HERVIP10FH-int element (chr17:45142929-45145735, 16kb upstream of *ARL17A/B*). It overlaps the transcription start site of a GENCODE v19 lincRNA (RP11-156P1.3, AC068152.1) and exhibits chromHMM marks typical of an actively transcribed gene in almost all epigenomes, as well as overlapping a DHS peak summit in all.

312 TEs consistently overlap DHS peak summits. 59% are intergenic. Of the 15 TEs >50kb from the nearest RefSeq gene, two (LTR12C, chr14:53683100-53684767 and LTR12E, chr5:34244388-34245472) are promoters for a GENCODE v19 lincRNA (AL365295.1) and a pseudogene (AC138409.2), respectively, but the rest overlap no known transcript in either database.

Interestingly, most of the 89 TEs in the 5_TxWk state in all epigenomes are located within several small regions, including a 1.6kb region encompassing 33 MLT1D elements on chrX (213603- 215219) within an intron of *PLCXD1*,

although there are a few SVA_D, Alu, and L1 copies that are isolated. The 89 TEs are intronic (n=60) or intergenic (n=29), although 15 also overlap promoters, and 2 also overlap 3'UTRs.

TEs annotated with multiple states per epigenome

On average, each individual TE is annotated with at most 1 ± 1 chromHMM states in a single epigenome (median/IQR; TEs overlapping bin centers only; see Methods; Figure 3a-b), and this number correlates significantly with TE length (Spearman correlation, p-value < 0.001, rho = 0.64), which can range from 11 to 18,042 bp. Only 31% of TEs overlapping chromHMM bin centers are ever annotated with more than one state in a single epigenome. At the upper extreme, three TEs are annotated with up to 8-10 states in a single epigenome, all of which are over 6.7kb in length (99.98% percentile of length: HERV3-int (chr7:64451293-64459721) and HERVS71-int (chr3:101351443-101360352) elements in HepG2 (E118), and an L1PB1 element (chrX:115076380-115083102) in HUES6 ESCs (E015)). They overlap ZNF, ERV, or non-coding genes only.

Of all instances of TEs overlapping bin centers in a single epigenome, 99% are annotated with only one chromHMM state, and 96% of those with >1 state are annotated with only two states. In contrast, 24% of TE instances that overlap any CpG overlap a CpG in more than one methylation state, and 3.5% overlap CpGs in more than two states.

The specific chromHMM states most likely to co-exist on the same TE within a single epigenome reflect both chromHMM state definitions (e.g., histone modifications) and biological function (Supplementary Figure 8d). For example, the genic enhancer (6_EnhG) state is characterized by the presence of both H3K4me1 and H3K36me3 modifications and is most likely to be co-annotated with the transcribed states (4_Tx/5_TxWk, characterized by H3K36me3, 4% each of all TEs annotated as 6_EnhG) and the enhancer state (7_Enh, characterized by H3K4m3e1, 2% of all TEs annotated as 6_EnhG).

Epigenetic state dynamics of shuffled TEs

To establish a background for TE epigenetic state dynamics, we repeated the analyses with 10 iterations of shuffled TEs. More shuffled TEs are in all 15 chromHMM states across epigenomes (mean 164 vs. 9 true TEs), and shuffled TEs ever in the 2_TssAFlnk, 5_TxWk, 13_ReprPC, and intermediately methylated states are in more states overall than are true TEs. Shuffled TEs ever in the poised promoter states (10_TssBiv and 11_BivFlnk; 2.5% per iteration vs. <1% real TEs) are in the corresponding active states in 13 to 29% of epigenomes and the Polycomb repressed 13_ReprPC state in 16% of epigenomes, a higher proportion than for true TEs. In conclusion, real TEs are slightly less dynamic than expected, although the differences are small.

Epigenetic annotation variability by epigenome

The total number of bases or CpGs annotated with each epigenetic state varies substantially by epigenome, with some tissue-specific patterns (e.g., low 1_TssA annotation in blood epigenomes, high H3K27ac annotation in brain epigenomes) (Supplementary Figure 1). Some epigenomes are also outliers. For instance, E017 (IMR90) is severely hypomethylated relative to other epigenomes, with 18% of its CpGs hypomethylated and 24% of its CpGs intermediately methylated (versus median 10% each across all epigenomes), and the low methylation disproportionately affects TEs compared to other epigenomes (Figure 4a). 42% of the CpGs in E085 (Fetal Intestine Small) are missing methylation data (versus median 4%), and 23% of E034 (Primary T cells from peripheral blood) is in the weakly Polycomb repressed state (14_ReprPCWk) versus a median of 8%.

The proportion of each state within TEs is positively correlated with the total number of bases or CpGs annotated with the state for nine of the chromHMM states (states 1-4, 6, 8, and 13-15) and the hypomethylated state (Bonferroni-corrected p-value < 0.05, Spearman correlation), suggesting that TEs preferentially contribute more of those states in epigenomes with greater annotation with the state overall. However, the metrics are significantly negatively correlated for the 5_TxWk state and the hypermethylated and missing CpG data states, suggesting the opposite, that bases in these states are preferentially added to the non-TE genome.

The median width of a chromHMM block or DHS/H3K27ac peak also varies by state and by epigenome (Kruskal-Wallis test p-value = 0 across all epigenomes for each state; Supplementary Figure 22b). The number of blocks/peaks also varies considerably by epigenome, with the largest coefficient of variation in 3_TxFlnk (0.96) and the lowest in 5_TxWk

(0.20). While most states exhibit a positive correlation between the number and median length of the state blocks, indicating an increase in the total proportion of the genome annotated with that state, the 5_TxWk and 15_Quies chromHMM states and DHS and H3K27ac peaks exhibit a negative correlation (Spearman correlation, Bonferroni-corrected p-value < 0.05). For these states, a TE may be more likely to overlap a peak summit and be counted as annotated with the state in epigenomes with more, shorter peaks, even if the total bases annotated with the state does not change.

Tissue-specific subfamily enrichment

457 of the 968 TE subfamilies are enriched at least once in an active regulatory or transcribed chromHMM state, hypomethylated or intermediately methylated states, or in DHS or H3K27ac peak overlap (active states). Other class subfamilies are overrepresented in the 7_Enh and intermediately methylated states, as are DNA subfamilies among 4_Tx- and 6_EnhG-enriched subfamilies.

Four LINE subfamilies, X3_LINE (RTE-BovB family), HAL1-2a_MD (L1), X1_LINE (CR1), and X6B_LINE (CR1), together account for 70% of the 103 enrichments of LINE subfamilies in active states. X1_LINE is specifically enriched in ESCs in the 7_Enh state but Brain epigenomes in overlap with DHS peaks.

Subfamilies including LTR3B_ and MER44C exhibit enrichment in the 7_Enh state and DHS peak overlap in a cluster similar to that observed for MER121, but that includes more ENCODE cell lines of ectodermal origin and epithelial epigenomes, including melanocytes and keratinocytes in addition to fibroblasts.

Subfamilies in the 5th percentile of age (Jukes-Cantor distance <0.076) are also enriched in active epigenetic states. Most strikingly, the primate-specific subfamilies SVA_C, SVA_D, SVA_E, and SVA_F are enriched in the weakly transcribed 5_TxWk state in blood epigenomes more often than expected, although this pattern is likely due to a relative depletion of the 5_TxWk state in blood (Supplementary Figure 1), as the subfamilies exhibit a uniform number of TEs in the 5_TxWk state across all epigenomes. LTR13 and LTR13A are also enriched in blood epigenomes in the 1_TssA state and overlap with H3K27ac peaks and in the 7_Enh state, respectively, while LTR10C is enriched in the 1_TssA state in skin epigenomes.

Even when a subfamily is enriched, the median proportion of members in the state in a single epigenome is low (<15% for most states) and is never more than 53% for an active regulatory or transcribed chromHMM state (maximum UCON25, 23 members, 7_Enh, E010, H9 Derived Neuron Cultured Cells) or 58% for a methylation state (MER9B, 18 members, intermediate methylation, E094, Gastric), although it reaches 79% for DHS peaks (LTR6B, 121 members, E004, H1 BMP4 Derived Mesendoderm Cultured Cells).

Very large subfamilies (in terms of total length and number of members) are less likely to be enriched in active states than smaller subfamilies despite encompassing a large proportion of the state, because there is a higher expectation of overlap with the state by random chance. Subfamilies can encompass up to 3% of a chromHMM state (4_Tx, AluSx, E085 and 6_Enh, L2a, E004) and 11% of a methylation state (missing CpG data, AluY, E003) without being enriched. Indeed, there are 9,233 instances where a subfamily represents >1% of a state, and in only 863 (9%) is the subfamily also enriched. 25 subfamilies can encompass >1% of an active state, 21 of which belong to the Alu, MIR, L1, and L2 families (Supplementary Figure 16). Although size is a major predictor of large contribution to an epigenetic state, there is family-level variation as well. While the largest Alu, MIR, and L2 subfamilies all frequently represent >1% of the 4_Tx and 5_TxWk transcribed states, Alu subfamilies are less likely than MIR and L2 subfamilies to represent >1% of the active regulatory states. Furthermore, when large Alu subfamilies do represent >1% of the enhancer (6_EnhG and 7_Enh) states and the weakly transcribed 5_TxWk state, it is preferentially in blood epigenomes. In contrast, L1 subfamilies rarely reach >1% of active states.

As expected, the median number of members in the state when a subfamily represents >1% of a state is >100 for all but 10_TssBiv, although the median proportion of members is still frequently <5% due to the large size of some of the subfamilies. A subfamily can represent >1% of a state with ≤10 members in the state for the 3_TxFlnk, 6_EnhG, poised regulatory, 13_ReprPC, and missing CpG states due to the small overall proportion of the genome annotated with those states in some epigenomes.

Features affecting individual TE epigenetic profiles

The sex chromosomes and chromosome 19 are outliers in terms of overall chromHMM profile (though less so for other epigenetic marks; Supplementary Figure 17a-d), which is reflected in the average epigenetic profile of TEs on those chromosomes. Chromosome 19 TEs are more often annotated with several active states, including the 1_TssA promoter and 8_ZNF/Rpts states, hypomethylation, and expression RPKM > 1 (Supplementary Figure 17e). This is likely due to higher gene density on that chromosome, and indeed, 6% of chromosome 19 TEs (n=6,816) overlap protein-coding promoters versus 1.7% of all TEs. In contrast, chromosome Y TEs are more often in the 15_Quies state and missing methylation data, while chromosome X TEs are more often intermediately methylated, likely reflecting X-inactivation in females.

In addition to sequence features, overlap with genes greatly influences the TE epigenetic profile, with TEs that overlap promoters annotated with the 1_TssA state in 39% more epigenomes than those that do not, while TEs within introns are in the 4_Tx transcribed and genic enhancer (6_Enh) states in 14-17% more epigenomes (Supplementary Figure 18a). However, overlap with genes has relatively little impact on the number of epigenomes a TE overlaps a DHS or H3K27ac peak or is hypomethylated, likely because these epigenetic marks characterize intergenic enhancers as well.

Orthologous TEs

Although each hg19 TE is lifted over to only one set of mm10 coordinates, 0.6% of (n=1,568) hg19->mm10 coordinates are shared between hg19 TEs (thus, 266,646 hg19->mm10 coordinates for 269,096 hg19 TEs; up to 11 hg19 TEs per hg19->mm10 coordinate). 9% of both hg19 TEs and hg19->mm10 coordinates correspond to more than one mm10 ortholog (up to 10 mm10 orthologs per hg19 TE), and 8% of mm10 orthologs correspond to more than one hg19 TE or hg19->mm10 coordinate (up to 36 hg19->mm10 coordinates and 41 hg19 TEs). This is likely due to annotation differences between the species; the median length of hg19 TEs with mm10 orthologs is 220 bp, hg19->mm10 coordinates 207 bp, and mm10 orthologs 155 bp.

71% of hg19 TEs with an mm10 ortholog (n=190,143) and 62% of mm10 orthologs (n=166,883) overlap CpGs; 140,185 pairs of orthologs representing 131,306 hg19 TEs and 130,081 mm10 TEs both overlap CpGs. The median number of CpGs per TE is 2 for both hg19 and mm10 orthologs.

Even when subfamilies are shared between human and mouse, they may have different numbers of members. The median ratio of mm10 subfamily members to hg19 subfamily members is 0.27 (0.26 for members with CpGs), or roughly 4X as many members in human than in mouse.

Supplementary Figures

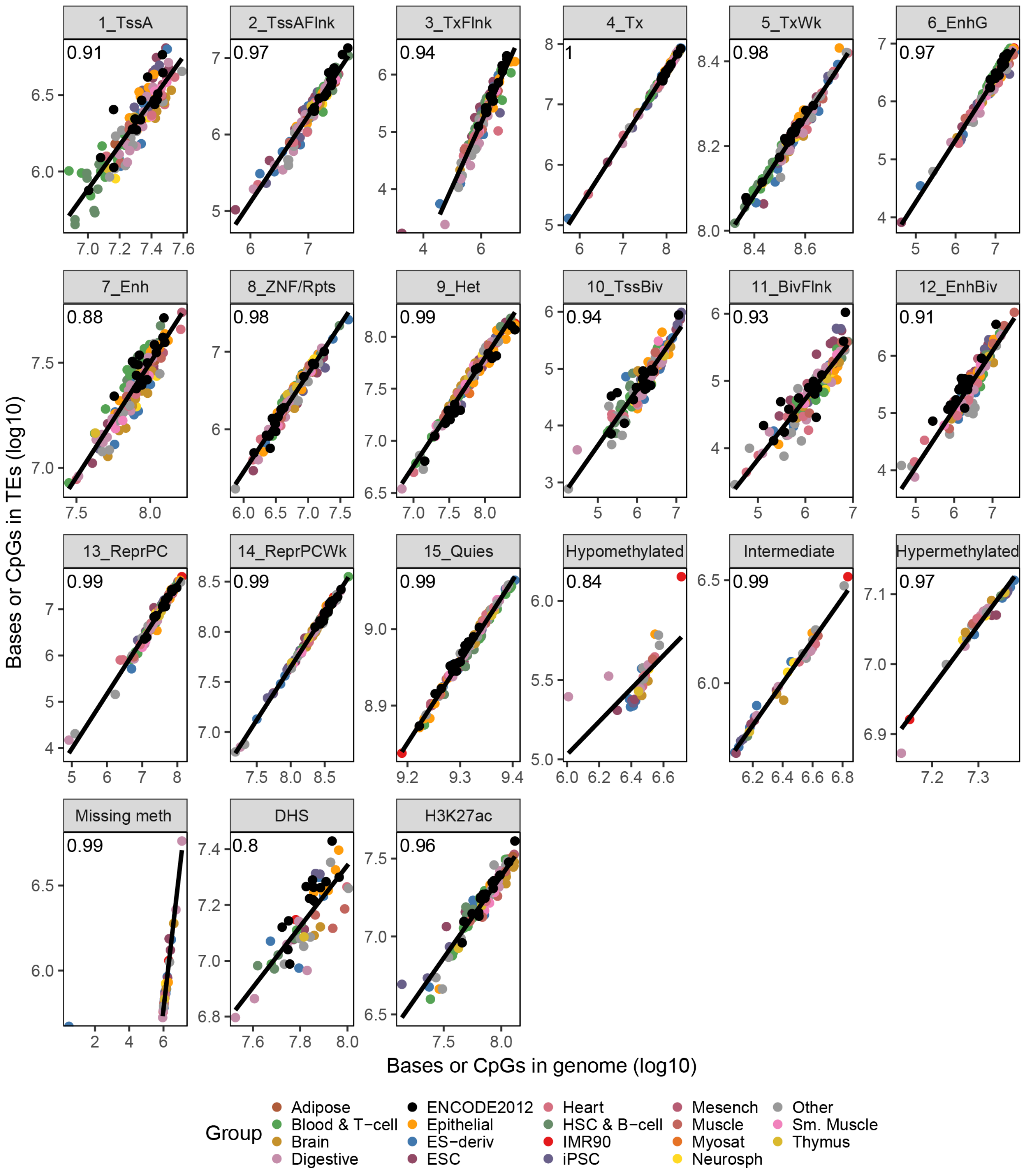

Supplementary Figure 1. Variation in epigenetic profile by epigenome. Total bases or CpGs in each epigenetic state by epigenome, for the entire genome (x-axis) and within TEs (y-axis). Each epigenome is represented by a circle, colored by Roadmap group. One epigenome (E012) has no CpGs within TEs missing methylation data. Spearman's rho for the correlation between the two values is listed for each state. chromHMM states n=127 epigenomes, methylation states n=37, DHS n=53, H3K27ac n=98.

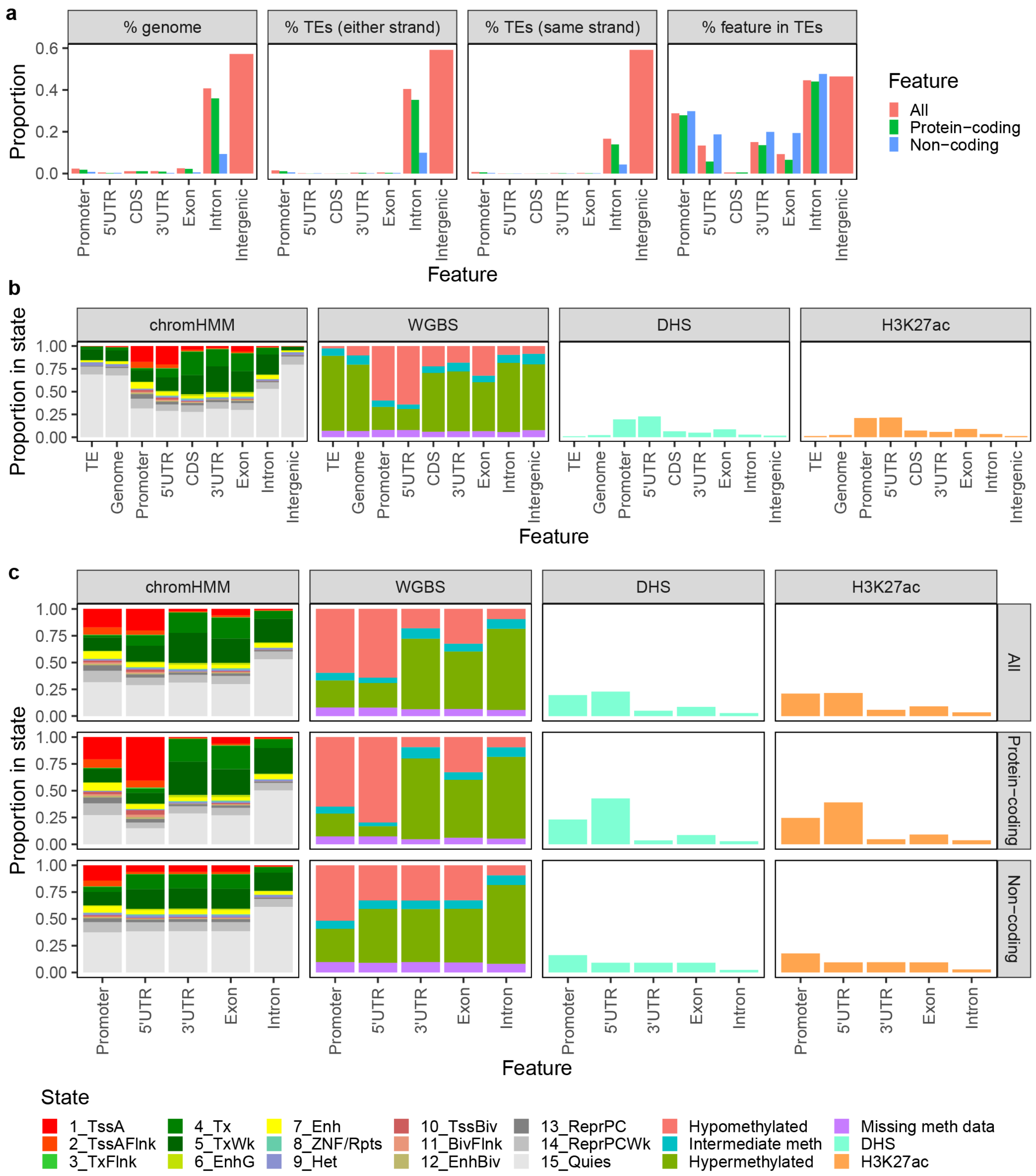

Supplementary Figure 2. Overlap of TEs with genic features and epigenetic states across all Roadmap epigenomes. **a** For each RefSeq feature, the proportion of the genome it encompasses (% genome), the proportion of unique TE bases overlapping the feature (on either strand or the same strand; % TEs), and the proportion of the feature overlapping TEs (% feature in TEs). Features are presented in aggregate (All) and split into protein-coding and non-coding features (see Methods). **b** Proportion of bases or CpGs within TEs, the entire genome, and RefSeq features annotated with each epigenetic state, averaged across all epigenomes with data for that technique (chromHMM states n=127 epigenomes, methylation states n=37, DHS n=53, H3K27ac n=98; vs. Figure 1a, summed across all epigenomes). The color legend is shared with **c**. **c** Proportion of bases or CpGs within each RefSeq feature annotated with each epigenetic state, summed across all epigenomes with data for that technique and split by protein-coding and non-coding features. The first row is the same as in Figure 1a.

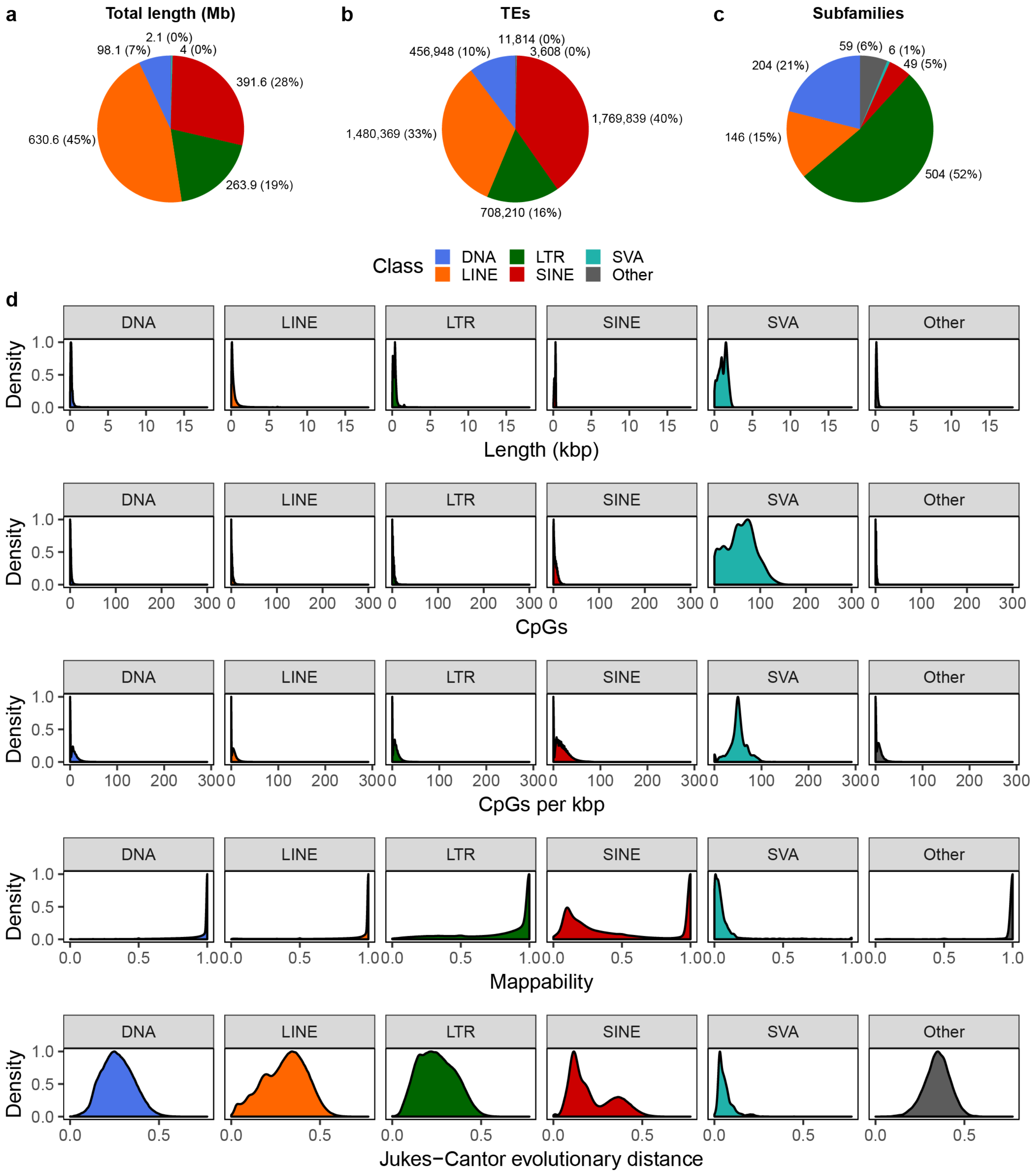

Supplementary Figure 3. TE class characteristics. **a-c** Proportion of TE **a** bases, **b** individual elements, and **c** subfamilies in each TE class. **d** Scaled density plots of individual TE length, number of CpGs, CpG density, 36bp mappability, and Jukes-Cantor evolutionary distance from consensus, by class (DNA n=456,948 TEs, LINE n=1,480,369, LTR n=708,210, SINE n=1,769,839, SVA n=3,608, Other n=11,814). Distribution color is based on class (facet).

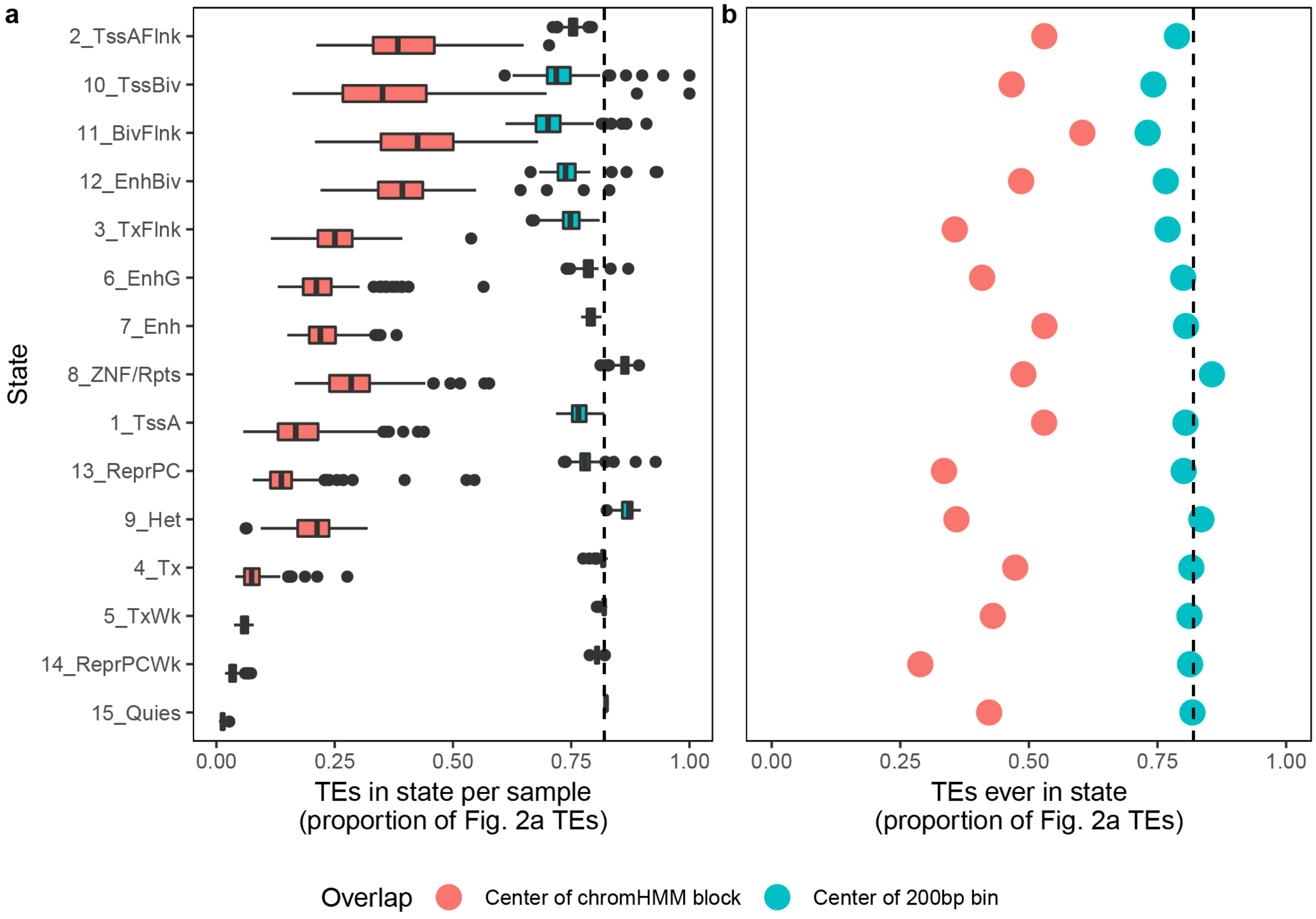

Supplementary Figure 4. Individual TE potential to be annotated with a chromHMM state using alternative annotation rules. **a-b** TEs annotated with a chromHMM state are limited to those overlapping the center of a chromHMM annotation block or 200bp annotation bin. Dashed lines represent the proportion of TEs that overlap the center of any 200bp bin (82%). **a** The proportion of all TEs (4,430,788 TEs) annotated with the state per epigenome (n=127 epigenomes), divided by the proportion from Figure 2a (boxplots). The y-axis is ordered by increasing median block size across all epigenomes. Boxplot elements: center line, median; box limits, first and third quartiles; whiskers, maximum value ≤ 1.5*IQR from box limits; points, outliers. **b** The fraction of TEs (n=4,430,788 TEs) annotated with the state in at least one epigenome (of 127 epigenomes), divided by the proportion from Figure 2a (red dots). The y-axis is shared with **a**.

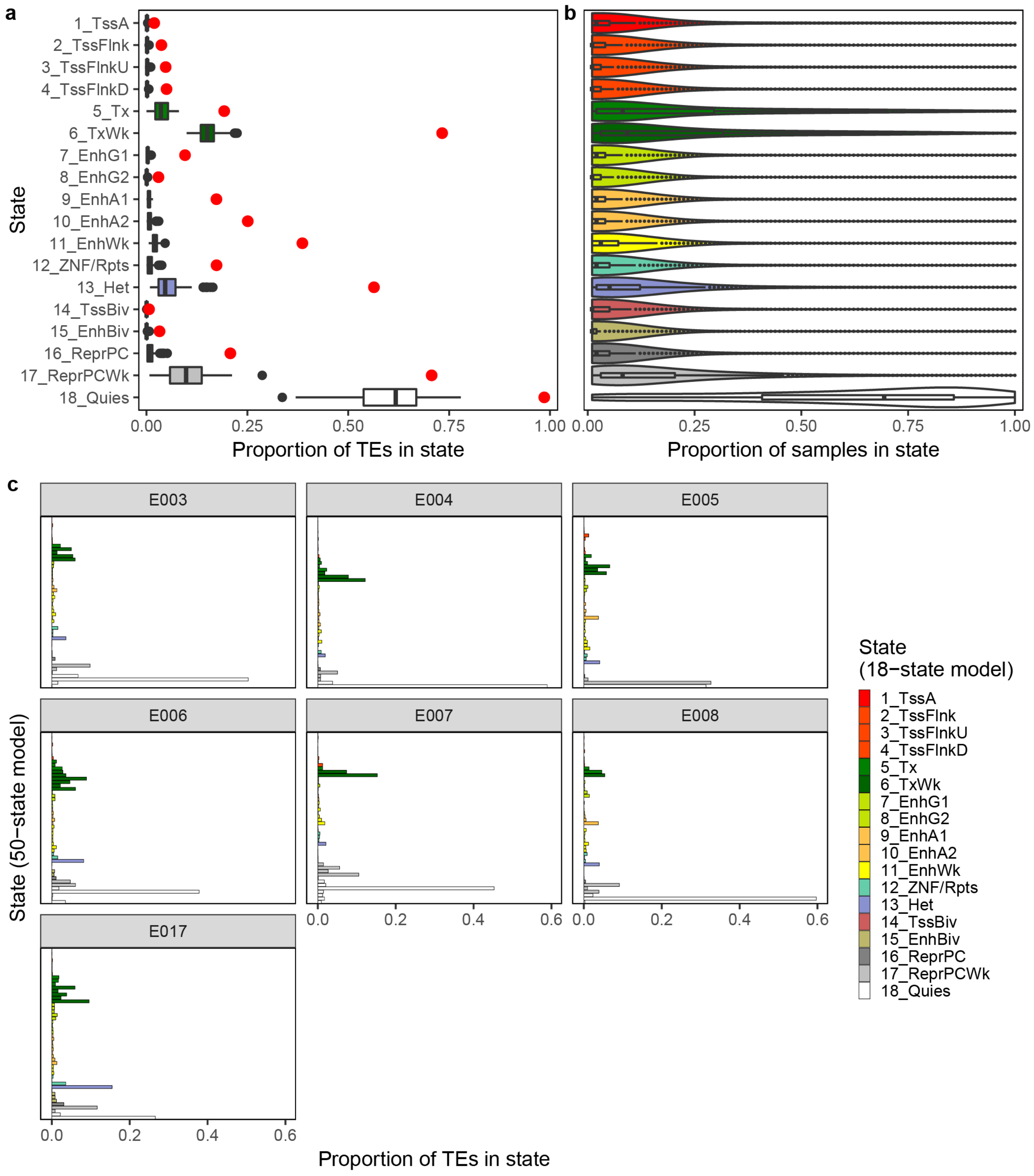

Supplementary Figure 5. Individual TE potential to be annotated with a chromHMM state using additional chromHMM models. **a-b** TEs annotated with the 18-state chromHMM model. State colors were defined by the Roadmap Project and correspond to the y-axis. **a** Boxplots indicate the proportion of all TEs (4,430,788 TEs) annotated with the state per epigenome (n=98 epigenomes). Red dots are the fraction of TEs annotated with the state in at least one epigenome. **b** For TEs annotated with the state in at least one epigenome (Supplementary Figure 5a, dots), the proportion of epigenomes the TE is annotated with the state. The y-axis is shared with **a**. **a-b** Boxplot elements: center line, median; box limits, first and third quartiles; whiskers, maximum value ≤ 1.5*IQR from box limits; points, outliers. **c** TEs annotated with 50-state chromHMM models trained independently on 7 epigenomes. Bar plots indicate the proportion of TEs (4,430,788 TEs) annotated with the state (top to bottom, E1-E50) in each epigenome, colored by the corresponding 18-state model state (see Methods).

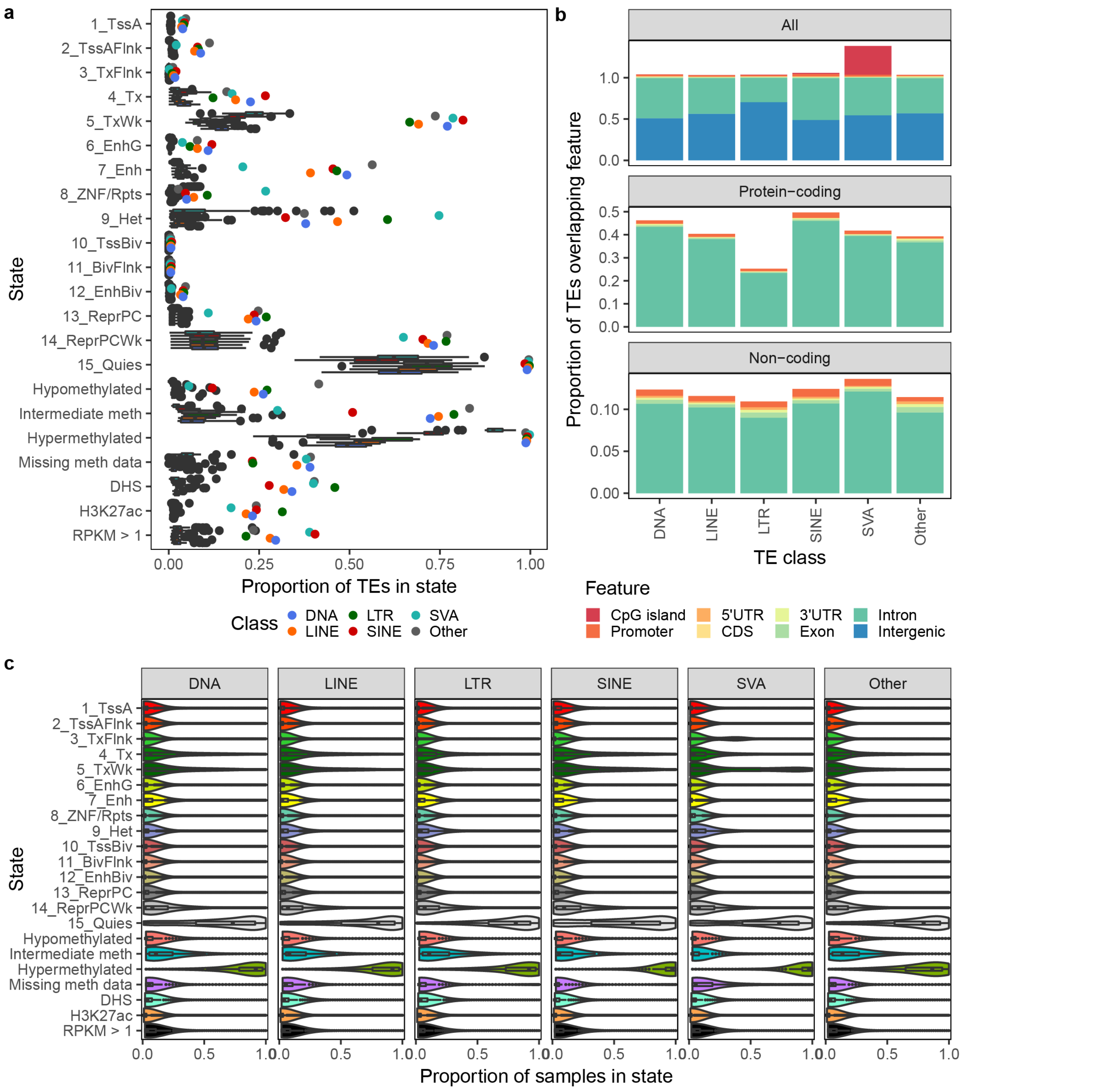

Supplementary Figure 6. Individual TE potential to be annotated with an epigenetic state, by class. **a** Boxplots indicate the proportion of TEs in each class (DNA 456,948 TEs, LINE 1,480,369, LTR 708,210, SINE 1,769,839, SVA 3,608, Other 11,814) annotated with the state per epigenome (chromHMM states n=127 epigenomes, methylation states n=37, DHS n=53, H3K27ac n=98, expression n=56). Dots are the fraction of TEs in each class annotated with the state in at least one epigenome. Boxplots and dots are colored by class. For WGBS states, only TEs with CpGs are included (DNA 275,140 TEs (e.g., 60% of DNA elements), LINE 952,459 (64%), LTR 532,571 (75%), SINE 1,430,171 (81%), SVA 3,519 (98%), Other 6,568 (56%)). **b** Proportion of individual TEs in each class overlapping RefSeq features or CpG islands, split by protein-coding and non-coding features. TEs may overlap multiple features, leading to a total proportion >1. **c** For TEs annotated with the state in at least one epigenome (Supplementary Figure 6a, dots), the proportion of epigenomes the TE is annotated with the state, split by class. Distributions are colored by state (y-axis). **a/c** Boxplot elements: center line, median; box limits, first and third quartiles; whiskers, maximum value ≤ 1.5*IQR from box limits; points, outliers.

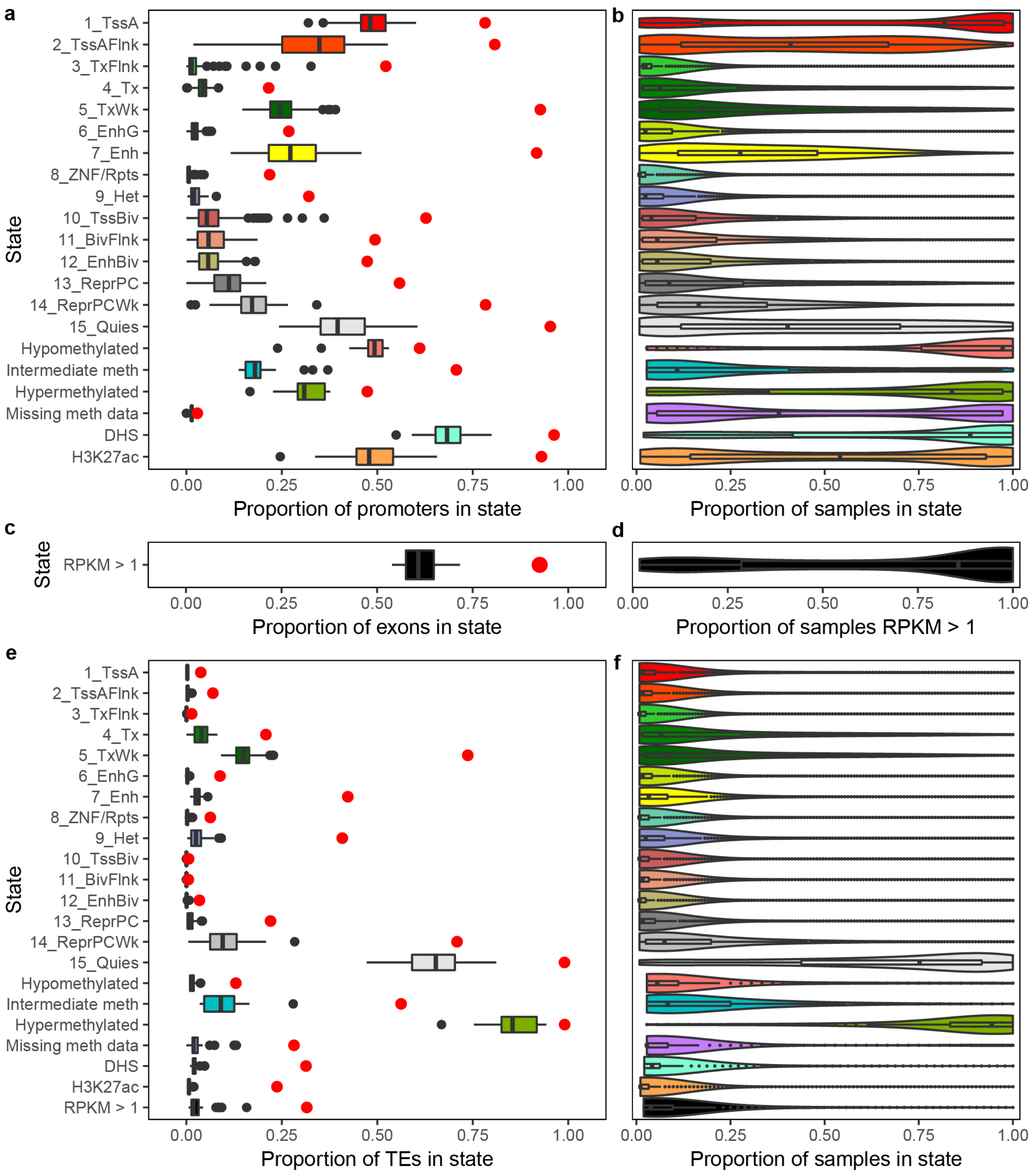

Supplementary Figure 7. Potential for individual promoters and exons to be annotated with an epigenetic state and potential for individual TEs excluding cancer cell lines. **a** Boxplots indicate the proportion of unique RefSeq promoters (34,750 promoters) annotated with the state per epigenome (chromHMM states n=127 epigenomes, methylation states n=37, DHS n=53, H3K27ac n=98). Red dots are the fraction of promoters annotated with the state in at least one epigenome. All promoters overlap CpGs. **b** For promoters annotated with the state in at least one epigenome (Supplementary Figure 7a, dots), the proportion of Roadmap epigenomes the promoter is annotated with the state. The y-axis is shared with **a**. **c** The boxplot indicates the proportion of unique RefSeq exons (252,280 exons) with RPKM >1 per epigenome (n=56 epigenomes). Red dots are the fraction of exons with RPKM >1 in at least one epigenome. **d** For exons with RPKM >1 in at least one epigenome (Supplementary Figure 7c, dots), the proportion of Roadmap epigenomes the exon is RPKM >1. The y-axis is shared with **c**. **e** Boxplots indicate the proportion of TEs (4,430,788 TEs) annotated with the state per epigenome, excluding

five cancer cell lines and IMR90 (remaining epigenomes with chromHMM, n=121 epigenomes; WGBS, n=36; DHS, n=48; H3K27ac, n=92; RNA, n=52). Red dots are the fraction of TEs annotated with the state in at least one of the remaining epigenomes. For WGBS states, only TEs with CpGs are included (3,200,428 TEs). **f** For TEs annotated with the state in at least one epigenome (Supplementary Figure 7e, dots), the proportion of Roadmap epigenomes the TE is annotated with the state, excluding cancer cell lines and IMR90. The y-axis is shared with **e**. **a-f** Boxplot elements: center line, median; box limits, first and third quartiles; whiskers, maximum value ≤ 1.5*IQR from box limits; points, outliers. Boxplots and distributions are colored by state (y-axis).

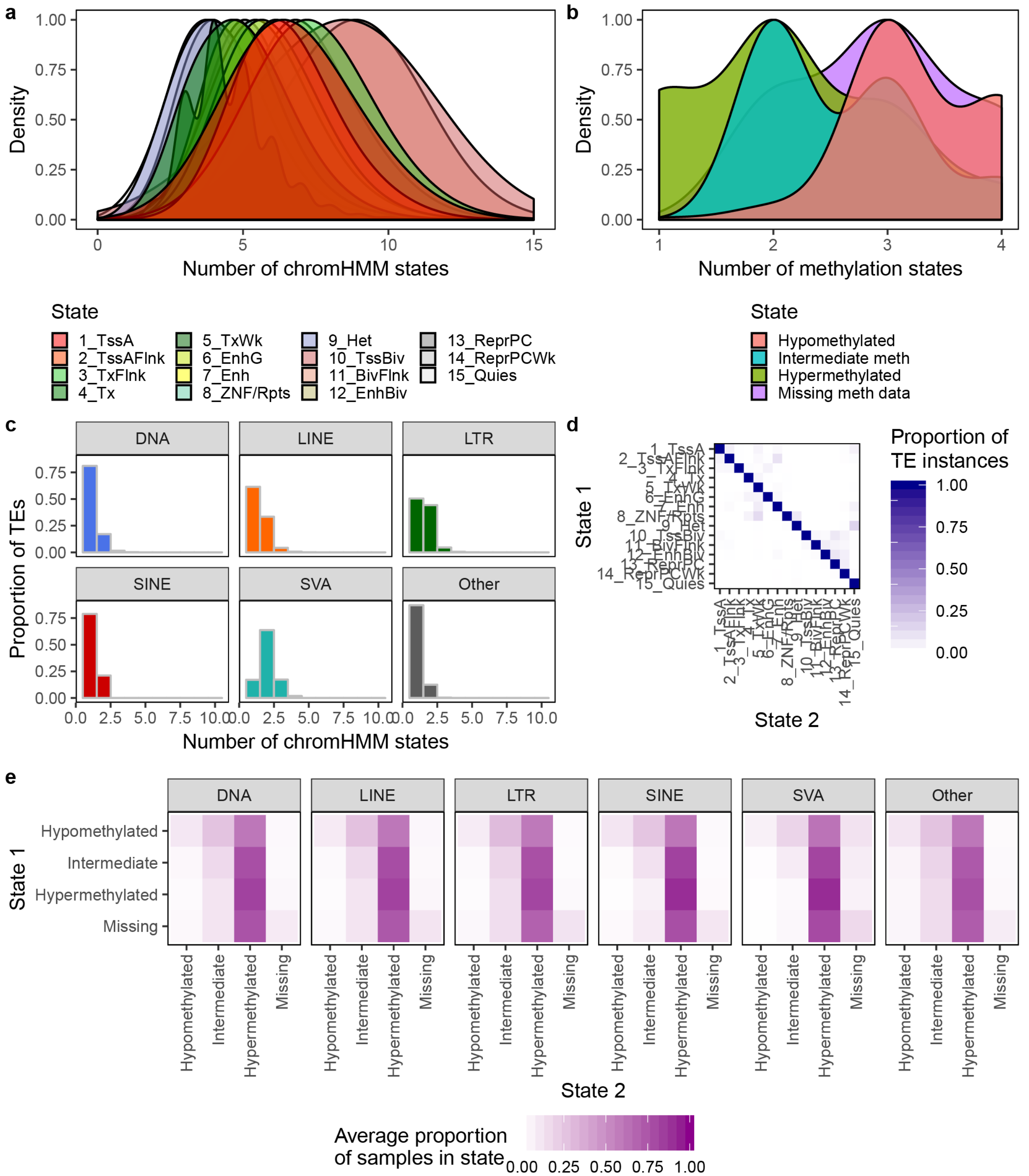

Supplementary Figure 8. TE epigenetic annotations within and across epigenomes. **a** For individual TEs in each chromHMM state in at least one epigenome (Figure 2a, red dots), scaled density plots of the total number of chromHMM states the TE is annotated with across all epigenomes (127 epigenomes). **b** For individual TEs in each methylation state in at least one epigenome (Figure 2a, red dots), scaled density plots of the total number of methylation states the TE is annotated with across all epigenomes (37 epigenomes). **c** For individual TEs in each TE class, the maximum number of chromHMM states with which the TE is annotated in a single epigenome. Histograms are colored by class (facet). Includes only TEs overlapping the center of 200bp chromHMM annotation windows, n=3,623,613 TEs: DNA n=331,443 TEs, LINE n=1,174,979, LTR n=605,833, SINE n=1,499,320, SVA n=3,466, Other n=8,572 (see Methods). **d** The proportion of TE instances in chromHMM State 1 (i.e., one TE in the state in a single epigenome) that are also annotated with chromHMM State 2 in the same epigenome. Includes only TEs overlapping the center of 200bp chromHMM annotation windows**. e** For individual TEs in methylation State 1 in at least one Roadmap epigenome (Supplementary Figure 6a, dots), the average proportion of epigenomes (37 epigenomes) they are annotated with methylation State 2, by class.

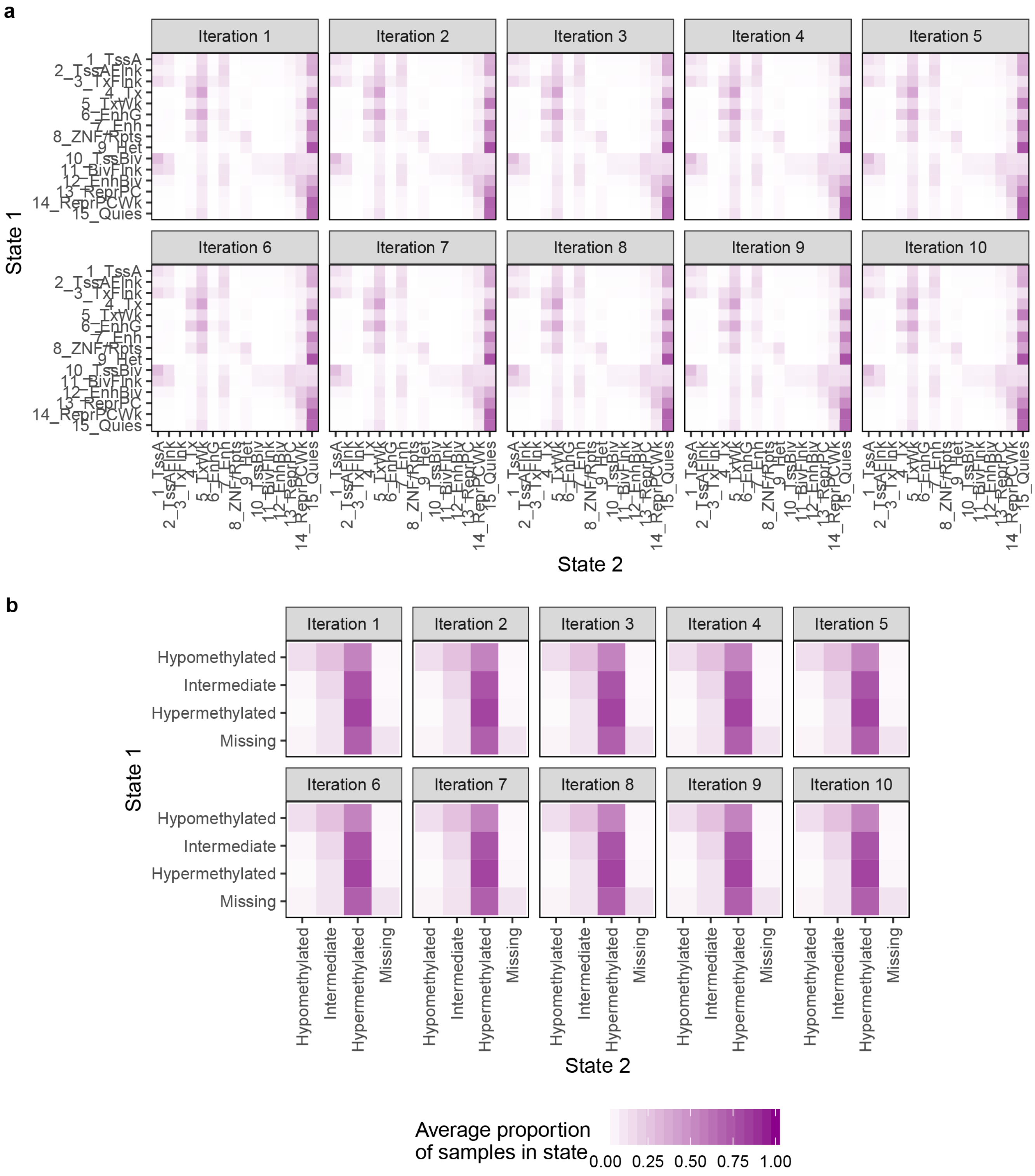

Supplementary Figure 9. Epigenetic state dynamics of shuffled TEs. **a** For TEs in chromHMM State 1 in at least one Roadmap epigenome, the average proportion of epigenomes in which they are annotated with chromHMM State 2 (represented by color scale below Supplementary Figure 9b), for 10 iterations of shuffled TEs. **b** For TEs in methylation State 1 in at least one Roadmap epigenome, the average proportion of epigenomes in which they are annotated with methylation State 2, for 10 iterations of shuffled TEs.

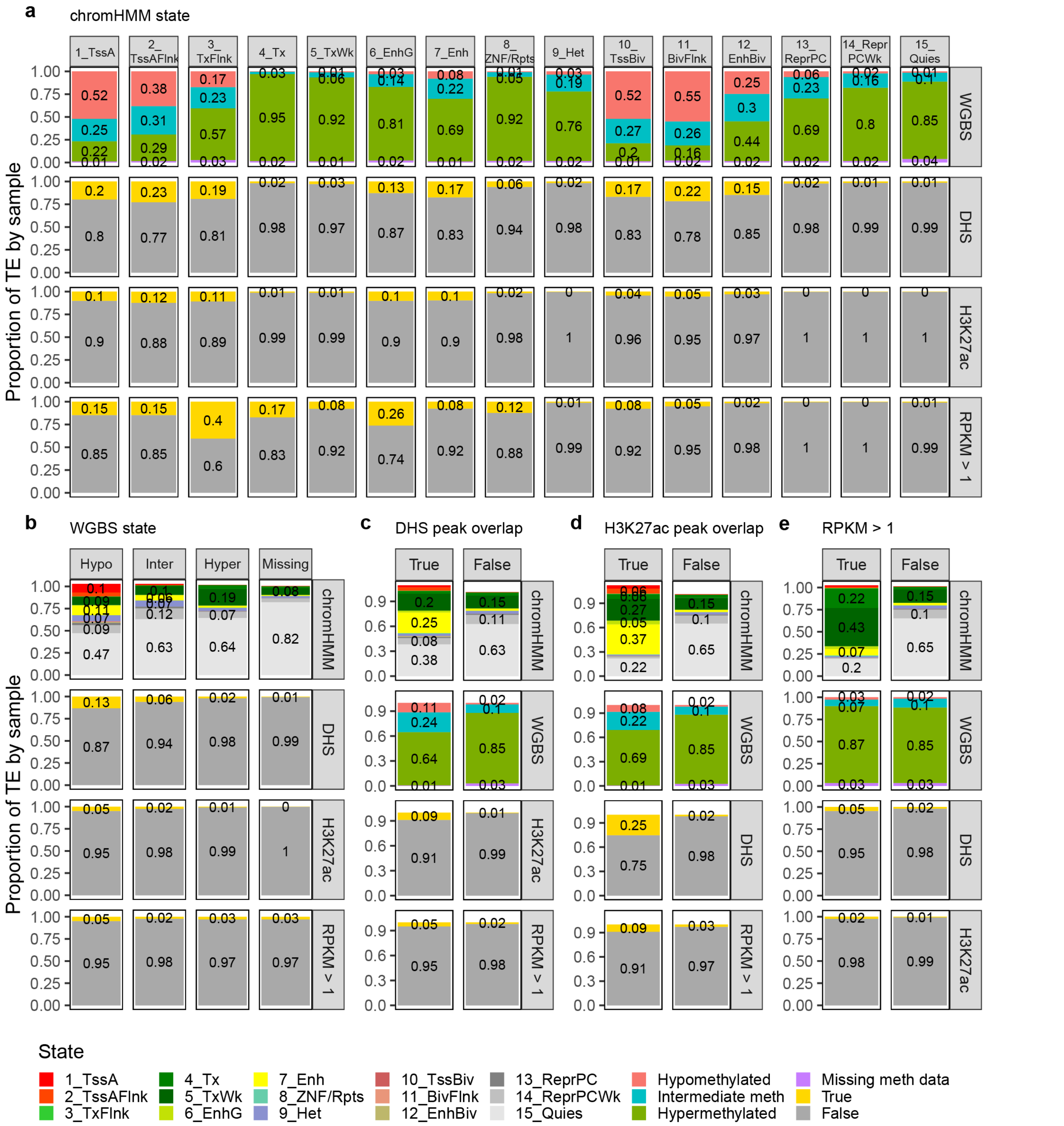

Supplementary Figure 10. Comparison of epigenetic states annotated with different techniques. **a-e** Proportion of TE by epigenome instances in each x-axis state also annotated with each y-axis state, for epigenomes with data for both techniques. For chromHMM and WGBS, colors represent states; for DHS, H3K27ac, and RNA-seq, colors represent True/False for overlap with a peak summit or RPKM >1 (see color legend below figure). Stacked bar plots are labelled with the proportion of TE instances in the y-axis state; only chromHMM states with proportion ≥0.05 are labelled. Chi-squared tests are significant (p-value=0) for all technique pairs. For comparisons of other techniques to chromHMM, the number of TE by epigenome instances is normalized by the total number of TE by epigenome instances in the x-axis state to account for those annotated with more than one chromHMM state. **a** Proportion of TE by epigenome instances in each chromHMM state (x-axis) also annotated with each y-axis state, for epigenomes with data for both techniques (WGBS: 37 epigenomes; DHS: 53; H3K27ac: 98; RNA: 56). **b** Proportion of TE by epigenome instances in each methylation state (x-axis) also annotated with each y-axis

state, for epigenomes with data for both techniques (DHS epigenomes: 17; H3K27ac: 33; RNA: 33). **c** Proportion of TE by epigenome instances overlapping DHS peaks (x-axis) also annotated with each y-axis state, for epigenomes with data for both techniques (H3K27ac epigenomes: 44; RNA: 29). The y-axis title is shared with **b**. **d** Proportion of TE by epigenome instances overlapping H3K27ac peaks (x-axis) also annotated with each y-axis state, for epigenomes with data for both techniques (RNA: 48 epigenomes). The y-axis title is shared with **b**. **e** Proportion of TE by epigenome instances expressed RPKM >1 (x-axis) also annotated with each y-axis state, for epigenomes with data for both techniques. The y-axis title is shared with **b**.

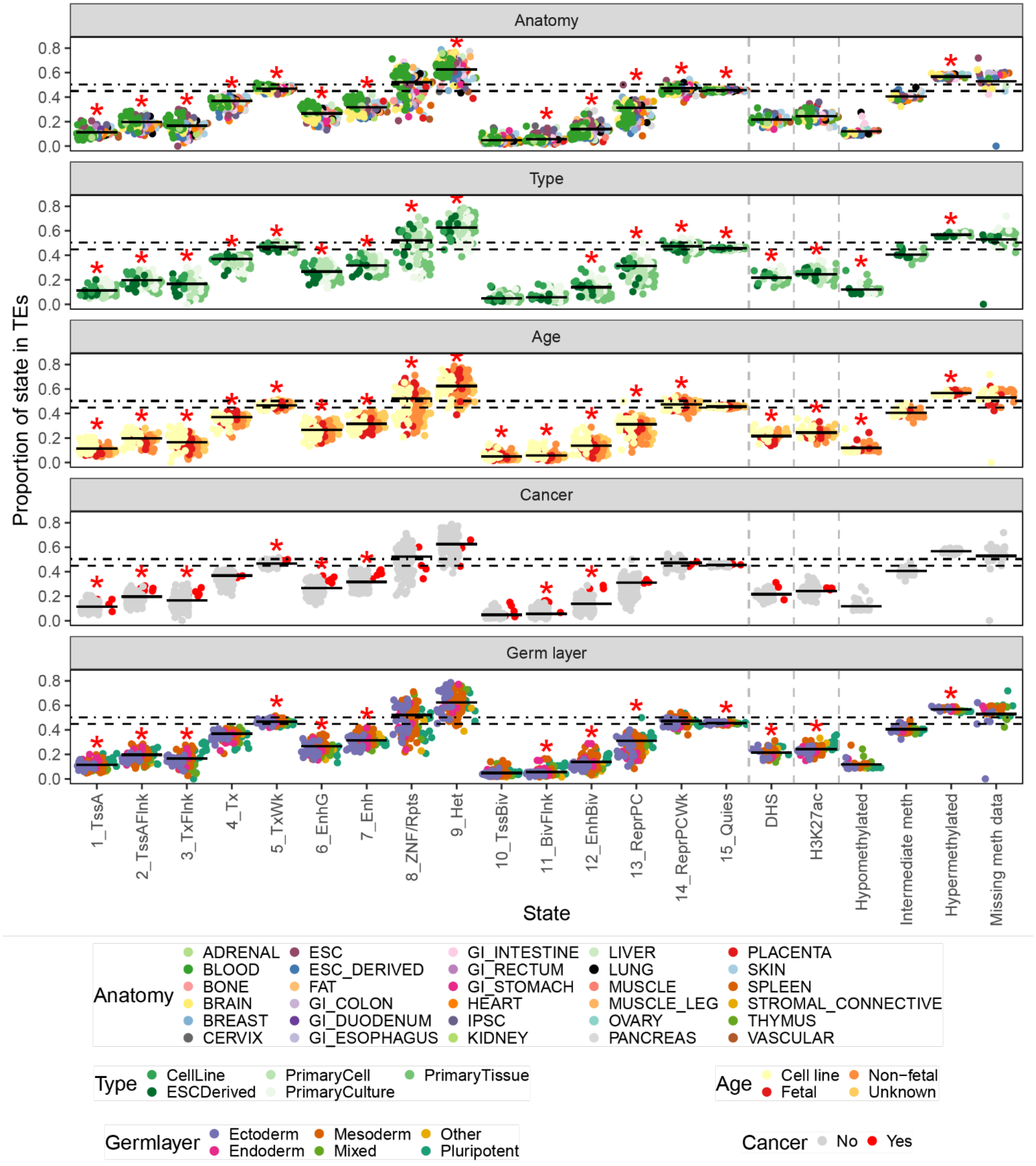

Supplementary Figure 11. Variation in TE contribution to epigenetic states by alternative epigenome classifications. Each facet is Figure 4a (proportion of the epigenetic state within TEs by epigenome) colored by epigenome Anatomy, Type, Age, Cancer, and Germ layer metadata (see Methods, Supplementary Data 3). Solid black lines represent the proportion of the epigenetic state within TEs across all epigenomes (contribution; see Figure 1c). Dashed and dot-dashed lines represent the proportion of genomic bases and CpGs within TEs, respectively. Red stars represent Bonferroni-corrected Kruskal-Wallis test p-value < 0.05 across epigenome categories (chromHMM states n=127 epigenomes, methylation states n=37, DHS n=53, H3K27ac n=98; see Supplementary Data 3 for metadata assignments).

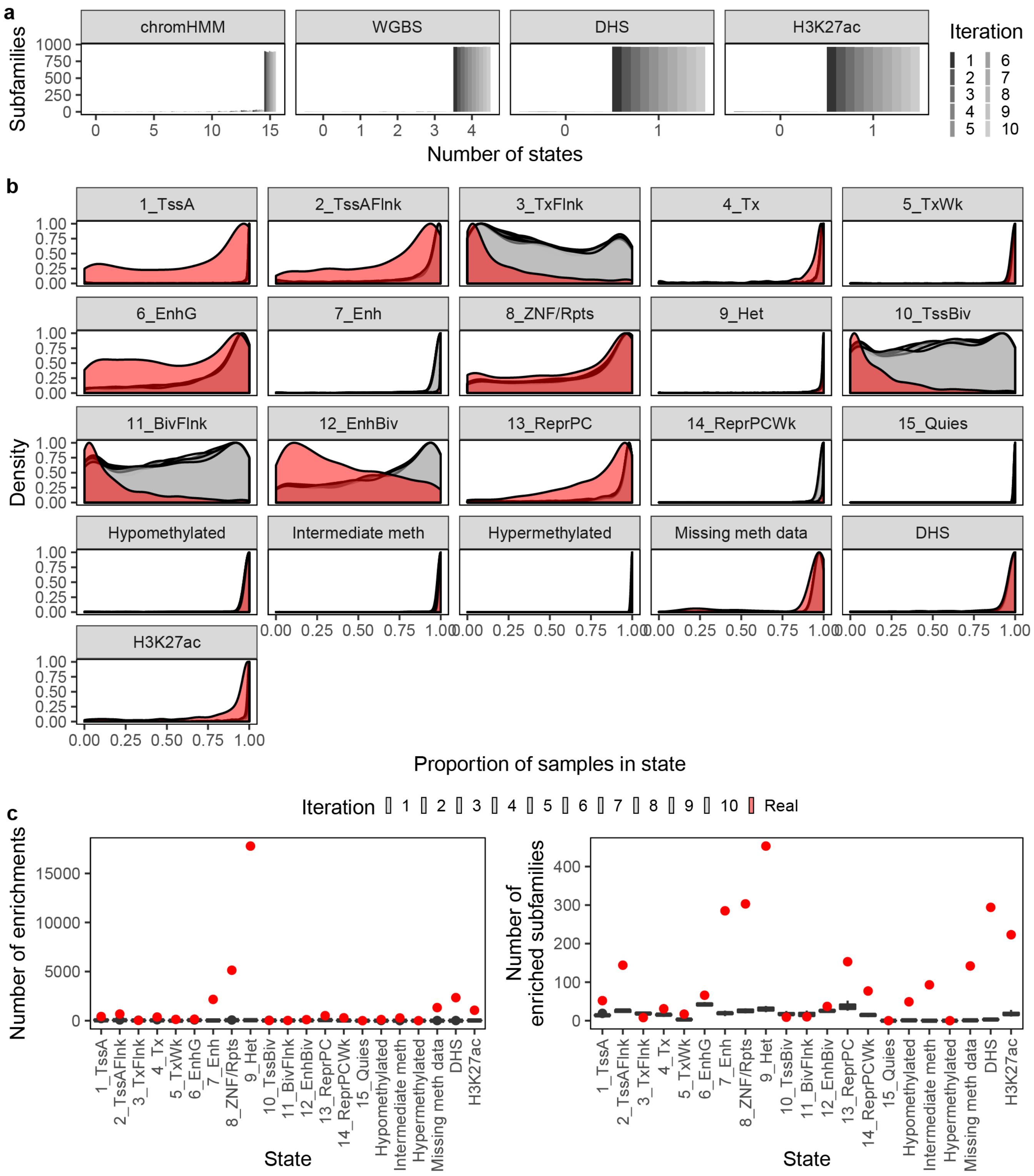

Supplementary Figure 12. Tissue-specific enrichment of shuffled TE subfamilies in epigenetic states. **a** The total number of epigenetic states with which each shuffled TE subfamily is annotated across all epigenomes (n=968 subfamilies, n=966-968 for methylation states), for 10 iterations of shuffled TEs. **b** Scaled density plots of the proportion of Roadmap epigenomes in which each TE subfamily is annotated with each epigenetic state (chromHMM states 127 epigenomes, methylation states 37, DHS 53, H3K27ac 98). Distributions are presented for real TE subfamilies (n=968 subfamilies, n=965 for methylation states) and 10 iterations of shuffled TEs. **c** Number of subfamily enrichments with LOR > 1.5 per state for 10 iterations of shuffled TEs (boxplot, n=10 iterations) and real TEs (red dot). **d** Number of subfamilies enriched LOR > 1.5 at least once per state for 10 iterations of shuffled TEs (boxplot, n=10 iterations) and real TEs (red dot). **c-d** Boxplot elements: center line, median; box limits, first and third quartiles; whiskers, maximum value ≤ 1.5*IQR from box limits; points, outliers.

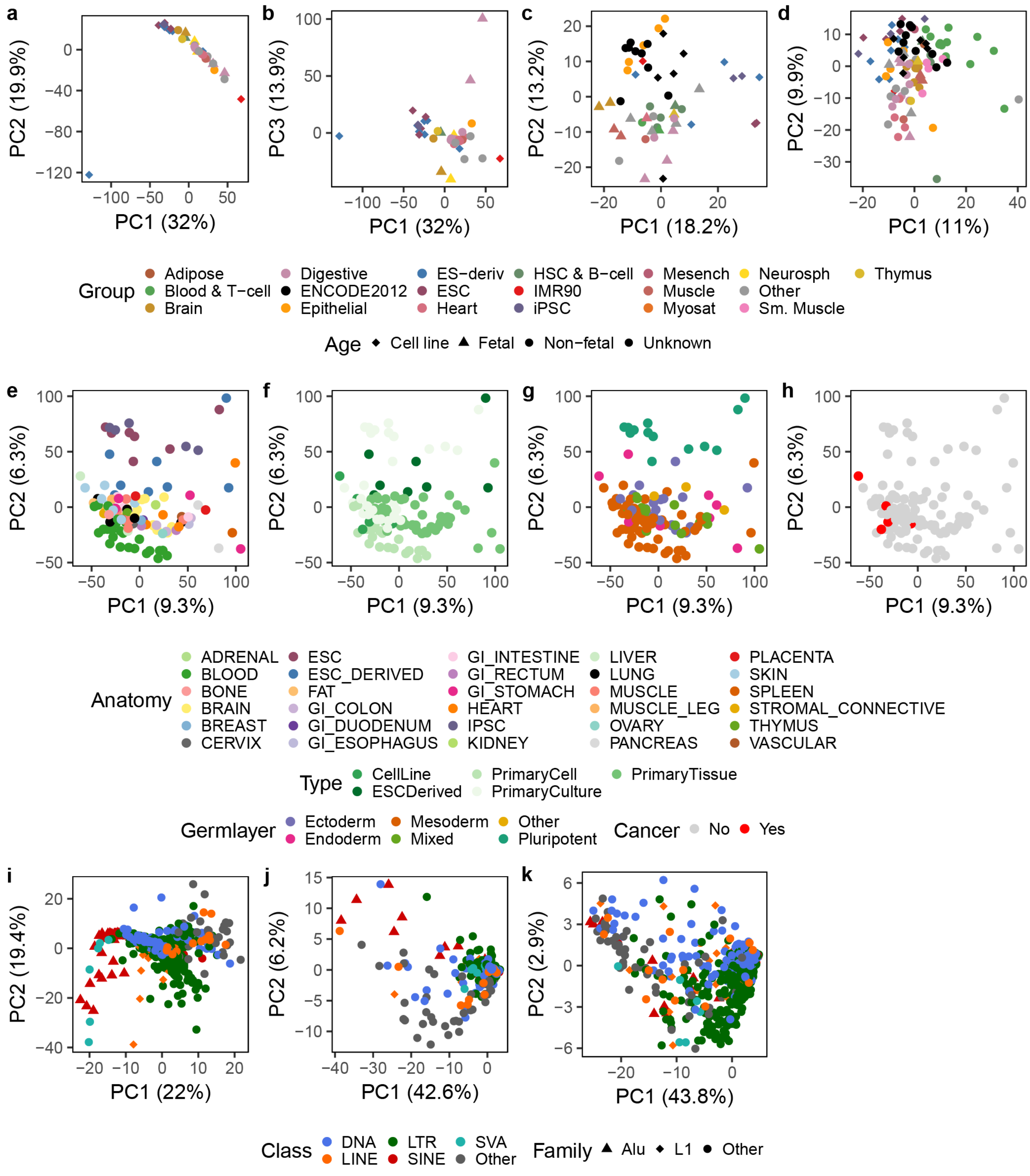

Supplementary Figure 13. PCA on enrichment of TE subfamilies in epigenetic states in Roadmap epigenomes, using additional epigenetic marks and alternative epigenome classifications. **a-k** The amount of variation explained by each PC is listed in parentheses. **a-d** PCA on Roadmap epigenomes, using as variables the LOR enrichment of each TE subfamily in **a** each methylation state (PC1 vs. PC2; n=37 epigenomes, variables are 3,656 subfamily by state combinations), **b** each methylation state (PC1 vs. PC3), **c** overlap with DHS peaks (n=53 epigenomes, variables are 937 subfamilies), **d** overlap with H3K27ac peaks (n=98 epigenomes, variables are 937 subfamilies). Each point represents an epigenome, with color based on group and shape based on epigenome age. The outlier in **a** along PC1 and PC2 is E012, which has an unusually low number of CpGs missing methylation data (see Supplementary Figure 1). The outliers along PC3 in **b** are E084 and E085. **e-h** The PCA plot from Figure 5b (PCA on Roadmap epigenomes (n=127 epigenomes), using the LOR enrichment of each TE subfamily in each chromHMM state (13,716 subfamily by state combinations) as variables), colored by alternative epigenome classifications: **e** Anatomy, **f**

Type, **g** Germ layer, and **h** whether the epigenome is a cancer cell line (Cancer). **i-k** PCA on TE subfamilies, using as variables the LOR enrichment of the subfamily in each Roadmap epigenome in **i** each methylation state (n=914 subfamilies, variables are 147 epigenome by state combinations), **j** overlap with DHS peaks, and **k** overlap with H3K27ac peaks. Each point represents a TE subfamily. Color is based on class, and shape is based on TE family (only Alu and L1 families are highlighted).

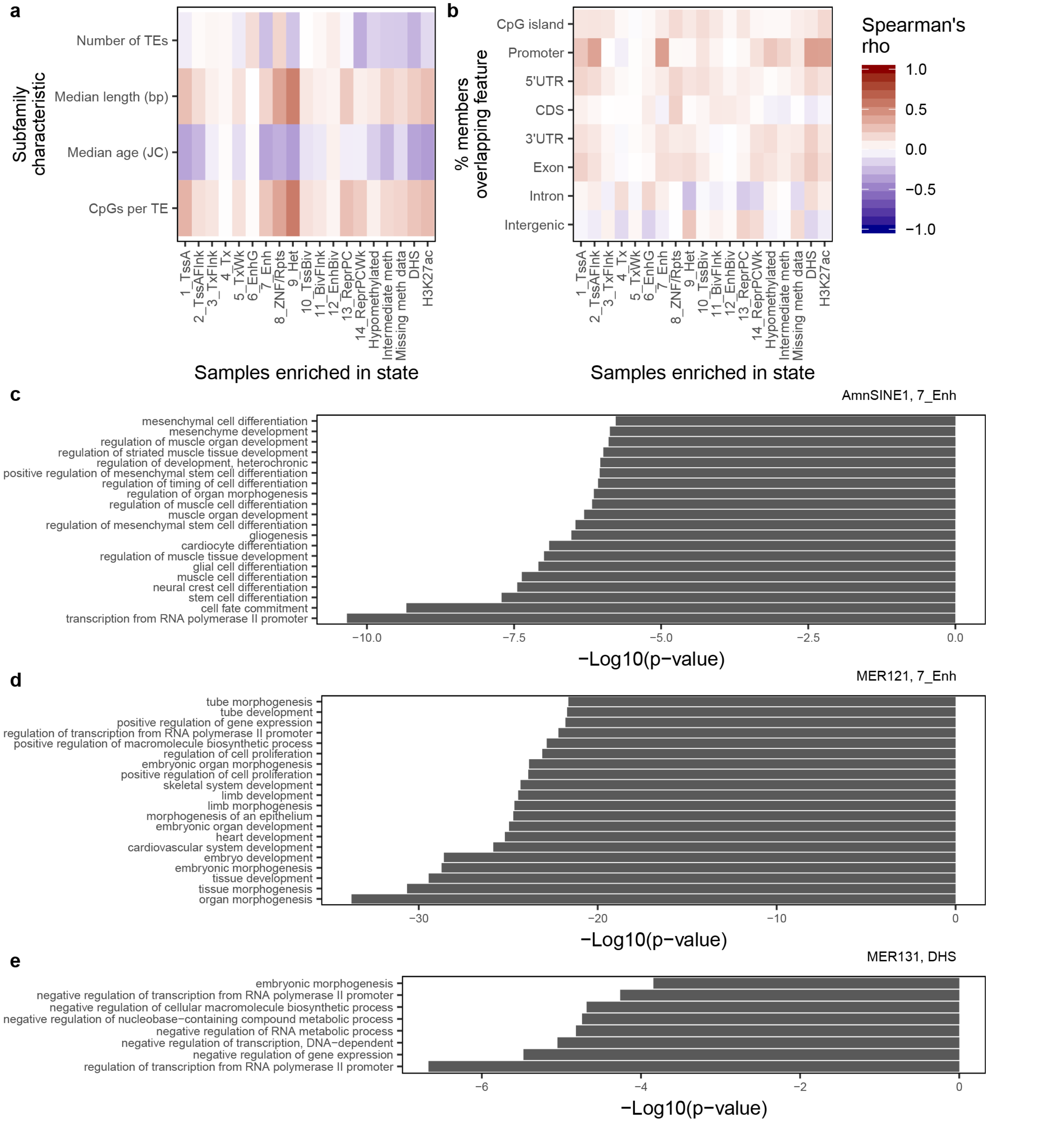

Supplementary Figure 14. Support for TE subfamilies enriched in active regulatory states. **a-b** Spearman correlation between **a** subfamily sequence features and **b** the proportion of subfamily members overlapping each genic feature and the proportion of epigenomes the subfamily is enriched in each state (chromHMM states 127 epigenomes, methylation states 37, DHS 53, H3K27ac 98), for LTR subfamilies only (n=504 subfamilies). Color represents Spearman's rho and is represented by the scale bar to the right of Supplementary Figure 14b. JC: Jukes-Cantor evolutionary distance. **c-e** The top 20 most significantly enriched GO Biological Processes (as identified by GREAT, binomial and hypergeometric test, FDR-corrected p-value < 0.05, default genomic background) for **c** the AmnSINE1 subfamily in the 7_Enh state (n=701 TEs), **d** MER121 in the 7_Enh state (n=475 TEs), and **e** MER131 overlapping DHS peaks (n=153 TEs). Enrichments are based on subfamily members annotated with the state in epigenomes where the subfamily is enriched in the state.

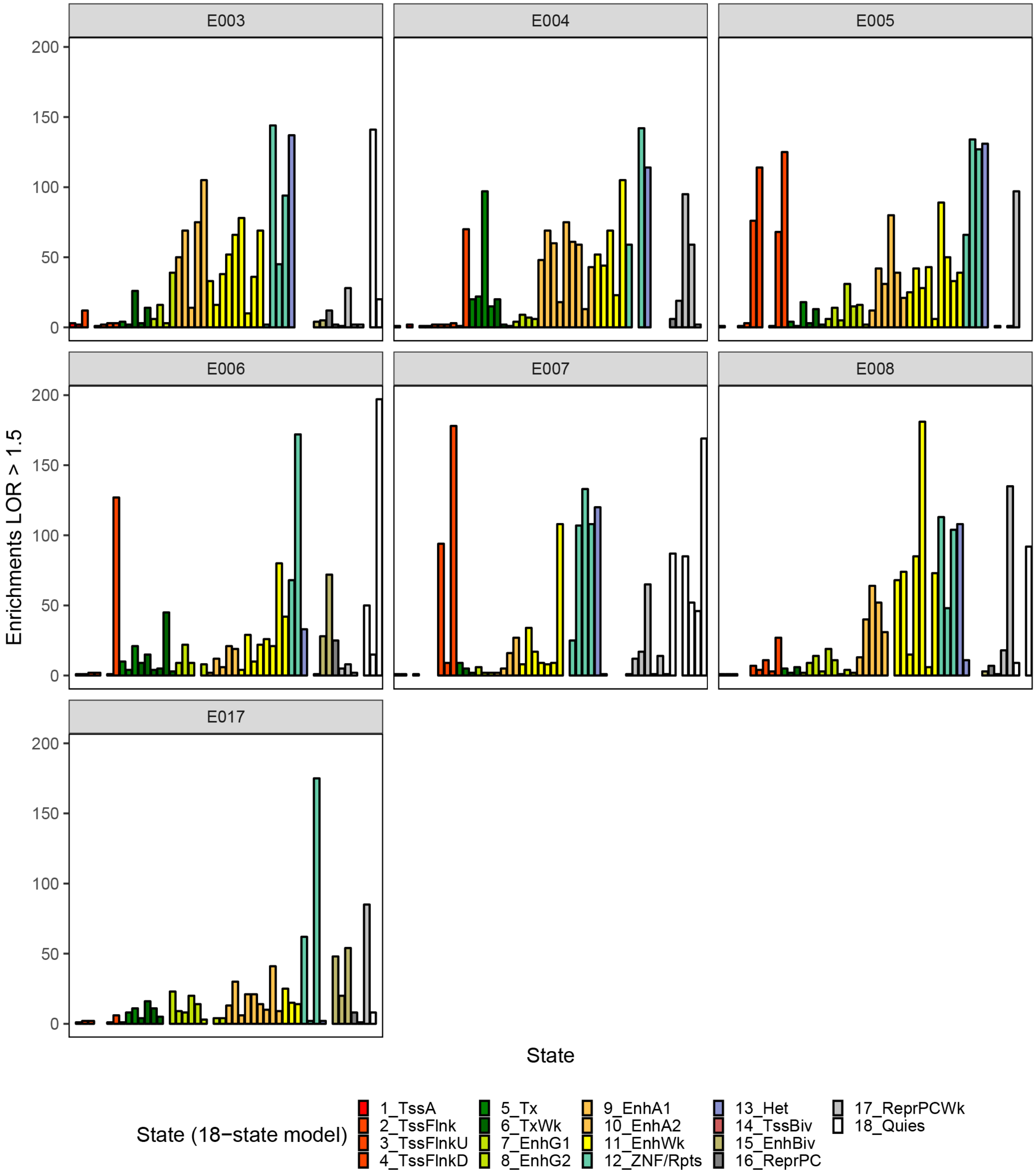

Supplementary Figure 15. Subfamily-level enrichment in epigenetic states using the 50-state chromHMM models. For each of the 7 epigenomes for which a chromHMM model was generated, the number of subfamily LOR enrichments in each state (left to right, E1-E50) with LOR > 1.5 (out of 968 subfamilies). Bar plots are colored by the corresponding 18-state model state.

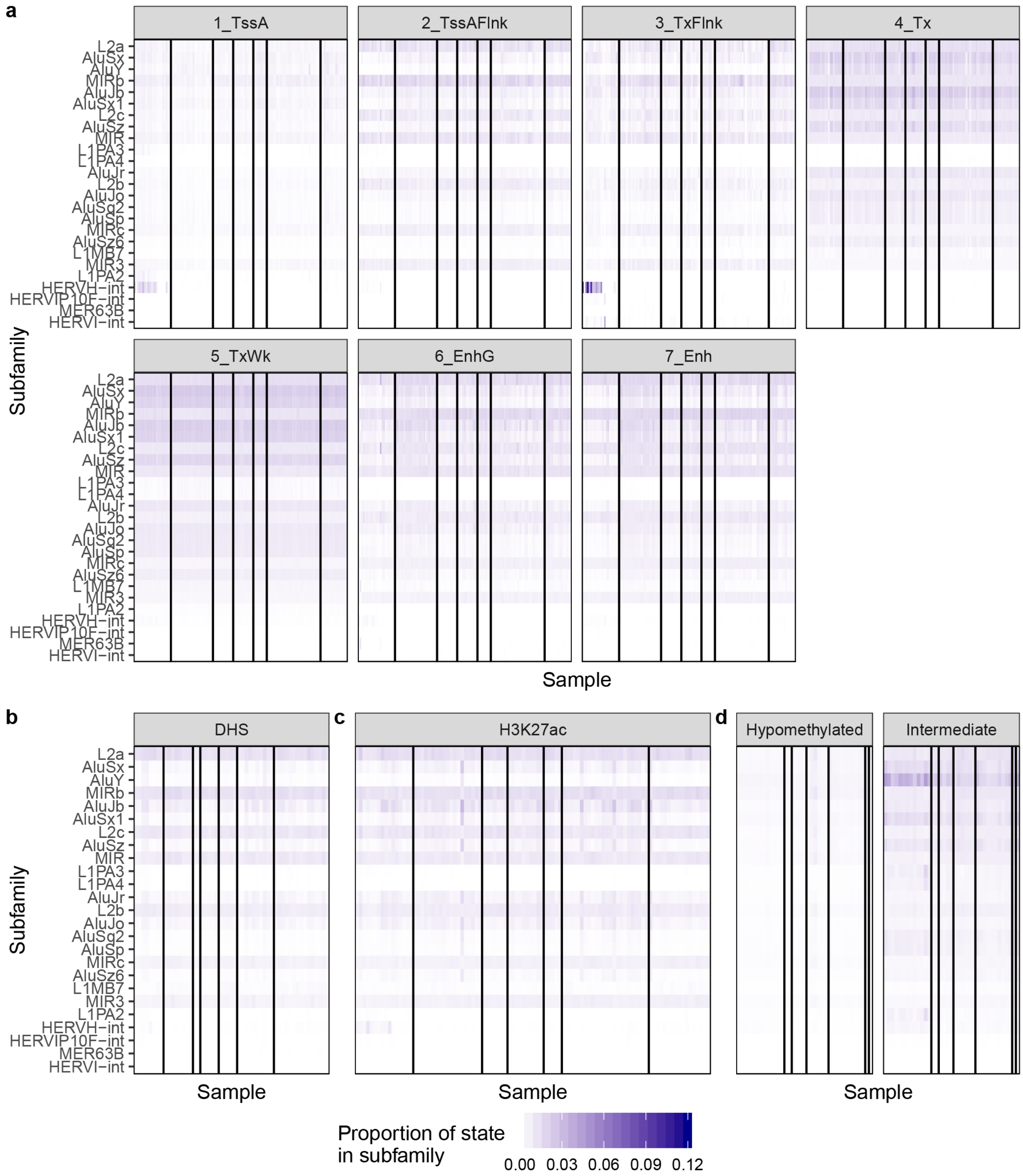

Supplementary Figure 16. Subfamilies encompassing a large proportion of an epigenetic state. **a-d** Subfamilies overlapping more than 1% of **a** an active regulatory or transcribed chromHMM state, **b** DHS or **c** H3K27ac peak summits, or **d** hypomethylated or intermediately methylated CpGs in at least one epigenome. The y-axis for **c-d** is shared with **b**. Color represents the proportion of the state within the subfamily in each epigenome (columns: chromHMM states 127 epigenomes, methylation states 37, DHS 53, H3K27ac 98), and the scale bar applies to all panels. Subfamilies are ordered by decreasing total length. All are in the top 30 subfamilies by length except HERVH-int (#66), HERVIP10F-int (#286), MER63B (#346), and HERVI-int (#449). L2a, AluSx, AluY, MIRb, AluJb, and AluSx1 comprise >1% of the genome (30,956,940 bp), and AluSx, AluY, AluJb, AluSx1, AluSz, AluSq2, AluSp, (and AluSg), comprise >1% of CpGs (282,175 CpGs). Epigenomes are ordered by group, with black vertical lines dividing ESC/iPSC/ES-deriv; Blood & T-cell/HSC & B-cell/Thymus; Brain/Neurosph; Digestive; Epithelial; Adipose/Mesench/Myosat/Muscle/Sm. Muscle/Heart/Other; and IMR90/ENCODE2012.

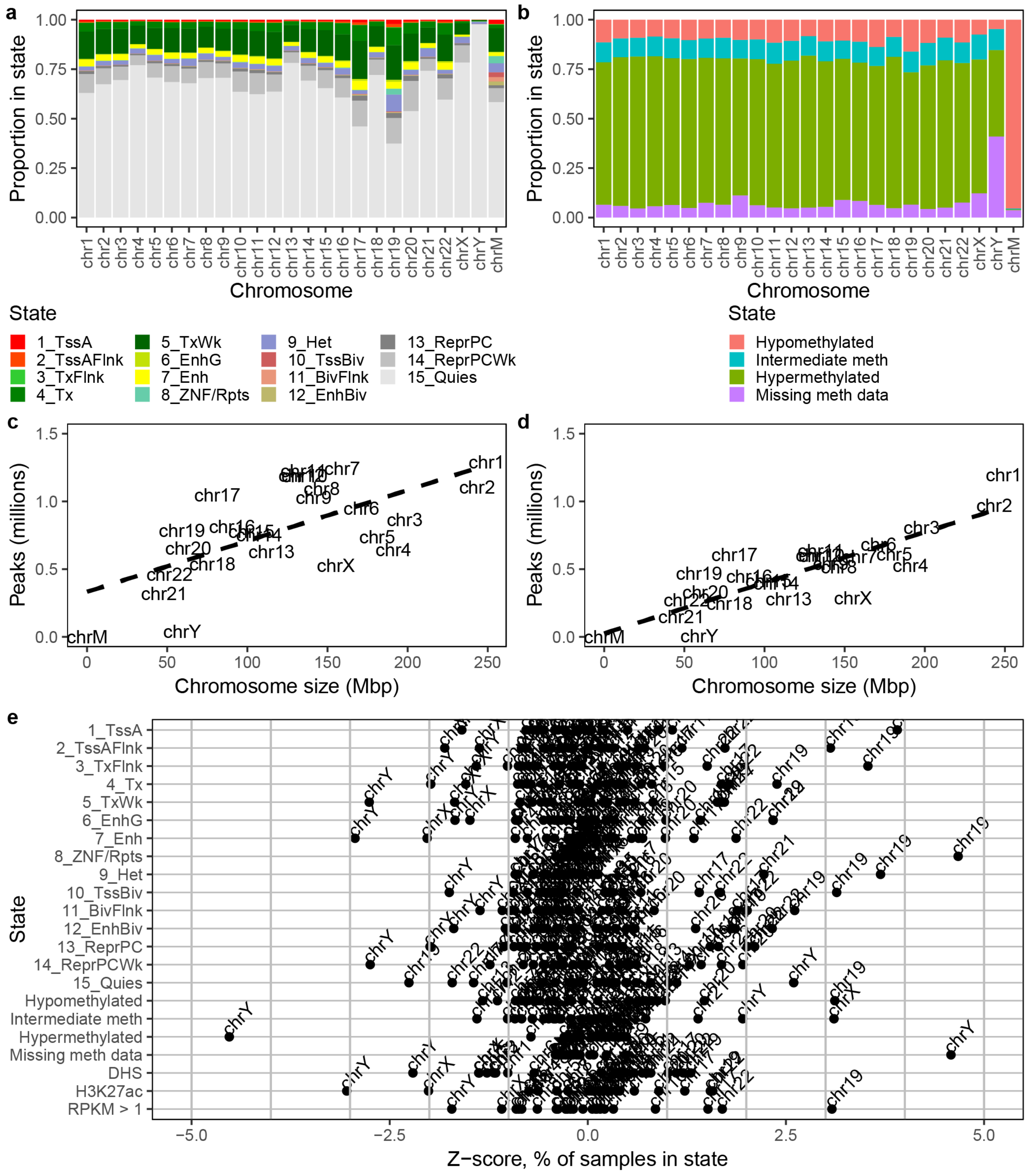

Supplementary Figure 17. Epigenetic state by chromosome. **a** Proportion of each chromosome annotated with each chromHMM state, summed across all Roadmap epigenomes (127 epigenomes). **b** Proportion of each chromosome annotated with each methylation state, summed across all Roadmap epigenomes (37 epigenomes). **c** Chromosome size vs. number of DHS peaks, summed across all epigenomes (53 epigenomes). **d** Chromosome size vs. number of H3K27ac peaks, summed across all epigenomes (98 epigenomes). **e** Z-scores for the average proportion of epigenomes a TE is annotated with the state by the chromosome where the TE is located (n=4,430,788 TEs; for methylation states, n=3,200,428 TEs with CpGs).

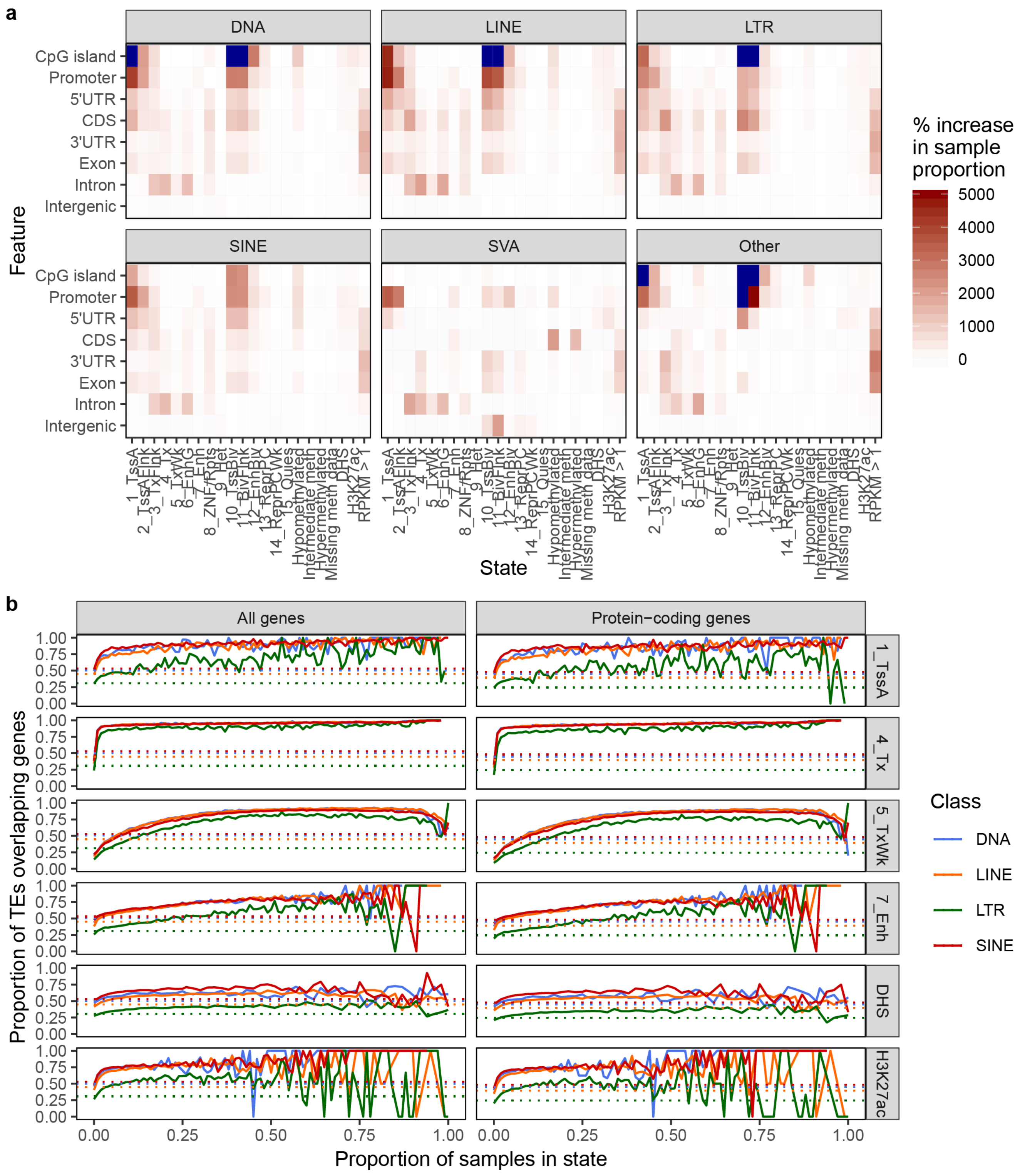

Supplementary Figure 18. Epigenetic state of individual TEs versus gene overlap. **a** Average percent increase in the proportion of epigenomes in which a TE is annotated with the epigenetic state (chromHMM states 127 epigenomes, methylation states 37, DHS 53, H3K27ac 98, expression 56) when it overlaps the RefSeq genic feature or CpG island, versus TEs that do not overlap the feature (denominator), by class see Supplementary Figure 6b). Values >5000% are in dark blue. **b** The proportion of TEs that overlap RefSeq genes (exons, introns, and promoters) versus the proportion of epigenomes in which the TE is annotated with each state (solid lines; see Figure 2c), for the four largest TE classes. Dashed lines represent the overall proportion of elements in that class that overlap genes.

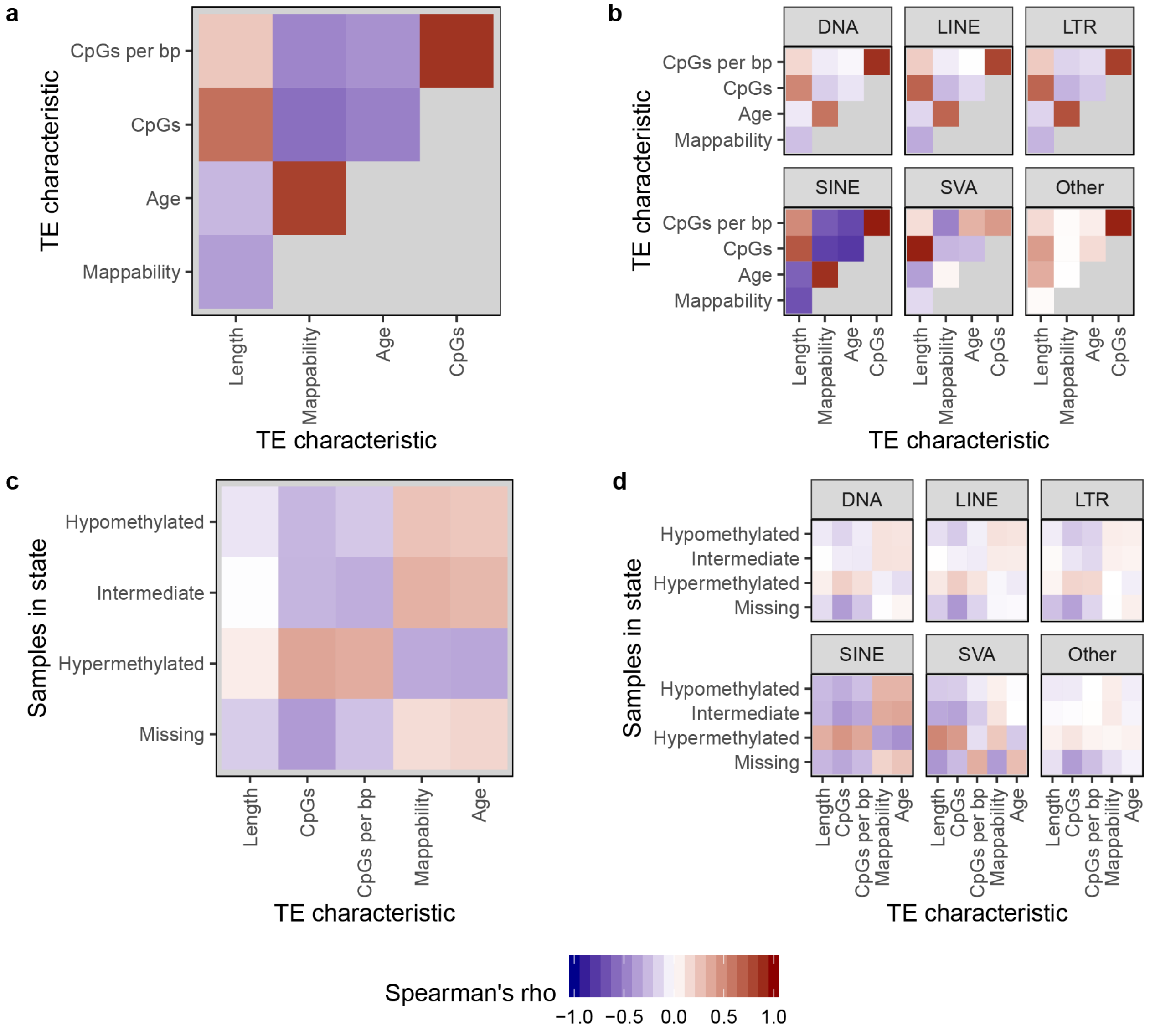

Supplementary Figure 19. Individual TE characteristics and their correlation with epigenetic state, by class. **a-d** The scale bar applies to all panels. **a** Spearman correlation between individual TE characteristics (n=4,430,788 TEs). **b** Spearman correlation between individual TE characteristics, by class (DNA n=456,948 TEs, LINE n=1,480,369, LTR n=708,210, SINE n=1,769,839, SVA n=3,608, Other n=11,814). **c** Spearman correlation between individual TE characteristics and the proportion of epigenomes the TE is in each methylation state (n=3,200,428 TEs with CpGs; 37 epigenomes). **d** Spearman correlation between individual TE characteristics and the proportion of epigenomes the TE is in each methylation state, by class (DNA n=275,140 TEs with CpGs, LINE n=952,459, LTR n=532,571, SINE n=1,430,171, SVA n=3,519, Other n=6,568).

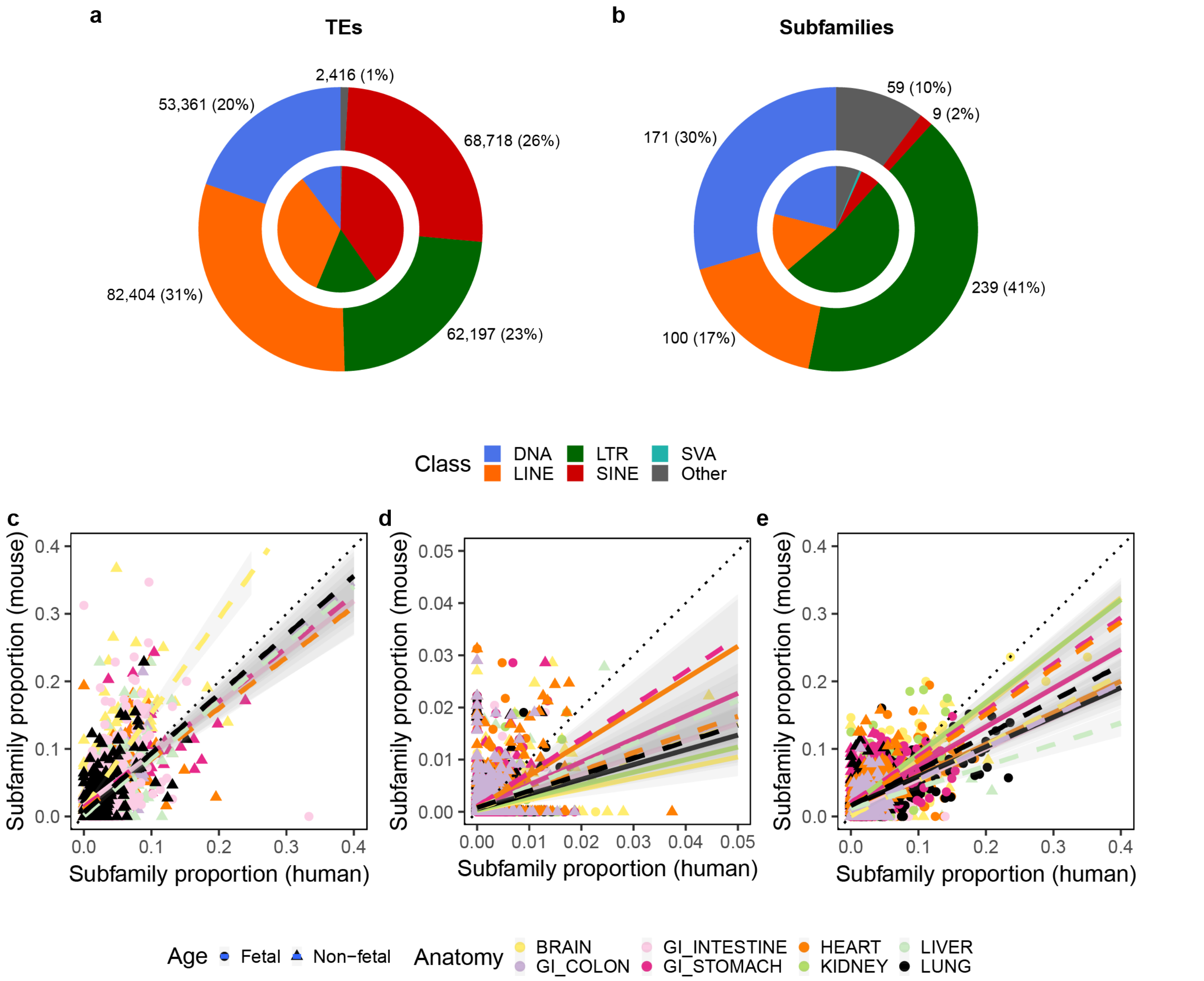

Supplementary Figure 20. Orthologous TE characteristics and subfamily epigenetic conservation. **a** The proportion of human TEs (hg19) with orthologs in mouse (mm10) in each TE class (outer circle) versus the proportion of all human TEs in each TE class (inner circle; see Supplementary Figure 3b). **b** The proportion of human TE subfamilies shared with mouse in each TE class (outer circle) versus all human subfamilies (inner circle; see Supplementary Figure 3c). **c-e** The proportion of members of each shared TE subfamily that is **c** hypomethylated, **d** in the promoter state, or **e** in an active regulatory state in matched human and mouse samples, including non-orthologous members. Only subfamilies with >30 members in both species are included (n=502 subfamilies; 30 members with CpGs for methylation analyses, n=466 subfamilies). Each point represents a subfamily in one epigenome and is colored by epigenome anatomy, while shapes correspond to human epigenome age. Lines represent a linear regression for each epigenome across all subfamilies (Fetal: solid, Non-fetal: dashed). The dotted black line represents x=y.

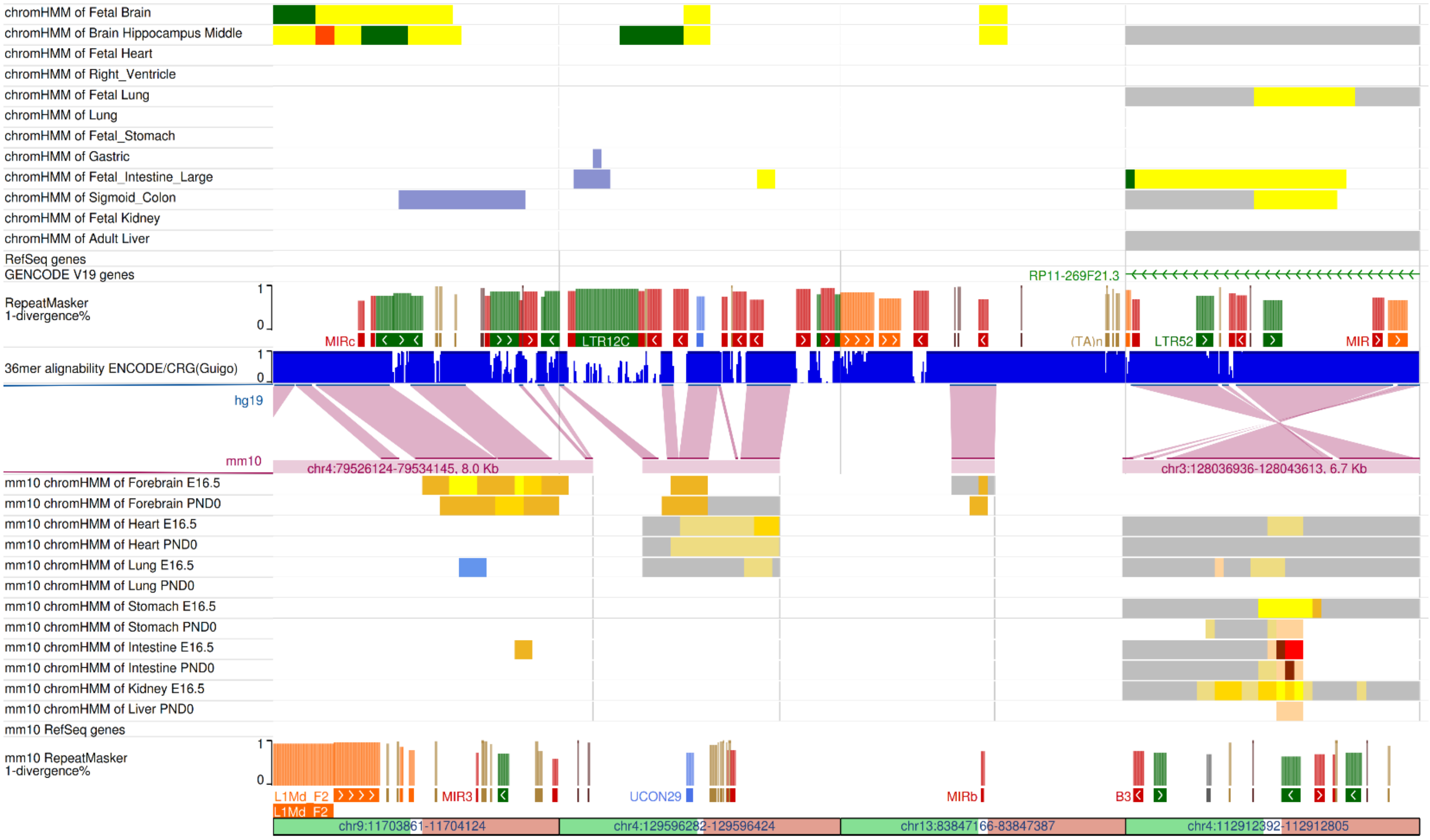

Supplementary Figure 21. Conserved tissue-specific regulatory signatures. WashU Epigenome Browser view of four intergenic human-mouse TE orthologs with shared tissue-specific epigenetic annotation in hg19 (Roadmap) and mm10 (mouseENCODE) tissues with corresponding anatomy. chromHMM state colors are as in Figure 1c (human) and Figure 7c (mouse). The position of the hg19 TEs is provided in the bottom track, while the position of the mm10 TEs is provided by the red lines indicating lift over. From left to right: MER92B (green, with a THE1D insertion), UCON29 (blue), MIRb (red), and MLT1L (green).

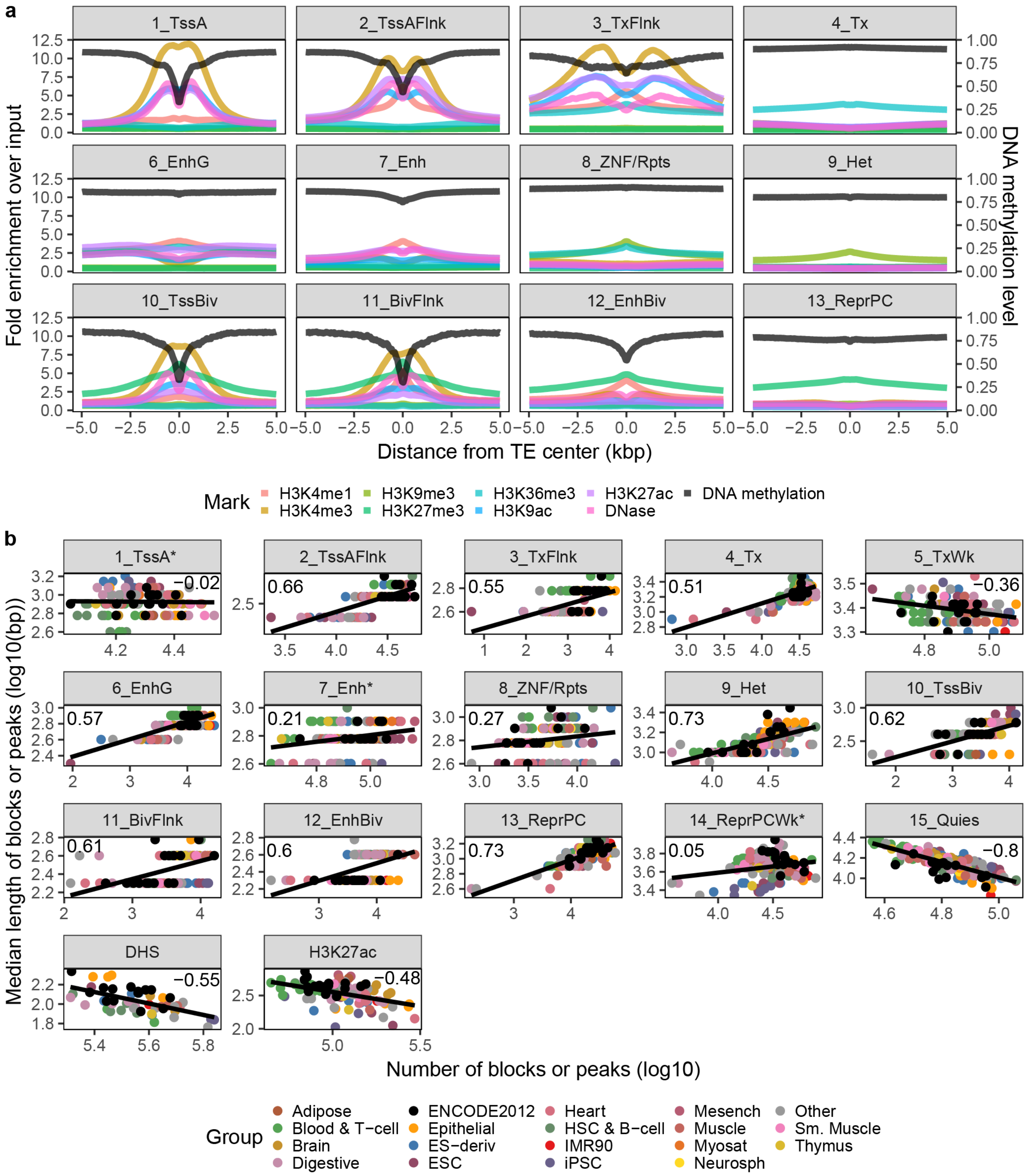

Supplementary Figure 22. Support for chromHMM annotations and state annotation size by epigenome. **a** For all TE instances in each chromHMM state, fold-enrichment ratios over input for ChIP-seq on seven histone modifications and DHS assays (left y-axis), as well as the DNA methylation level (right y-axis; black line). Marks were profiled over a 10kb region centered on the TE (see Methods). Values were calculated in bins of 50bp and represent averages of all instances of a TE in that state in an epigenome. Only epigenomes with data for that mark were included (H3K4me1, H3K4me3, H3K9me3, H3K27me3, and H3K36me3, 127 epigenomes; H3K27ac, 98; H3K9ac, 62; DHS, 53; WGBS, 37). Three chromHMM states with weak signatures are not shown. **b** Number versus the median length of chromHMM state blocks, DHS peaks, and H3K27ac peaks by epigenome. Each epigenome is represented by a circle, colored by Roadmap group. Spearman's rho between the two measures is listed on each graph. States marked with an asterisk have a Bonferroni-adjusted Spearman correlation p-value >0.05. chromHMM states n=127 epigenomes, methylation states n=37, DHS n=53, H3K27ac n=98.

Supplementary Tables

Supplementary Table 1. chromHMM state definitions for the 15-state model generated by the Roadmap Epigenomics Project

State	Description	Category	Enriched histone marks
1_TssA	Active TSS	Active regulatory	H3K4me3, (H3K4me1)
2_TssAFlnk	Flanking Active TSS	Active regulatory	H3K4me3, H3K4me1
3_TxFlnk	Transcr. at gene 5' and 3'	Active regulatory	H3K4me3, H3K4me1, H3K36me3
4_Tx	Strong transcription	Transcribed	H3K36me3
5_TxWk	Weak transcription	Transcribed	(H3K36me3)
6_EnhG	Genic enhancers	Active regulatory	H3K4me1, H3K36me3
7_Enh	Enhancers	Active regulatory	H3K4me1
8_ZNF/Rpts	ZNF genes & repeats		H3K36me3, H3K9me3, (H3K4me3)
9_Het	Heterochromatin	Repressed	H3K9me3
10_TssBiv	Bivalent/Poised TSS	Poised regulatory	H3K4me3, (H3K4me1), H3K27me3
11_BivFlnk	Flanking Bivalent TSS/Enh	Poised regulatory	H3K4me3, H3K4me1, H3K27me3
12_EnhBiv	Bivalent Enhancer	Poised regulatory	H3K4me1, H3K27me3
13_ReprPC	Repressed PolyComb	Repressed	H3K27me3
14_ReprPCWk	Weak Repressed PolyComb	Repressed	(H3K27me3)
15_Quies	Quiescent/Low		None

The description and enriched histone marks for each state are from the Roadmap Epigenomics Project publication[1]. Weakly enriched histone marks are in parentheses. Category was assigned in this paper.

Supplementary Table 2. Generalized linear models of TE epigenetic state versus class and CpG density

	**Intercept estimate**	**p-value**	**Class (SINE) estimate**	**p-value**	**CpG density estimate**	**p-value**
9_Het	1.9338411	<2e-16	-1.0527651	<2e-16	-0.0033067	<2e-16
Hypermethylated	3.367e+00	<2e-16	8.191e-02	<2e-16	2.399e-03	<2e-16

Quasi-Poisson family generalized linear models were constructed for the number of epigenomes a TE is in the 9_Het or hypermethylated states (function glm(), quasipoisson family). Class (LTR vs. SINE) and CpG density (CpGs per kbp) were included as predictors. Coefficient estimates and p-values are included for each predictor in the combined model.

Supplementary Table 3. Generalized additive models of TE characteristics or methylation state versus age

	Predictor	Intercept estimate	p-value	Jukes-Cantor estimate	p-value
SINE	Epigenomes hypomethylated	-0.0082081	<2e-16	0.1159926	<2e-16
	Epigenomes intermediately methylated	0.0004362	0.0277	0.3494058	<2e-16
	Epigenomes hypermethylated	0.9929504	<2e-16	-0.5220017	<2e-16
	Epigenomes missing methylation	0.0148215	<2e-16	0.0566033	<2e-16
	Length (bp)	0.3185340	<2e-16	-0.4026277	<2e-16
	Mappability	-0.1152084	<2e-16	2.7641406	<2e-16
	CpGs per bp	3.347e-02	<2e-16	-6.599e-02	<2e-16
	Overlap with CpG islands	-1.49226	<2e-16	-23.65068	<2e-16
Alu	Epigenomes hypomethylated	-0.0006745	5.59e-07	0.0466124	<2e-16
	Epigenomes intermediately methylated	-0.0060830	<2e-16	0.3864416	<2e-16
	Epigenomes hypermethylated	1.0006482	<2e-16	-0.5585809	<2e-16
	Epigenomes missing methylation	0.0061093	<2e-16	0.1255269	<2e-16
	Length (bp)	321.9487	<2e-16	-404.0943	<2e-16
	Mappability	-0.1085549	<2e-16	2.5753845	<2e-16
	CpGs per bp	5.073e-02	<2e-16	-2.032e-01	<2e-16
	Overlap with CpG islands	0.37531	<2e-16	-43.75426	<2e-16

Generalized additive models of TE length, mappability, CpG density, and the number of epigenomes a SINE or Alu element is in each methylation state were constructed independently using Jukes-Cantor evolutionary distance as the predictor (function gam()). Logistic regression models of the likelihood a SINE or Alu element overlaps a CpG island were constructed using Jukes-Cantor evolutionary distance (JC) as the predictor (function glm(), binomial family). All SINE elements with CpGs (n=1,430,171) or Alu elements with CpGs (n=1,105,597) were included in the models. Coefficient estimates and p-values are included for each predictor.

Supplementary Table 4. Paired Roadmap Epigenomics Project and mouseENCODE epigenomes

Roadmap epigenome (hg19)	Description	Sex; Age	ENCODE File accession (mm10), chromHMM	ENCODE File accession (mm10), WGBS	Description*
E066	Liver	Mixed; Unknown	ENCFF580WIS	ENCFF770AUO	Liver, PND0
E071	Brain Hippocampus Middle	Male; 81Y, 73Y	ENCFF520TYE	ENCFF446IJS	Forebrain, PND0
E081	Fetal Brain Male	Male/Unknown; 17GW, 17GW	ENCFF360BWA	NA	Forebrain, E16.5
E083	Fetal Heart	Mixed; 14GW, 15GW, 13GW	ENCFF850SZS	NA	Heart, E16.5
E084	Fetal Intestine Large	Male; 15GW	ENCFF027XQM	ENCFF895RRK	Intestine, E16.5
E086	Fetal Kidney	Unknown; 17GW	ENCFF750AZO	NA	Kidney, E16.5
E088	Fetal Lung	Female/Unknown	ENCFF410WGK	NA	Lung, E16.5
E092	Fetal Stomach	Female	ENCFF445BPN	NA	Stomach, E16.5
E094	Gastric	Male; 34Y	ENCFF882VQM	ENCFF051OZQ	Stomach, PND0
E096	Lung	Female; 30Y	ENCFF580YIU	ENCFF920WHY	Lung, PND0
E105	Right Ventricle	Male; 3Y, 34Y	ENCFF786MCR	ENCFF467UEZ	Heart, PND0
E106	Sigmoid Colon	Male; 3Y, 34Y	ENCFF491PKK	ENCFF644XMA	Intestine, PND0

*E: embryonic day; PND: postnatal day

Supplementary References

1 Roadmap Epigenomics Consortium *et al.* Integrative analysis of 111 reference human epigenomes. *Nature* **518**, 317-330, doi: https://www.doi.org/10.1038/nature14248 (2015).
