# Supplementary material for: The epigenomic landscape of transposable elements across normal human development and anatomy
Source: Nat Commun. 2019 Dec 10;10:5640. doi: 10.1038/s41467-019-13555-x (PMC6904449; doi:10.1038/s41467-019-13555-x)
Supplement: Supplementary file 2 — Description of Additional Supplementary Files [file 41467_2019_13555_MOESM2_ESM.pdf]

Description of Additional Supplementary Files

File Name: Supplementary Data 1
Description: TEs in an epigenetic state in all epigenomes.

File Name: Supplementary Data 2
Description: Permutation results for proportion of the state in TEs.

File Name: Supplementary Data 3
Description: Roadmap epigenome classifications.

File Name: Supplementary Data 4
Description: TE subfamilies preferentially enriched in epigenome categories.
