# Supplementary material for: The epigenomic landscape of transposable elements across normal human development and anatomy
Source: Nat Commun. 2019 Dec 10;10:5640. doi: 10.1038/s41467-019-13555-x (PMC6904449; doi:10.1038/s41467-019-13555-x)
Supplement: Supplementary file 7 — Reporting Summary [file 41467_2019_13555_MOESM7_ESM.pdf]

nature research

Corresponding author(s): Ting Wang
Erica Pehrsson

Last updated by author(s): Oct 19, 2019

Reporting Summary

Nature Research wishes to improve the reproducibility of the work that we publish. This form provides structure for consistency and transparency in reporting. For further information on Nature Research policies, see Authors & Referees and the Editorial Policy Checklist.

Statistics

For all statistical analyses, confirm that the following items are present in the figure legend, table legend, main text, or Methods section.

n/a	Confirmed	
☐	☒	The exact sample size (*n*) for each experimental group/condition, given as a discrete number and unit of measurement
☐	☒	A statement on whether measurements were taken from distinct samples or whether the same sample was measured repeatedly
☐	☒	The statistical test(s) used AND whether they are one- or two-sided<br>*Only common tests should be described solely by name; describe more complex techniques in the Methods section.*
☐	☒	A description of all covariates tested
☐	☒	A description of any assumptions or corrections, such as tests of normality and adjustment for multiple comparisons
☐	☒	A full description of the statistical parameters including central tendency (e.g. means) or other basic estimates (e.g. regression coefficient) AND variation (e.g. standard deviation) or associated estimates of uncertainty (e.g. confidence intervals)
☐	☒	For null hypothesis testing, the test statistic (e.g. *F*, *t*, *r*) with confidence intervals, effect sizes, degrees of freedom and *P* value noted<br>*Give P values as exact values whenever suitable.*
☒	☐	For Bayesian analysis, information on the choice of priors and Markov chain Monte Carlo settings
☒	☐	For hierarchical and complex designs, identification of the appropriate level for tests and full reporting of outcomes
☐	☒	Estimates of effect sizes (e.g. Cohen's *d*, Pearson's *r*), indicating how they were calculated

Our web collection on statistics for biologists contains articles on many of the points above.

Software and code

Policy information about availability of computer code

Data collection	No software was used to collect data for this study, as all data used was made publicly available by earlier publications.
Data analysis	Software used in this study includes R version 3.3.0 and R packages plyr (1.8.4), reshape2 (1.4.3), ggplot2 (3.1.0), gridExtra (2.2.1), RColorBrewer (1.1.2), scales (1.0.0), mgcv (1.8.24), extraFont (0.17), and rcompanion (2.0.10); python versions 2.7, 3.6.5, and 3.7; bedtools (2.27.1); bedops; kentsrc; kentUCSC; htslib; numpy; pandas; HOMER (4.9); and GREAT (v3). Custom code used in this study is available at https://github.com/epehrsson/TE_landscape.

For manuscripts utilizing custom algorithms or software that are central to the research but not yet described in published literature, software must be made available to editors/reviewers. We strongly encourage code deposition in a community repository (e.g. GitHub). See the Nature Research guidelines for submitting code & software for further information.

Data

Policy information about availability of data

All manuscripts must include a data availability statement. This statement should provide the following information, where applicable:

- Accession codes, unique identifiers, or web links for publicly available datasets
- A list of figures that have associated raw data
- A description of any restrictions on data availability

All datasets used in this manuscript were made publicly available as part of previous publications.
Data from the Roadmap Epigenomics Project were downloaded from the data portal, including: epigenome metadata [http://egg2.wustl.edu/roadmap/web_portal/meta.html]; chromHMM state assignments using the 15-state model, 127 epigenomes [http://egg2.wustl.edu/roadmap/data/byFileType/chromhmmSegmentations/ChmmModels/coreMarks/jointModel/final/all.mnemonics.bedFiles.tgz]; chromHMM state assignments using the 18-state model, 98 epigenomes [http://egg2.wustl.edu/roadmap/data/byFileType/chromhmmSegmentations/ChmmModels/core_K27ac/jointModel/final/all.mnemonics.bedFiles.tgz]; chromHMM state assignments using separate 50-state models, 7 epigenomes [http://egg2.wustl.edu/roadmap/data/byFileType/chromhmmSegmentations/ChmmModels/class1Models_50states/] (file format [EID]/[EID]_50_segments.bed.gz); WGBS fractional methylation, 37 epigenomes [http://egg2.wustl.edu/

roadmap/data/byDataType/dnamethylation/WGBS/FractionalMethylation.tar.gz]; DHS narrow peaks, 53 epigenomes [http://egg2.wustl.edu/roadmap/data/byFileType/peaks/consolidated/narrowPeak] (file format [EID]-DNase.macs2.narrowPeak.gz); H3K27ac narrow peaks, 98 epigenomes [http://egg2.wustl.edu/roadmap/data/byFileType/peaks/consolidated/narrowPeak/] (file format [EID]-H3K27ac.narrowPeak.gz); strand-agnostic, unnormalized mRNA signal coverage [http://egg2.wustl.edu/roadmap/data/byDataType/rna/signal/unnormalized_wig/strandagnostic/] (file format [EID].wig.gz) and normalization factors [http://egg2.wustl.edu/roadmap/data/byDataType/rna/signal/unnormalized_wig/all.EGID.N.readlength], 56 epigenomes; and histone modification ChIP-seq and DHS signal fold-enrichment ratios over input [http://egg2.wustl.edu/roadmap/data/byFileType/signal/consolidated/macs2signal/foldChange/] (file format [EID]-[mark].fc.signal.bigwig) for H3K4me1, H3K4me3, H3K9me3, H3K27me3, and H3K36me3 (127 epigenomes each), H3K9ac (62 epigenomes), H3K27ac (98 epigenomes), and DHS (53 epigenomes).
mm10 chromHMM assignments and WGBS CpG methylation levels were downloaded from the ENCODE data portal [https://www.encodeproject.org/]; see Supplementary Table 4 for unique accessions.
The source data underlying all figures is provided in the source data file (SourceData.tar.gz), except for Supplementary Figure 21.

Field-specific reporting

Please select the one below that is the best fit for your research. If you are not sure, read the appropriate sections before making your selection.

☒ Life sciences ☐ Behavioural & social sciences ☐ Ecological, evolutionary & environmental sciences

For a reference copy of the document with all sections, see nature.com/documents/nr-reporting-summary-flat.pdf

Life sciences study design

All studies must disclose on these points even when the disclosure is negative.

Sample size	All consolidated epigenomes produced by the Roadmap Epigenomics Project and included in the the 2015 publication were included in our analyses (n=127 epigenomes, some epigenetic marks are available for fewer epigenomes).
Data exclusions	No data was excluded.
Replication	Although some tissues have multiple consolidated epigenomes, no formal replicates were used.
Randomization	Randomization was not performed.
Blinding	The investigators were not blinded.

Reporting for specific materials, systems and methods

We require information from authors about some types of materials, experimental systems and methods used in many studies. Here, indicate whether each material, system or method listed is relevant to your study. If you are not sure if a list item applies to your research, read the appropriate section before selecting a response.

Materials & experimental systems

n/a	Involved in the study	
☒	☐	Antibodies
☒	☐	Eukaryotic cell lines
☒	☐	Palaeontology
☒	☐	Animals and other organisms
☒	☐	Human research participants
☒	☐	Clinical data

Methods

n/a	Involved in the study	
☒	☐	ChIP-seq
☒	☐	Flow cytometry
☒	☐	MRI-based neuroimaging
